# Six Novel Loci Associated with Circulating VEGF Levels Identified by a Meta-analysis of Genome-Wide Association Studies

**DOI:** 10.1371/journal.pgen.1005874

**Published:** 2016-02-24

**Authors:** Seung Hoan Choi, Daniela Ruggiero, Rossella Sorice, Ci Song, Teresa Nutile, Albert Vernon Smith, Maria Pina Concas, Michela Traglia, Caterina Barbieri, Ndeye Coumba Ndiaye, Maria G. Stathopoulou, Vasiliki Lagou, Giovanni Battista Maestrale, Cinzia Sala, Stephanie Debette, Peter Kovacs, Lars Lind, John Lamont, Peter Fitzgerald, Anke Tönjes, Vilmundur Gudnason, Daniela Toniolo, Mario Pirastu, Celine Bellenguez, Ramachandran S. Vasan, Erik Ingelsson, Anne-Louise Leutenegger, Andrew D. Johnson, Anita L. DeStefano, Sophie Visvikis-Siest, Sudha Seshadri, Marina Ciullo

**Affiliations:** 1 Department of Neurology, Boston University School of Medicine, Boston, Massachusetts, United States of America; 2 Department of Biostatistics, Boston University School of Public Health, Boston, Massachusetts, United States of America; 3 National Heart, Lung and Blood Institute’s Framingham Heart Study, Framingham, Massachusetts, United States of America; 4 Institute of Genetics and Biophysics, National Research Council of Italy, Naples, Italy; 5 Population Sciences Branch, National Heart, Lung and Blood Institute’s Framingham Heart Study, Framingham, Massachusetts, United States of America; 6 Department of Medical Sciences, Molecular Epidemiology and Science for Life Laboratory, Uppsala University, Uppsala, Sweden; 7 Department of Medical Epidemiology and Biostatistics, Karolinska Institutet, Stockholm, Sweden; 8 Icelandic Heart Association, Kopavogur, Iceland; 9 University of Iceland, Reykjavik, Iceland; 10 Institute of Population Genetics, National Research Council of Italy, Sassari, Italy; 11 Division of Genetics and Cell Biology, San Raffaele Scientific Institute, Milano, Italy; 12 UMR INSERM U1122, IGE-PCV “Interactions Gène-Environnement en Physiopathologie Cardio-Vasculaire”, Faculté de Pharmacie, Université de Lorraine, Nancy, France; 13 Wellcome Trust Centre for Human Genetics, University of Oxford, Oxford, United Kingdom; 14 Oxford Centre for Diabetes, Endocrinology and Metabolism, Radcliffe Department of Medicine, University of Oxford, Oxford, United Kingdom; 15 Department of Neurology, Bordeaux University Hospital, Bordeaux, France; 16 INSERM U897, Bordeaux, France; 17 University of Leipzig, IFB Adiposity Diseases, Leipzig, Germany; 18 Department of Medical Sciences, Uppsala University, Uppsala, Sweden; 19 Randox Laboratories, Crumlin, United Kingdom; 20 University of Leipzig, Department of Medicine, Leipzig, Germany; 21 Institut Pasteur de Lille, Lille, France; 22 INSEM U744, Lille, France; 23 Université Lille-Nord de France, Lille, France; 24 Section of Preventive Medicine and Epidemiology, Department of Medicine, Boston University Schools of Medicine and Public Health, Boston, Massachusetts, United States of America; 25 INSERM U946, Paris, France; 26 Université Paris Diderot, Sorbonne Paris Cité, IUH, UMR-S 946, Paris, France; Wellcome Trust Sanger Institute, UNITED KINGDOM

## Abstract

Vascular endothelial growth factor (VEGF) is an angiogenic and neurotrophic factor, secreted by endothelial cells, known to impact various physiological and disease processes from cancer to cardiovascular disease and to be pharmacologically modifiable. We sought to identify novel loci associated with circulating VEGF levels through a genome-wide association meta-analysis combining data from European-ancestry individuals and using a dense variant map from 1000 genomes imputation panel. Six discovery cohorts including 13,312 samples were analyzed, followed by in-silico and de-novo replication studies including an additional 2,800 individuals. A total of 10 genome-wide significant variants were identified at 7 loci. Four were novel loci (5q14.3, 10q21.3, 16q24.2 and 18q22.3) and the leading variants at these loci were rs114694170 (*MEF2C*, P = 6.79x10^-13^), rs74506613 (*JMJD1C*, P = 1.17x10^-19^), rs4782371 (*ZFPM1*, P = 1.59x10^-9^) and rs2639990 (*ZADH2*, P = 1.72x10^-8^), respectively. We also identified two new independent variants (rs34528081, *VEGFA*, P = 1.52x10^-18^; rs7043199, *VLDLR-AS1*, P = 5.12x10^-14^) at the 3 previously identified loci and strengthened the evidence for the four previously identified SNPs (rs6921438, *LOC100132354*, P = 7.39x10^-1467^; rs1740073, *C6orf223*, P = 2.34x10^-17^; rs6993770, *ZFPM2*, P = 2.44x10^-60^; rs2375981, *KCNV2*, P = 1.48x10^-100^). These variants collectively explained up to 52% of the VEGF phenotypic variance. We explored biological links between genes in the associated loci using Ingenuity Pathway Analysis that emphasized their roles in embryonic development and function. Gene set enrichment analysis identified the ERK5 pathway as enriched in genes containing VEGF associated variants. eQTL analysis showed, in three of the identified regions, variants acting as both *cis* and *trans* eQTLs for multiple genes. Most of these genes, as well as some of those in the associated loci, were involved in platelet biogenesis and functionality, suggesting the importance of this process in regulation of VEGF levels. This work also provided new insights into the involvement of genes implicated in various angiogenesis related pathologies in determining circulating VEGF levels. The understanding of the molecular mechanisms by which the identified genes affect circulating VEGF levels could be important in the development of novel VEGF-related therapies for such diseases.

## Author Summary

Vascular Endothelial Growth Factor (VEGF) is a protein with a fundamental role in development of vascular system. The protein, produced by many types of cells, is released in the blood. High levels of VEGF have been observed in different pathological conditions especially in cancer, cardiovascular, and inflammatory diseases. Therefore, identifying the genetic factors influencing VEGF levels is important for predicting and treating such pathologies. The number of genetic variants associated with VEGF levels has been limited. To identify new loci, we have performed a Genome Wide Association Study meta-analysis on a sample of more than 16,000 individuals from 10 cohorts, using a high-density genetic map. This analysis revealed 10 variants associated with VEGF circulating levels, 6 of these being novel associations. The 10 variants cumulatively explain more than 50% of the variability of VEGF serum levels. Our analyses have identified genes known to be involved in angiogenesis related diseases and genes implicated in platelet metabolism, suggesting the importance of links between this process and VEGF regulation. Overall, these data have improved our understanding of the genetic variation underlying circulating VEGF levels. This in turn could guide our response to the challenge posed by various VEGF-related pathologies.

## Introduction

Vascular Endothelial Growth Factor (VEGF) is secreted largely by endothelial cells and plays a key role in several physiological and pathological conditions. During growth, development, and maintenance of the circulatory system, VEGF is the principal pro-angiogenic factor and it has additionally, a neurotrophic role. High levels of circulating VEGF have been observed in individuals with various vascular diseases (myocardial infarction [1], stroke [2,3], heart failure [4], and atherosclerosis [5]), neurodegenerative conditions (age-related cognitive decline [6] and Alzheimer dementia [7]), immune inflammatory disorders (rheumatoid arthritis [8], inflammatory bowel disease [9], and Behçet’s disease [10]) and cancers (breast [11,12], uterine [13], gastrointestinal [14,15], lung [16] and prostate [17]). An increase of VEGF levels has also been found in patients with diabetes [18] and various reproductive disorders [19–21]. Reduced circulating VEGF levels have been observed in amyotrophic lateral sclerosis [22] and spinal bulbar muscular atrophy [23]. Moreover, since VEGF levels are pharmacologically modifiable, understanding the determinants of circulating VEGF could support efforts directed at risk prediction, prevention and therapy. Circulating VEGF levels are highly heritable [24–27] leading to a search for specific genetic determinants within the Vascular Endothelial Growth Factor A (*VEGFA*) gene [27–29]. Several putative candidate genes were then identified but could not be consistently replicated [10,30–41]. A genome-wide linkage study of VEGF levels identified the 6p21.1 *VEGFA* gene region as the main quantitative trait locus determining variation in VEGF serum levels [27]. Specific variants at this locus were also identified as the strongest associations in the first genome-wide association study (GWAS) of circulating VEGF levels based on data from 3 large cohort studies in this consortium, wherein two addition loci, located at 8q23.1, and 9p24.2 were also identified [42]. We have now conducted a new GWAS meta-analysis using an extended sample, the largest to date, and a deeper genomic coverage based on imputation to the 1000 genomes panel to identify additional genetic variants that explain variation in circulating VEGF concentrations.

## Results

### Characteristics of study participants

A GWAS meta-analysis of VEGF levels was performed in 16,112 individuals from 10 cohorts of European ancestry (see Materials and Methods and Section 1 in S1 Text for details): the Age Gene/Environment Susceptibility Reykjavik Study (AGES), the Cilento study (Cilento), the Framingham Heart Study (FHS), the Ogliastra Genetic Park (OGP), the Prospective Investigation of the Vasculature in Uppsala Seniors Study (PIVUS), and the Val Borbera study (VB) served as discovery cohorts; the Gioi population, the Sorbs population, the STANISLAS Family Study (SFS) and a sample of hypertensive adults (HT) served as replication cohorts. The characteristics of study participants are shown in Table 1. The mean age of the participants was 54.8 years, ranging from 30.4 years in SFS to 76.2 years in the AGES. The percentage of females in the overall sample was 54%, ranging from 37% in OGP to 64% in Sorbs. To account for differences in age distribution and gender among the studies, both age and sex were subsequently used as covariates in the association analyses. Across studies, median VEGF levels ranged from 27.0 to 393.6 pg/ml, with the lowest median levels in HT and SFS studies in which VEGF was measured in plasma rather than serum (see Section 2 in S1 Text for details). This is expected since VEGF levels are higher in serum than in plasma secondary to VEGF release from platelets during clot formation [43,44]. Differences in VEGF levels also partly reflect demographic and assay differences between the cohorts.

**Table 1:** Descriptive statistics for participating cohorts at all stages.

Study*	Sample size	Age^†^(mean±SD)	Women %(n)	VEGF(median; 25%-75%)^‡^	VEGF(min; mean; max)	Undetectable % (n)	Stage^§^
**AGES**	1548	76.16 ± 5.61	51.17 (916)	50.9; 35.3–75.3	0.2; 61.5; 715.6	0.06 (1)	1
**Cilento**	1115	51.25 ± 19.29	54.98 (613)	382.8; 232.3–591.4	16.6; 447.7; 2046.6	0 (0)	1
**FHS**	7048	51.50 ± 15.66	54.27(3825)	284.6;161.7–452.1	2.0; 342.5; 2718.0	0 (0)	1
**OGP**	897	52.82 ± 18.17	36.57 (328)	65.4; 27.9–109.2	20.8; 86.5; 2690.0	20.4 (187)	1
**PIVUS**	945	70.20 ± 0.17	50.05 (473)	187.4; 106.2–316.1	10.3; 237.6; 1167.9	0 (0)	1
**VB**	1759	55.08 ± 17.99	56.45 (993)	66.3; 13.4–137.2	2.7; 106.6; 2429.1	23.5 (414)	1
**Gioi**	470	55.50 ± 20.30	57.45 (270)	393.6; 226.5–585.8	34.3; 434.7; 1268.7	0 (0)	2
**Sorbs**	659	48.21 ± 16.26	64.04 (422)	49.3; 0–98.4	2.4; 75; 1269.4	27.8 (183)	2
**HT**	995	55.51 ± 11.12	50.45 (502)	33.3; 20.7–59.0	4.7; 47; 448.3	0 (0)	3
**SFS**	676	30.45 ± 14.03	49.85 (337)	27.0; 16.7–43.2	9.7; 35.1; 255.1	0 (0)	3

*Study names were abbreviate as Age Gene/Environment Susceptibility Reykjavik Study (AGES), Cilento study (Cilento), Framingham Heart Study (FHS), Ogliastra Genetic Park (OGP), Prospective Investigation of the Vasculature in Uppsala Seniors Study (PIVUS), Val Borbera (VB), a village included in the Cilento study (Gioi), Sorbs population (Sorbs), hypertensive adults (HT) and STANISLAS Family Study (SFS) from Biological Resources Center (BRC) Interactions Gène-Environnement en Physiopathologie CardioVasculaire” (IGE- PCV).

^†^Age at measured VEGF levels in years.

^‡^The median and interquartile range were calculated considering the individuals with undetectable levels of VEGF as having 0 pg/ml.

^§^The analysis stage in which each cohort was used.

### Meta-analysis

**Fig 1:**
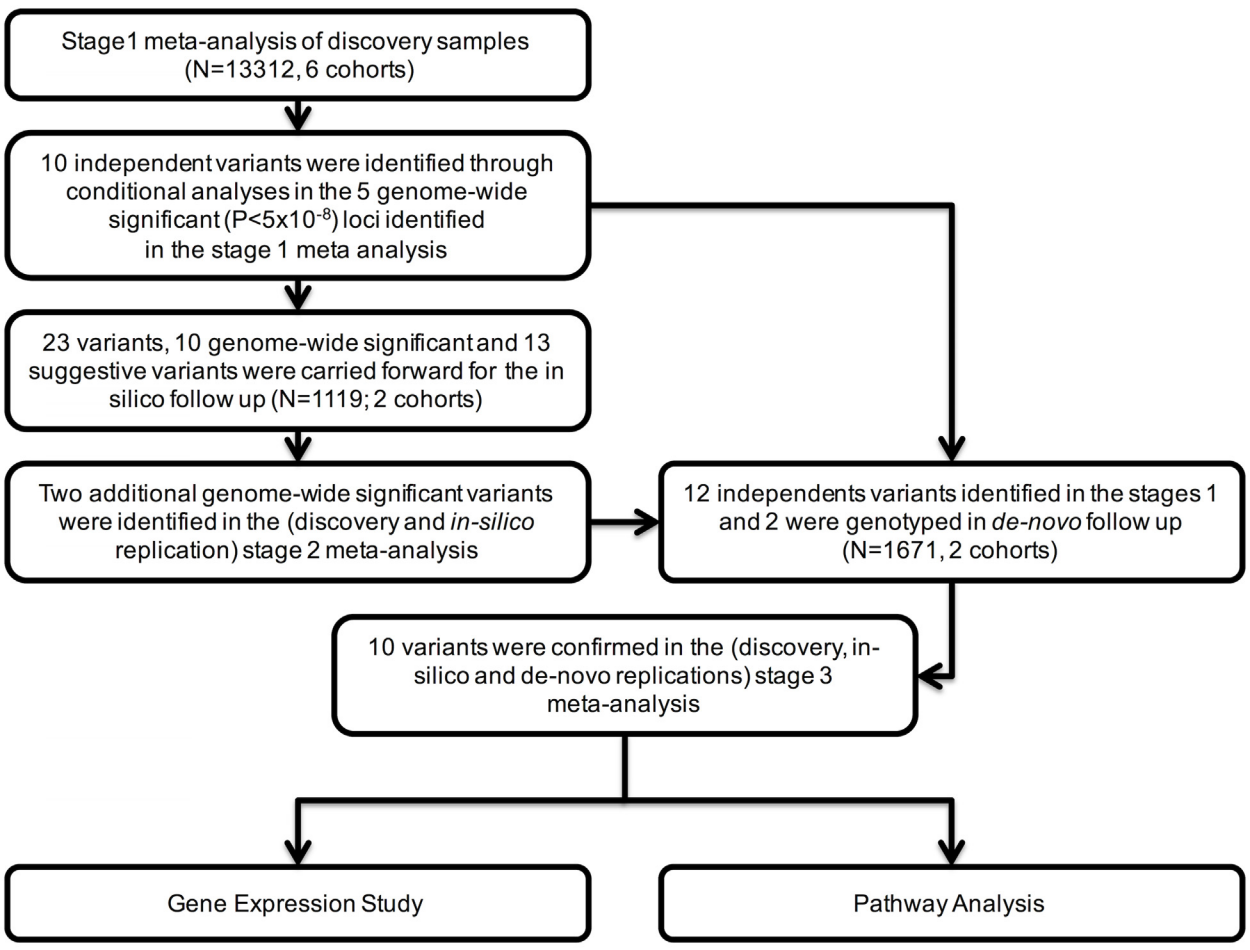
Flow chart for VEGF meta-analysis study design.

An overview of the study design is presented in Fig 1. Due to heterogeneity in the distribution of VEGF levels among the cohorts (Table 1), a sample size-weighted Z-score (rather than an inverse-variance) method was chosen for the meta-analysis. A discovery GWAS meta-analysis was carried out for 6,705,861 autosomal variants in 13,312 individuals from the six cohorts described in the “Characteristics of study participants” section (Stage 1). A Quantile-Quantile plot for the investigated variants revealed many more variants with lower observed p-values (P) than expected (S1 and S2 Figs).

There were 920 variants in 5 chromosomal regions (6p12.1, 8q23.1, and 9p24.2, which have been previously described and two novel regions at 5q14.3 and 10q21.3) that reached genome-wide significance (P<5x10^-8^) in the discovery sample (S2 Table). To identify independently associated variants within these 5 genome-wide significant genomic regions, conditional analyses were carried out in the study with the largest number of samples (FHS). This approach was selected since our use of a Z-score meta-analysis, which does not yield effect size estimates, precluded the use of aggregate results for conditional analyses. The conditional analyses revealed 10 independent signals (4 previously known and 6 novel variants). These 10 Stage 1 variants were carried forward to *in-silico* (Stage 2) and subsequent *de-novo* (Stage 3) replication.

Further, 57 variants in 13 loci were suggestively associated at 5x10^-8^<p-value<1x10^-5^. At each locus, a single independent signal was identified using a clumping procedure, and the most strongly associated variant at each of these 13 loci was also tested in the *in-silico* replication. Among them, 2 variants reached a genome-wide level of significance in the joint meta-analysis of discovery and *in-silico* replication samples and these two were also carried forward for the *de-novo* replication. So a total of 12 variants were carried forward to the de novo replication.

**Fig 2:**
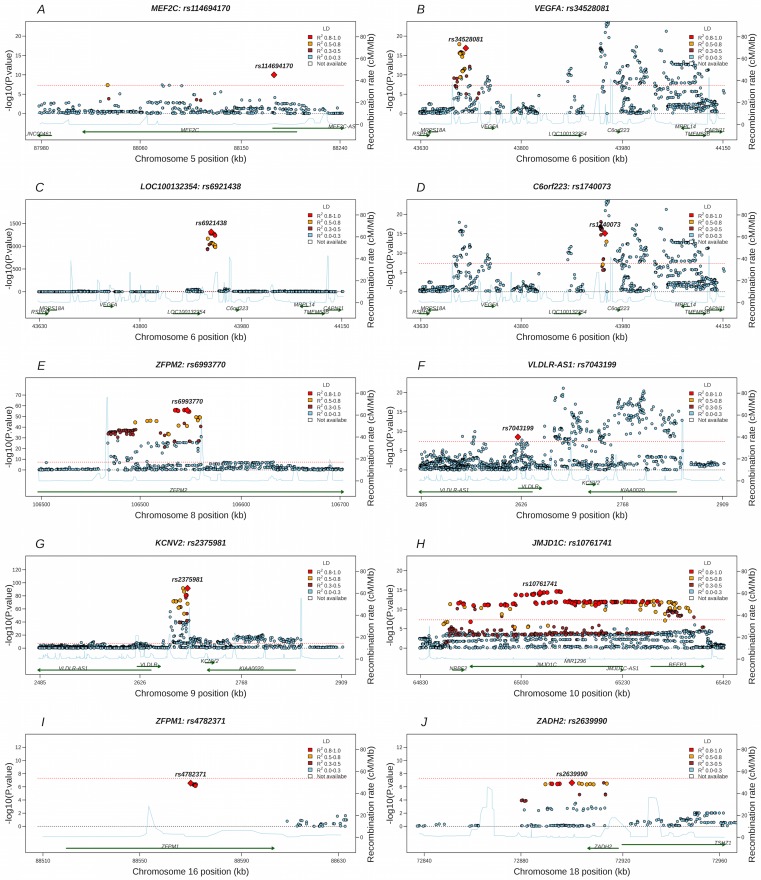
Regional plots of top 10 replicated variants in the Stage 1. Regional association plots show–log_10_ p-values for all variants ordered by their chromosomal position (build37) within regions of the 10 replicated variants. Panel A shows the 5q14.3 locus. Panels B-D show three independent signals on 6p21.1 locus. Panel E shows the 8q23.1 locus. Panels F-G show two independent signals on 9p24.2 locus. Panel H-J show 10q21.1, 16q24.2 and 18q22.3 loci respectively. The variants were color coded by R-square values with the top variant shaped in diamond. The estimated recombination rates (cM/Mb) were generated by lifting the HapMap Phase II genetic map from build35 to GRCH37 and showed in blue line. The green arrows represent the known genes in each locus. Red dotted line represents the genome-wide significant level (P = 5x10^-8^). Plots in the panels B, D and F are truncated at p-value = 10^−20^.

Overall, 10 of these 12 variants, 8 of the 10 independent variants identified in Stage 1 and the 2 variants identified in Stage 2 (combined discovery and in-silico replication), were successfully replicated in the Stage 3 meta-analysis of the combined discovery, *in-silico*, and *de-novo* replication samples (Fig 2 and Table 2).

**Table 2:** Meta-analysis result of the 10 replicated variants.

Variant rsID	Chr	Position	Coded*	Coded AF^†^	St1.P^‡^	St2.P^‡^	St3.P^‡^	Direction^§^	IV.Beta^#^	IV.SE**	IV.P^††^	Het.P^‡‡^	N.Gene^||^	Location
rs114694170^##^	5	88180196	T	0.96	1.00E-10	3.50E-12	6.80E-13	--------+-	-0.15	0.023	1.09E-11	0.034	*MEF2C*	Intron
rs34528081^##^	6	43704417	T	0.60	1.30E-17	3.20E-19	1.50E-18	---------+	-0.09	0.010	1.83E-17	0.0002	*VEGFA*	Intergenic
rs6921438	6	43925607	A	0.46	6.6E-1315	2.5E-1425	7.4E-1467	----------	-0.64	0.008	1.66E-1449	1.82E-88	*LOC100132354*	Intergenic
rs1740073	6	43947398	T	0.36	8.30E-16	8.50E-18	2.30E-17	+++--+++++	0.09	0.010	4.40E-17	1.71E-05	*C6orf223*	Intergenic
rs6993770	8	106581528	A	0.70	1.80E-56	1.30E-60	2.40E-60	++++++++++	0.16	0.010	3.83E-55	2.01E-06	*ZFPM2*	Intron
rs7043199^##^	9	2621145	A	0.21	3.10E-09	8.20E-12	5.10E-14	----------	-0.10	0.013	4.16E-14	0.49	*VLDLR-AS1*	Intron
rs2375981	9	2692583	C	0.54	2.50E-92	7.50E-100	1.50E-100	++++++++++	0.21	0.010	9.49E-99	2.00E-11	*KCNV2*	Intergenic
rs10761741^§§^^,^^##^	10	65066186	T	0.43	4.60E-15	1.10E-16	1.20E-19	++++++++++	0.08	0.009	2.99E-19	0.82	*JMJD1C*	Intron
rs4782371^##^	16	88568831	T	0.67	2.80E-07	1.20E-08	1.60E-09	----------	-0.07	0.011	1.26E-09	0.57	*ZFPM1*	Intron
rs2639990^||||^^,^^##^	18	72915551	T	0.91	2.50E-07	4.20E-08	1.70E-08	++++++++++	0.11	0.018	5.85E-10	0.81	*ZADH2*	Intron

*Coded: Coded allele.

^†^Coded AF: Coded allele Frequency.

^‡^St1.P- St2.P-St3.P: Stage1, 2, and 3 p-value.

^§^Direction: Effect direction of the coded allele on VEGF levels; the sequence of the direction follows the alphabetical order of cohorts at each stage (AGES, Cilento, FHS, OGP, PIVUS, VB, Gioi, Sorbs, HT, SFS).

^#^IV.Beta: Inverse-variance weighted meta-analysis Beta at Stage 3.

**IV.SE: Inverse-variance weighted meta-analysis standard error at Stage 3.

^††^IV.P: Inverse-variance weighted meta-analysis p-value at Stage 3.

^‡‡^Het.P: p-value for Cochran’s Q-statistic for heterogeneity at Stage 3.

^||^N.Gene: The nearest gene from the variant.

^§§^rs10761741 is a proxy variant (r^2^ = 0.97) of rs74506613.

^||||^rs2639990 is the next lowest p-value variant of rs111939830.

^##^The novel variants were identified in meta-analysis.

For these variants, an additional inverse variance-weighted meta-analysis was performed as a secondary analysis on the Stage 3 data, including the discovery and both replication cohorts. These secondary meta-analysis results, reported in the Table 2, are concordant with our original analysis results.

**Fig 3:**
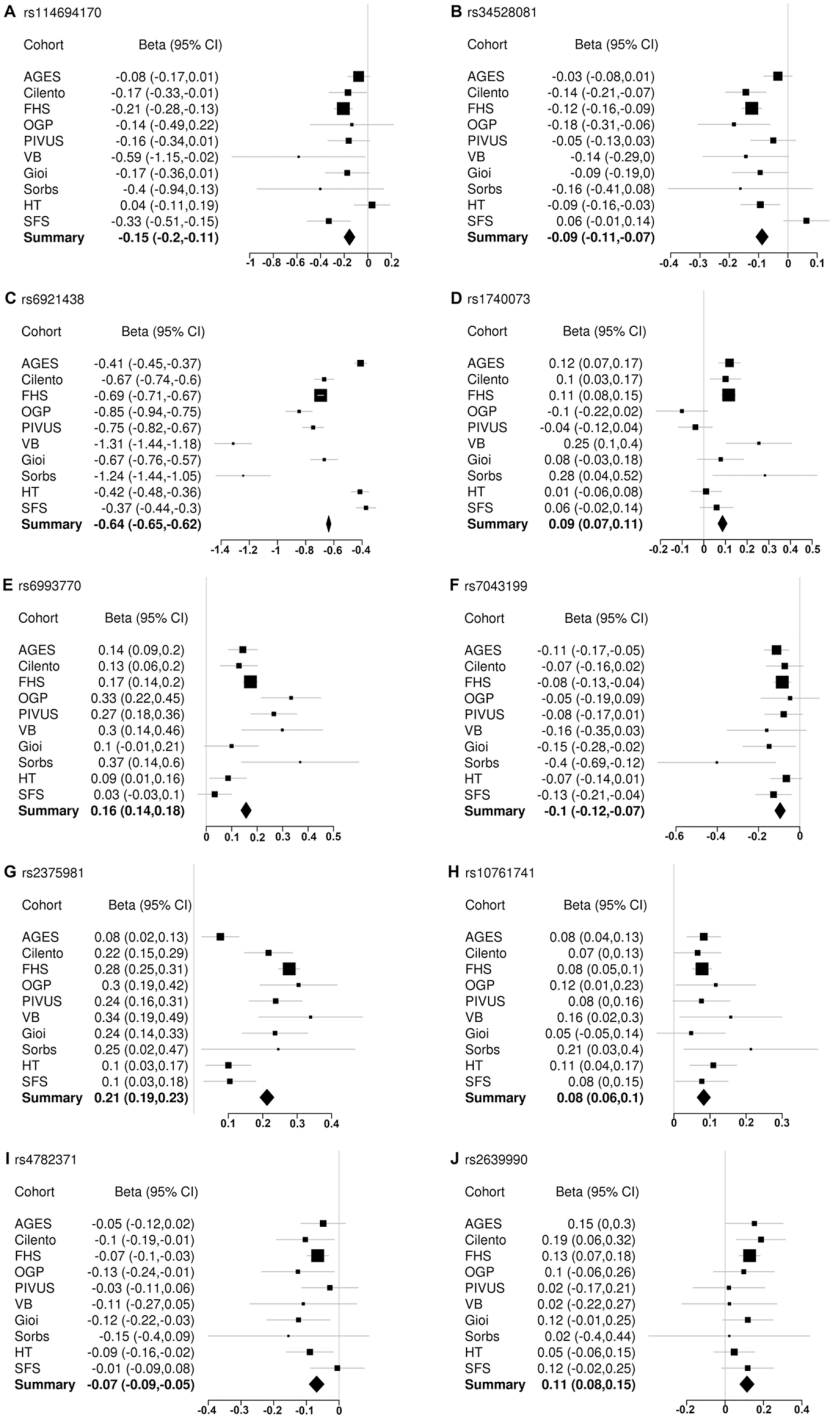
Forest plots of the 10 replicated variants. Panels A-J. The effect size (Beta) and the 95% Confidence Interval (CI) in all discovery and replication studies and the cumulative effect in the inverse-variance meta-analysis are represented for each replicated variant.

Forest plots reporting the effects of the 10 replicated variants in all the cohorts and the cumulative effect in the inverse-variance meta-analysis are shown in the Fig 3.

Among those 10 signals, 4 were located in novel chromosomal regions (5q14.3, 10q21.3, 16q24.2, and 18q22.3) and 6 (2 novel, independent variants and 4 previously known signals) were located in previously identified chromosomal regions (6p21.1, 8q23.1, and 9p24.2).

### VEGF associated signals in novel chromosomal loci

The leading SNP on chromosome 5q14.3 was rs114694170 (P = 6.79x10^-13^). This new association is located in the intronic region of the myocyte enhancer factor 2C (*MEF2C*) gene. Conditional analyses did not identify additional independent variants in the region.

In the locus on chromosome 10q21.3, the most significantly associated variant was rs74506613 (proxy rs10761741 used for in-silico replication has r^2^ of 0.97, P = 1.17x10^-19^) located within the intronic region of the jumonji domain containing 1C (*JMJD1C*) gene. Conditional analyses did not identify any other independent variants in this region.

Two additional loci reached a genome-wide significance level in the meta-analysis of the combined discovery and replication samples. At the locus on chromosome 16q24.2, the most significantly associated variant was rs4782371 (P = 1.59x10^-09^) located within the intronic region of the zinc finger protein, *FOG* family member 1 (*ZFPM1*) gene. At chromosome 18q22.3, the leading variant was rs111939830 which along with the second leading variant rs2639990 (used as proxy for de novo replication for rs111939830, r^2^ = 0.48, P = 1.72x10^-08^) was located in the intronic region of the zinc binding alcohol dehydrogenase domain containing 2 (*ZADH2*) gene.

### VEGF associated signals at previously identified loci

The most significant variant on chromosome 6p21.1 was rs6921438 (P = 7.39x10^-1467^), already identified in the previous GWAS [42]. Two additional independent variants were also identified at this locus after conditional analyses. One was rs1740073 (P = 2.34x10^-17^) which was in LD with rs4416670 reported in the previous GWAS (r^2^ = 0.15) [42]. Although the LD between these two SNPs is relatively low, rs4416670 and rs1740073 are in close physical proximity (3055 base-pair distance) and conditional analysis confirmed that rs1740073 eliminated the signal of rs4416670 (P = 4.16x10^-21^; before adjusting for rs1740073, P = 0.727; after adjusting for rs1740073), hence we believe the two SNPs, rs1740073 and rs4416670, both represent a single locus of genetic variation. This rs1740073 SNP is located about 22Kb downstream from rs6921438 and both are located upstream of the gene *C6orf223*, which encodes an uncharacterized protein. The other independent variant identified, about 221kb distant from the main signal rs6921438, was rs34528081 (P = 1.52x10^-18^), a novel variant, located upstream of the *VEGFA* gene and the mitochondrial ribosomal protein S18A (*MRPS18A*) gene. The values of r^2^ between the 3 variants at 6p21.1 are extremely low (rs6921438-rs1740073 = 0.01, rs6921438-rs34528081 = 0.007, rs1740073-rs34528081 = 0.01), suggesting that the 6p21.1 region has 3 independent variants that modulate circulating VEGF levels.

The leading variant identified on chromosome 8q23.1 was rs6993770 (P = 2.44x10^-60^). This SNP, located within an intron of the zinc finger protein multitype 2 (*ZFPM2*) gene, was already known to be associated with circulating VEGF levels [42].

On chromosome 9p24.2 the most significantly associated SNP was rs2375981 (P = 1.48x10^-100^, which is in strong LD with rs10738760 (r^2^ = 0.81) reported in the previous GWAS [42]). This variant lies downstream of the very low-density lipoprotein receptor (*VLDLR*) and upstream of the potassium voltage-gated channel subfamily V member 2 (*KCNV2*) genes. One novel independent signal also found in this region using conditional analyses was rs7043199 (P = 5.12 x10^-14^) located about 71kb upstream of rs2375981, in the *VLDLR-AS1* gene and upstream of the *VLDLR* gene. No LD exists between the two variants (r^2^ = 0.0008). Thus, in the 9p24.2 region, there are 2 independent variants able to influence VEGF levels.

### Explained variance

A genetic score was calculated for each individual using information on the 10 VEGF replicated variants. This genetic score explained 52% of the observed variability in circulating VEGF levels in FHS. The proportions of variance in circulating VEGF explained by these 10 replicated variants in PIVUS, Cilento, AGES, VB, HT, and SFS are 48%, 46%, 24%, 24%, 21% and 19%, respectively. The observed differences in the proportion of variance explained might be due to heterogeneity in effect sizes of some SNPs related to the trait variability in distribution of VEGF levels across the cohorts (Table 2). Accordingly, the explained variability is similar in the cohorts where a similar distribution of VEGF levels was observed (Table 1).

### Functional element analysis in associated loci

To identify putative functional elements at the associated loci, ENCODE data related to chromatin modifications and hypersensitivity DNAse sites (DHSs) included in HaploReg [45] were analyzed. Among the 10 replicated variants and their 126 proxies (r^2^>0.8), 16 variants were located in regions reported as DHSs in 5 or more different cell lines. Among these 16, 11 variants (rs114694170 on chromosome 5p14.3, rs6993770 on chromosome 8q23.1, rs7043199 on chromosome 9p24.2, 5 proxies of rs74506613 on chromosome 10q21.3 and 3 proxies of rs4782371 on chromosome 16q24.2) were also located in a promoter and/or enhancer histone mark. These results suggest a potential functional role of these variants.

### Gene expression analysis

A large database assembled by one of the authors (AJD) that included eQTL association results from 61 studies (detailed Section 3 in S1 Text) was queried for the 10 replicated variants identified in the GWAS and their 126 proxies (r^2^>0.8). Eighty-four variants in three loci (1 replicated variant and 83 proxies of two additional replicated variants) were found in the database. The variant rs6993770 on chromosome 8q23.1 was a *trans* eQTL for the *CXCL5* gene; rs609303 (proxy of rs111939830) on chromosome 18q22.3 was a *cis* eQTL for the *TSHZ1* gene. On chromosome 10q21.3 82 proxies for rs74506613 were identified: 2 variants were *trans* eQTL for 6 genes (*AQP10*, *CXCL5*, *GUCY1A3*, *ITGA2B*, *MYL9*, and *NRGN*) and 81 were *cis* eQTLs for 3 genes (*JMJD1C*, *NRBF2* and *REEP3*); one variant rs10761779 is both a trans and cis eQTL. All 84 variants identified as eQTL in this search are listed in S3 Table.

### Biological pathway analysis

In order to identify biological pathways involved in the modulation of VEGF protein levels two pathway analysis approaches were applied. MAGENTA software [46] was applied to the Stage 1 meta-analysis results, to identify the known biological pathways most strongly represented among all the variants associated with circulating VEGF concentrations (see Materials and Methods). Overall, 3,216 biological pathways (with at least 10 genes) and 168,932 genes were examined. This pathway analysis identified 18 biological pathways, 3 molecular functions and 2 cellular components significantly associated with VEGF levels at a nominal Gene Set Enrichment Analysis (GSEA) p-value ≤0.01. Among these, only the ERK5 pathway reached statistical significance after correction for multiple testing (FDR threshold of 0.05).

**Fig 4:**
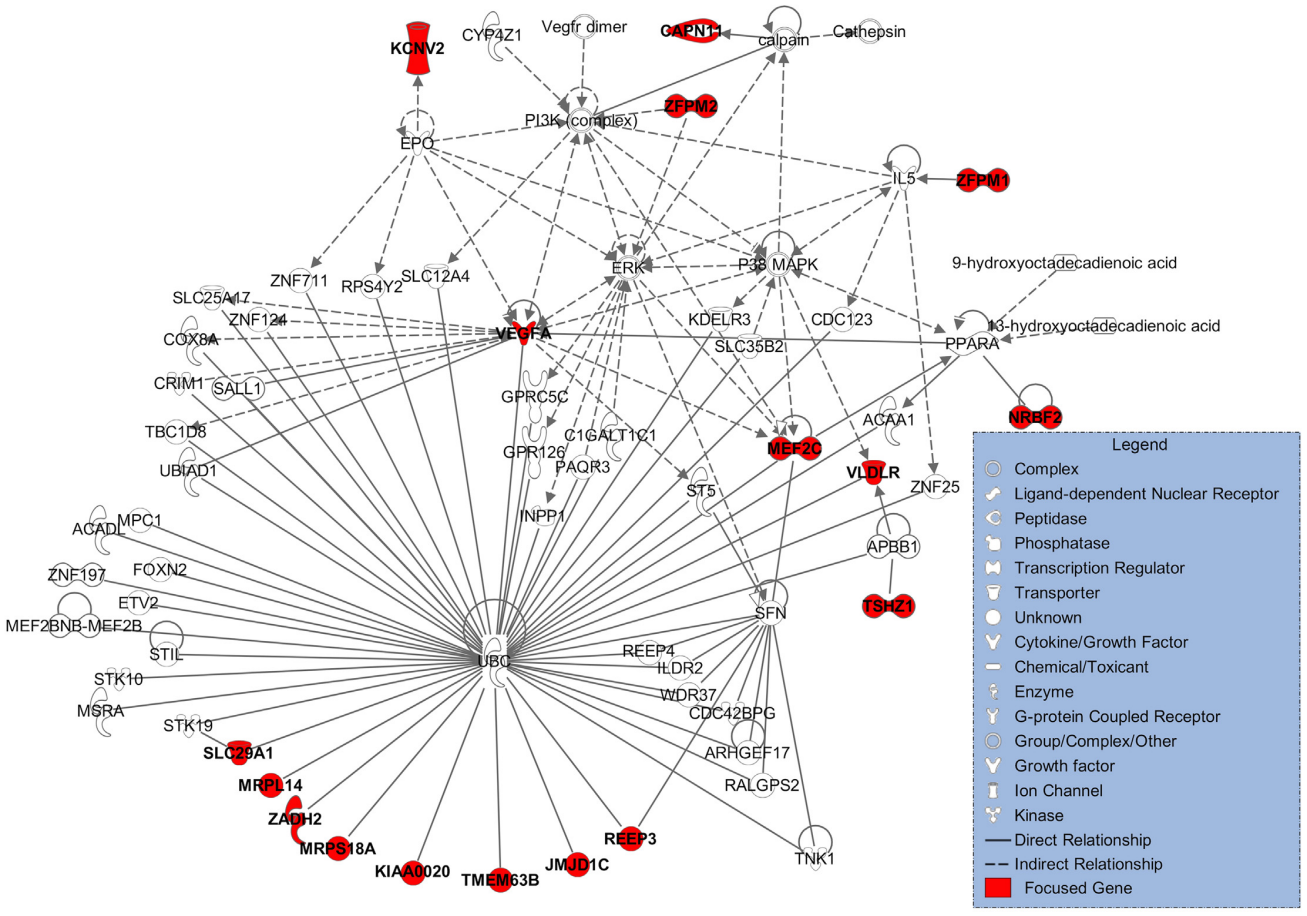
Network generated by IPA using 26 genes in replicated loci. Edges describe the characteristics of relationship between the two nodes. Lines between two genes denote verified interaction. The shapes of the nodes represent the functional level of the gene product.

The Ingenuity Pathway Analysis software (IPA, www.qiagen.com/ingenuity) was used to explore functional relationships between genes in the VEGF associated loci. A total of 26 genes located at and adjacent to the 10 replicated variants were selected as focus genes for IPA analysis (S4 Table). Among them, 17 genes were found to be biologically linked in a unique network of 70 molecules as shown in Fig 4. The associated functions for this network were organism development, especially early embryonic and later cardiovascular system development. The probability that 17 genes would be linked in a randomly designated set of 26 genes using data from the Global Molecular Network was 1.0x10^-42^. Thus, it appears extremely unlikely that this network has been identified purely by chance.

## Discussion

In this GWAS meta-analysis of circulating VEGF levels, we identified 10 independent variants located in 7 chromosomal loci; 4 of those variants had been described in a previous GWAS [42]. We now describe 6 novel variants, 4 of which were in newly identified chromosomal regions (5q14.3, 10q21.3, 16q24.2, and 18q22.3) whereas 2 were identified through conditional analyses at previously described loci (6p21.1 and 9p24.2). These 10 variants explain about 52% of VEGF phenotypic variance in the largest cohort in this study, with the 6 novel variants increasing the explained variance by 4% compared to the 48% described by Debette et al. for the 4 previously identified loci [42]. This increase represents a valuable addition to the proportion of variance explained when compared to the results obtained from GWAS of other complex traits [47–50].

The newly identified regions include many interesting and plausible candidate genes with angiogenic and neurotrophic roles.

The leading variant on chromosome 5 was located within an intron of the *MEF2C* gene. This protein has a demonstrated role in cardiac myogenesis, morphogenesis and in vascular development. *MEFC2* knock out is embryonically lethal due to cardiac and vascular abnormalities. *MEFC2* also supports cortical development and variants in this region have been associated with severe neurodevelopmental problems in humans such as developmental retardation, cerebral malformations [51,52], stereotypic movements and epilepsy. *MEF2C* was also reported to be associated with retinal vascular caliber in the Cohorts for Heart and Ageing Research in Genomic Epidemiology (CHARGE) consortium [53], which is particularly interesting given the known role of *VEGF* in proliferative retinopathy and macular degeneration. *MEF2C* may be a transmitter of *VEGF* signaling and has been shown to be regulated by *VEGF in-vitro*, as a key mediator [54].

The leading variant on chromosome 10 was located in an intronic region of *JMJD1C*, a protein-coding gene with an intriguing role in many biological processes ranging from platelet and endothelial cell function to DNA repair [55]. Thyroiditis [56] and fatty liver disease [57] have been associated with this gene. A GWAS of plasma liver enzymes revealed an association of rs7923609 (P = 6.0x10^-23^, *G* = risk allele) with elevated enzyme levels indicating abnormal liver function. Interestingly, this SNP also showed an association with *VEGF* levels in our study (P = 1.15x10^-12^) with the G allele associated with higher levels [58]. In a mouse model, it was noted that *VEGF* promotes proliferation of hepatocytes through reestablishment of liver sinusoids by proliferation of sinusoidal endothelial cells; thus *VEGF* may mediate the genetic association observed [59] between *JMJD1C* variants and hepatic steatosis.

*JMJD1C* and *MEF2C* genes were found associated to platelet count and volume in a European ancestry GWAS [49]. Further, a variant (rs7896518, P = 2.93x10^-15^) located in an intron of the *JMJD1C* gene showed an association with platelet counts (P = 2.3x10^-12^) in an African American GWAS [60]. In a second European ancestry GWAS of platelet aggregation another SNP in the same gene, rs10761741, showed an association with epinephrine-induced platelet aggregation with the T allele being associated with greater aggregation [61]. Interestingly, this *T* allele of rs10761741 was also associated with higher circulating VEGF levels (P = 7.10x10^-15^). Because both platelets and VEGF play important roles in the development of atherosclerosis and arterial thrombosis, investigating the intricate relationships among platelet, VEGF, and *JMJD1C* might identify novel drug targets and biological pathways implicated in atherosclerosis and arterial thrombosis.

In a GWAS of serum androgen levels in European men a variant (rs10822184) in *JMJD1C* reached genome-wide significance (P = 1.12x10^-8^) with the *C* allele being associated with lower levels [62]. This variant was also associated with higher circulating VEGF levels (P = 4.06x10^-11^). Further, in a GWAS of sex hormone-binding globulin, the *T* allele of a variant in *JMJD1C* (rs7910927) was associated with a decrement of sex hormone-binding globulin concentrations (P = 6.1x10^-35^) [63]. This *T* allele was also associated with a decrement of VEGF levels (P = 1.31x10^-12^). Sex hormones influence VEGF levels [64] thus suggesting a hormone-dependent VEGF production mediated by *JMJD1C*.

The leading variant in chromosome 18 was located in an intergenic region downstream of the *ZADH2* gene and upstream of the Teashirt Zinc Finger Homeobox 1 (*TSHZ1*) gene and a variant in strong LD with the lead SNP regulates expression of the latter gene. Both genes have been reported as candidate genes for congenital vertical talus [65]. *TSHZ1* has been associated with increased expression in Juvenile Angiofibroma (JA) [66]. Because VEGF is secreted by JA, and VEGF contributes to vascularization in JA [67], the investigation of relationships among *TSHZ1*, JA, and VEGF might lead to a new therapy for JA.

The top variant in chromosome 16 was located in an intron of the *ZFPM1* gene. The *ZFPM1* gene is also known as Friend of *GATA1* (*FOG1*) gene and is related to *ZFPM2*, which was identified in our previous meta-analysis [68]. Both proteins are transcription factors that play a role in the development of the heart and coronary vessels. Further, a mutation in the N-finger of the *GATA1* gene, abrogating the interaction between *GATA1* and *FOG1*, showed associations with X-linked macro-thrombocytopenia, non-X-linked thrombocytopenia and dyserythropoiesis [69]. It is possible that the observed association between *ZFPM1* and serum VEGF levels was partly driven by variations in platelet counts.

Biological pathway exploration using IPA showed that the Ubiquitin C (*UBC*) gene directly interacted with 10 of the focus genes. The encoded protein is a polyubiquitin precursor [70]. This gene has been associated with progressive accumulation of ubiquitinated protein inclusions in neurodegenerative disorders that involve dysfunction of the ubiquitin-dependent proteolytic pathway [71] and with verbal memory performance [72]. The *UBC* gene might play an important role in the association between variants and circulating VEGF serum as either mediator or confounder. However, a direct role for the *UBC* gene in determining circulating VEGF levels was not identified and none of the variants within 60kb of the *UBC* gene were associated with circulating VEGF level even at a nominally significant level.

Gene set enrichment analysis revealed the ERK5 pathway as significantly enriched for VEGF associations. ERK5 pathway is involved in multiple processes, such as cell survival, anti-apoptotic signaling, cell motility, differentiation, and cell proliferation [73,74]. ERK5 is also involved in the angiogenic process, where it acts as regulator of VEGF expression [75,76]. More recently it has been reported that this molecule is expressed on the platelet surface, and acts as platelet activator in ischemic conditions, such as after a myocardial infarct [77].

Based on eQTL analysis, we observed that 3 of the replicated variants were themselves, or in strong LD with, variants acting as *cis* and/or *trans* eQTLs on different genes. In particular, among those identified as trans-regulated genes, there were some very interesting candidates.

The C-X-C motif chemokine 5 (*CXCL5*) gene was a trans-regulated gene for 3 variants in two VEGF associated regions (rs6993770 on 8q23.1 and 2 proxies of rs74506613 on 10q21.3). It encodes a protein that through the binding of the G-protein coupled receptor chemokine (C-X-C motif) receptor 2, recruits neutrophils [78,79], promotes angiogenesis [80] and is thought to play a role in cell proliferation, migration, and invasion in different types of cancer [81–85]. *CXCL5* acts by activating several angiogenic signaling pathways, some of which, including JAK/STAT [86] and Src family kinases [87] pathways, are also activated by VEGF. Given the involvement of the two genes in the same pathways, it is conceivable that they could be co-regulated.

The *GUCY1A3* gene encodes the alpha-3 subunit of the Soluble Guanylate Cyclase (sGC), an heterodimeric enzyme that, acting as main receptor of the nitric oxide (NO), catalyzes the conversion of guanosine-5'-triphosphate (GTP) in 3', 5'-guanosine monophosphate (cGMP) and pyrophosphate. This NO-sGC-cGMP pathway controls vascular smooth-muscle relaxation, vascular tone, and vascular remodeling, and is activated by VEGF signaling. Inhibition of sGC reduces VEGF-induced angiogenesis [88,89]. Moreover, activation of sGC inhibits platelet activation [90].

The protein encoded by the *MYL9* gene is a myosin light chain that regulates muscle contraction by modulating the ATPase activity of myosin heads. In platelets, *MYL9* is associated with *MYH9*, the major nonmuscle myosin expressed in megakaryocytes and platelets. Defects in the *MYH9* gene are responsible of different autosomal dominant disorders characterized by thrombocytopenia and platelet macrocytosis [91,92]. Moreover, it has been demonstrated that *MYL9* is involved in pro-platelet formation [93]. In megakaryocytic cells, *MYL9* expression is regulated by *RUNX1*, a major hematopoietic transcription factor whose haplo-deficiency is associated with familial thrombocytopenia, platelet dysfunction, and predisposition to leukemia [94].

The *ITGA2B* gene encodes the integrin alpha chain 2b, a subunit of the glycoprotein IIb/IIIa, and an integrin complex expressed on the platelet surface. On the activated platelets, it acts as receptor for fibrinogen; this binding induces platelet aggregation, an essential event in thrombus formation, and permits clot retraction. Defects in the *ITGA2B* gene cause Glanzmann thrombasthenia, an autosomal recessive bleeding disorder characterized by failure of platelet aggregation and by absent or diminished clot retraction [95]. Moreover, a GWAS on platelet count revealed a SNP in the *ITGA2B* gene region associated with platelets count (rs708382, P = 1.51x10^-8^) [49]

As for the *ZFPM1* and *JMJD1C* genes, the observed connection between VEGF levels and *GUCY1A3*, *MYL9* and *ITGA2B* genes could be due, therefore, to a regulation of the number and/or the functionality of the circulating platelets. Overall our data suggest that studies clarifying whether the relationship between these genes and VEGF levels is mediated by platelets may be helpful to better understand the role of these genes in VEGF regulation.

In conclusion, the identification of novel genes and pathways associated with circulating VEGF levels could lead to new preventive and therapeutic strategies for a wide variety of diseases in which a pathophysiological role for VEGF has been implicated.

The major strength of this work is that it is the largest GWAS of circulating VEGF to date. A limitation is that, due to the heterogeneity in VEGF levels among the cohorts, a sample size-weighted Z-score method was used to perform the GWAS meta-analysis, which has lower power to detect associations compared to inverse-variance weighted meta-analysis, hence we may have failed to detect some real associations. Further, our analysis focused mostly on common and less frequent variants. Therefore, we could not comprehensively assess the effect of rare variants on VEGF levels. Identifying rare variants in future studies, could contribute to further increasing the proportion of variance in circulating VEGF explained. Also, our study was confined to individuals of European ancestry. The results need to be replicated in other racial and ethnic groups. Finally, a functional validation of the identified associations is needed.

## Materials and Methods

### Subjects

Six discovery data sets including 13,312 samples were analyzed in the Stage 1. The participating discovery studies were the Age Gene/Environment Susceptibility Reykjavik Study (AGES, n = 1,548), the Cilento study (Cilento, n = 1,115), the Framingham Heart Study (FHS, n = 7,048), the Ogliastra Genetic Park (OGP, n = 897), the Prospective Investigation of the Vasculature in Uppsala Seniors Study (PIVUS, n = 945), and the Val Borbera study (VB, n = 1,759). Two additional studies, the Gioi population (Gioi, n = 470) and the Sorbs population (Sorbs, n = 659) provided data for an *in-silico* replication (Stage 2). Further a *de-novo* replication (Stage 3) was undertaken in the STANISLAS Family Study (SFS, n = 676) and in a sample of hypertensive adults (HT, n = 995) from the Biological Resources Center (BRC) IGE-PCV “Interaction Gène-Environment en Physiopathologie Cardio-Vasculaire. The participating cohorts are described further in Section 1 in S1 Text. The local institutional ethics boards for each study approved the study design. Each subject signed an informed consent before participating to the study. Further details can be found in S5 Table.

### Genotyping, imputation and phenotype collection

In the discovery and *in-silico* replication cohorts, genotyping was performed using various arrays, and imputation was carried out using the 1000 genome v3 as reference panel in all studies. Details of pre-imputation quality control parameters, genotyping platforms and imputation parameters for each study are provided in S1 Table. In all cohorts blood samples were collected after an overnight fast, and serum/plasma samples were prepared and stored as described in Section 2 in S1 Text. Serum VEGF levels (plasma VEGF were measured in SFS and HT) were measured using commercial ELISA assays as detailed in Section 2 in S1 Text. The *de-novo* genotyping at SFS and HT was undertaken on a competitive allele specific PCR (KASP) chemistry array and variants were called using a FRET-based genotyping system.

### Genome-wide analyses in contributing studies

In each individual study, a natural log-transformation of VEGF levels was applied. To do that, in a few studies (AGES, OGP, VB, and Sorbs) where some individuals had VEGF levels below the detection threshold of the assay, half the minimum value of VEGF found in that cohort was arbitrarily assigned to each such participant [96]. The transformed trait, adjusted for age, sex and additional study-specific covariates (e.g. principal components associated with VEGF levels, study center for multi-site studies), was related to the variant dosages using a linear regression. Studies with familial correlation used linear mixed effect models to account for familial relatedness. Detailed information about the software used in each cohort is reported in the S1 Table. An additive genetic model with 1 degree of freedom was applied. Study specific results of genome-wide per-variant associations underwent additional quality control prior to meta-analysis. Checking of file formatting, data plausibility, and distributions of test statistics and quality measurements was facilitated by the *gwasqc* function of the GWAtoolbox package v1.0.0 in R [97]. Prior to the meta-analysis, variants with low minor allele frequency (<1%) and poor imputation quality (r^2^< 0.4) were removed.

### Meta-analysis of GWAS

Meta-analysis was performed in METAL using an effective sample size weighted Z-score method [98]. This method was chosen over an inverse-variance meta-analysis because of different covariate-adjusted mean values and standard deviations in VEGF levels among studies. The results of meta-analysis were adjusted for genomic control inflation factor. To define the effective sample size, the product of the sample size and the imputation quality for each variant was calculated in each cohort [99]. The sum of the product of each cohort divided by overall sample size represents the proportion of the effective sample size for each variant Eq (1).

$$\left[\sum_{i=1}^{C}N_{i}\times r_{i}^{2}\right]/13,312=Effective\;sample\;size$$ (1)

where *C* is the total number of participating cohorts, *i* indicates the specific cohort, *N* is the sample size used for the variant association test, and r^*2*^ is imputation quality of the variant. After completing initial quality control checks, 6,705,861 variants, each of which was informative at an effective sample size of >70%, were included in the meta-analysis (Stage 1). The genomic control inflation factor of the metal analysis was 1.003. All variants having a p-value less than 5x10^-8^ were considered to be genome-wide significant.

### Conditional analysis

To identify all independent associations within the loci reaching genome-wide significance, conditional analyses were performed in a forward stepwise fashion, examining the most significant association and including in successive association models the next most significantly associated variant (P<5x10^-8^) in a specific region at each step (referred to as the top variant in Eq (2)). We repeated this process until no more genome-wide significant associations were found. The conditional analysis model follows the formula (2).

$$ln\left(VEGF\right)=\beta_{0}+\beta_{1}variant+\sum_{i=1}^{n}\beta_{i}Covariates_{i}+\sum_{j=1}^{k}\beta_{j}Top\;variant_{j}$$ (2)

where *n* is the number of covariates used in the primary GWAS, *k* is the number of steps. The conditional analysis was only performed in FHS because it represents the largest cohort in the meta-analysis. The final conditional analysis model included 10 independent variants with p-values less than 5x10^-8^ in FHS.

### Replication stages

Genome-wide significant variants identified in the conditional analysis were examined in the two *in-silico* replication cohorts and also carried forward to *de-novo* replication. Furthermore, for each suggestive locus (5x10^-8^<P<1x10^-5^) the lead variant was also examined in the *in-silico* replication sample, and those suggestive variants that reached a genome-wide significant p-value in a meta-analysis of the discovery and *in-silico* replication data (Stage 2) were also carried forward to the *de-novo* replication phase. To check for the presence of other independent variants in the suggestive regions, a clumping procedure implemented in PLINK [100] was performed. The 1000-genome v3 genotypes were used as reference panel for LD calculation; the physical threshold for clumping was 1 Mb, and the r^2^ threshold for clumping was 0.1.

For selected variants that failed *de-novo* genotyping, a proxy variant having either the highest linkage disequilibrium (LD) value, or the variant in the same region with the next lowest p-value was genotyped instead of the lead variant. We considered as replicated, all variants that reached a genome-wide significance level in the meta-analysis of the discovery and the *in-silico* and *de-novo* replication samples (Stage 3).

For the replicated variants, an inverse variance-weighted meta-analysis was also performed as a secondary analysis, including in the analysis all the discovery and replication cohorts.

### Explained variance

The variants identified after replication stages were used to estimate, in each cohort, a genetic score associated with circulating VEGF levels by summing the product of the beta-estimate and genotype for each variant in a given individual Eq (3).

$$RiskScore=\sum_{i=1}^{10}\beta_{i}*Genotype_{i}$$ (3)

where *i* is the variant, *β* is effect size of the variant in the cohort, and genotype is additively coded genotype of the variant. The proportion of phenotypic variance explained by the variants incorporated in the score was estimated fitting two linear mixed effect models, in which VEGF levels were regressed, respectively, on: 1) gender and age (basic model); 2) gender, age, and genetic risk score (risk score model). The variance explained by the replicated variants was estimated as the difference between the variance explained by the risk score model and that explained by the basic model. The *lmekin* function (R package), which uses the genomic kinship matrix to correct for relatedness between individuals, if any, was applied.

### DNA functional element analysis

The replicated SNPs and variants in LD with them (r^2^>0.8) were investigated for the presence of chromatin histone marks and hypersensitive DNAse elements using data from ENCODE included in Haploreg_v3 software (http://www.broadinstitute.org/mammals/haploreg/haploreg_v3.php) [45].

### Gene expression analysis

A database of expression Single Nucleotide Polymorphism (eSNP) was created collecting results from multiple published sources, reported in Section 3 in S1 Text. The eSNP results from each study were included in the database if they met criteria for statistical thresholds for association with gene transcript levels as described in the original references. To search for eQTLs among the associations found in the meta-analysis we queried this database for the replicated variants and their proxies (r^2^>0.8).

### Biological pathway analysis

Two different approaches were used to identify biological pathways influencing VEGF variability.

The GSEA-like statistical test implemented in MAGENTA program was used to test the over-representation of genes containing VEGF-associated variants in a given biological pathway. To do that, all data of meta-analysis results from Stage 1 were used and the gene-set annotations from the Kyoto Encyclopedia of Genes and Genomes (KEGG), PANTHER, INGENUITY, Gene Ontology, REACTOME and BIOCARTA databases were applied. Each gene in the genome was scored by the most significant association p-value among all the SNPs located within a region from 110 kb upstream to 40 kb downstream of each gene’s transcript boundaries. Confounding effects on gene association scores were identified and corrected for. This “normalized best gene score” was used to evaluate the gene enrichment against a null distribution of 10,000 gene sets of identical set size that are randomly sampled from the genome. The 95^th^ percentile of all gene scores for the meta-analysis was used as the enrichment cutoff. Genes within the HLA-region were excluded from analysis due to difficulties in accounting for gene density and LD patterns and only gene sets with at least 10 genes were included in the analysis. Significance was determined when an individual pathway reached a false discovery rate (FDR)<0.05.

The Ingenuity Pathway Analysis software (IPA) was used to explore the functional relationship between genes of interest, selected from candidate regions. For this purpose, a candidate region was defined as comprising all variants between the first and last variants in a chromosomal region that were associated at genome-wide significance with circulating VEGF levels, either in discovery phase (Stage 1) or the combined discovery and replication meta-analysis (Stage 3). The genes of interest were chosen including all within 60kb of each of the candidate regions. A total of 26 genes (listed in the S4 Table) fit this description and served as ‘input’ genes for the pathway analysis. Direct and indirect interactions, a reasonable confidence (experimentally observed, highly predicted, or moderately predicted) and a maximum size of 70 genes/proteins per network were used as parameters in the analysis.

## Supporting Information

S1 Fig Genome-wide association plot based on meta-analysis in the discovery. Sample: X-axis represents the chromosome number and y-axis represents–log_10_ (P) of the variants analyzed in GWAS after applying QC options. Panel A shows all variants. Panel B shows the variants having the p-value > 5x10^-20^. The red line indicates the genome-wide significant level (P = 5x10^-8^) and the blue line indicates the suggestive significant level (P = 1x10^-5^).
(TIFF)

S2 Fig Quantile-Quantile plot using meta-analysis result of discovery set. X-axis is expected–log_10_ (P) and Y-axis is observed–log_10_ (P). Panel A shows all variants. Panel B shows the variants except for variants located at significant regions on chromosome 6, 8 and 9.
(TIFF)

S1 Table Genotyping and quality control. (DOCX)

S2 Table Genome-wide significant variants in discovery sample meta-analysis. (DOCX)

S3 Table *Cis* and *Trans* eQTL results. (DOCX)

S4 Table Focus genes for ingenuity pathway analysis. (DOCX)

S5 Table Ethics statement of participant studies. (XLSX)

S1 Text Supporting information for six novel loci associated with circulating VEGF levels identified by a meta-analysis of genome-wide association studies. (DOCX)
