# Supplementary figures and images for: Six Novel Loci Associated with Circulating VEGF Levels Identified by a Meta-analysis of Genome-Wide Association Studies
Source: PLoS Genet. 2016 Feb 24;12(2):e1005874. doi: 10.1371/journal.pgen.1005874 (PMC4766012; doi:10.1371/journal.pgen.1005874)

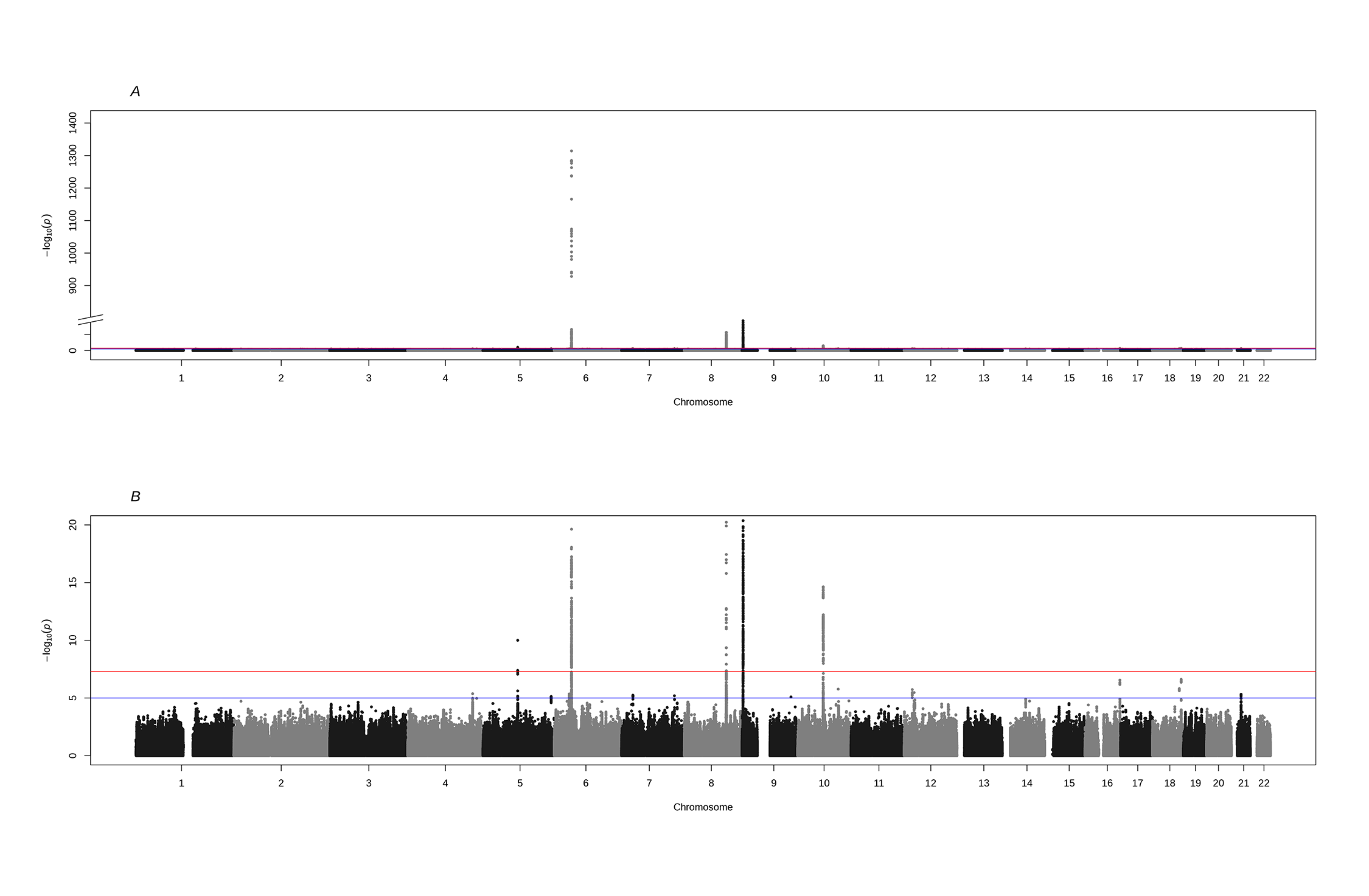

Supplement: S1 Fig — Sample: X-axis represents the chromosome number and y-axis represents–log10 (P) of the variants analyzed in GWAS after applying QC options. Panel A shows all variants. Panel B shows the variants having the p-value > 5x10^-20^. The red line indicates the genome-wide significant level (P = 5x10^-8^) and the blue line indicates the suggestive significant level (P = 1x10^-5^). (TIFF) [file pgen.1005874.s001.tiff]

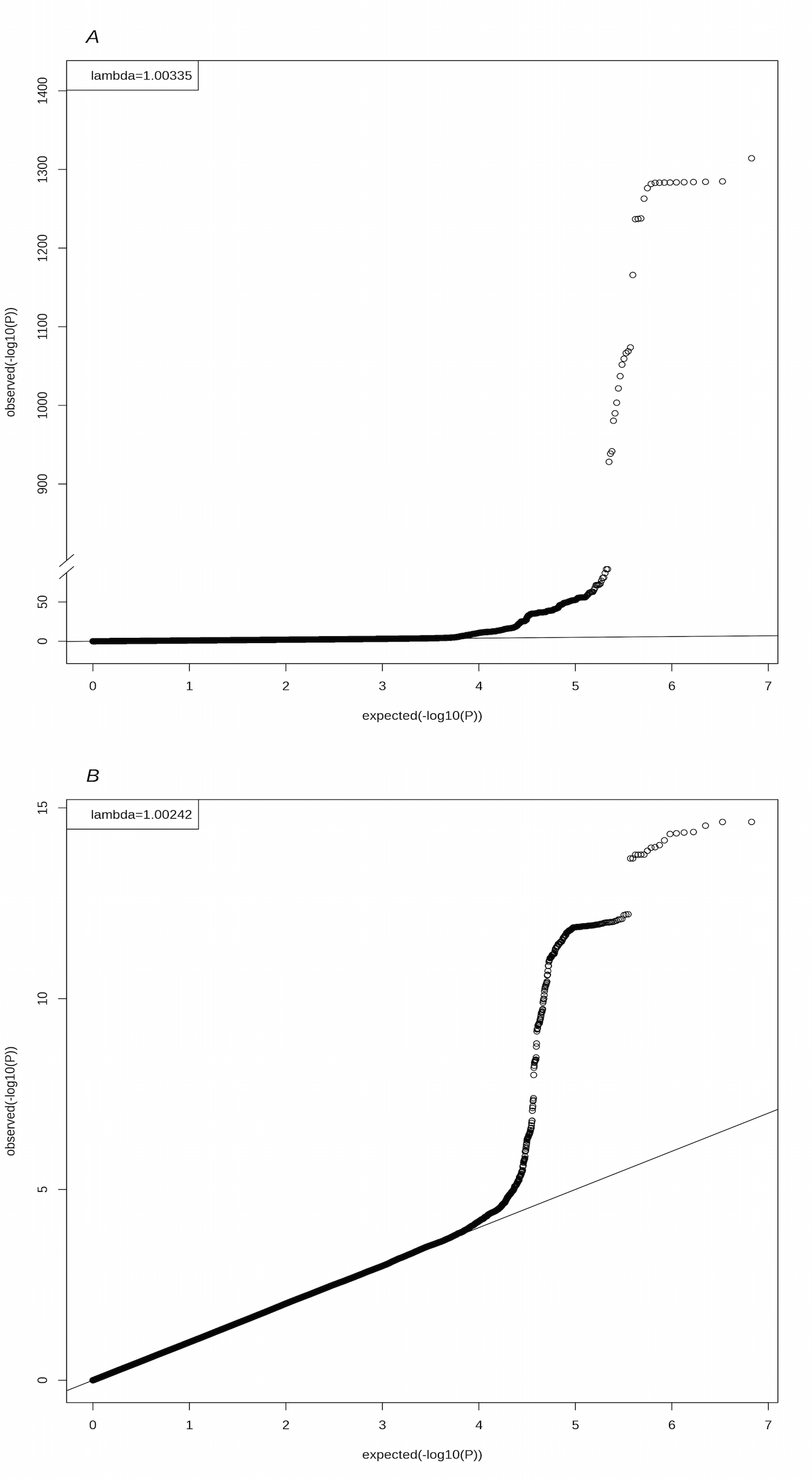

Supplement: S2 Fig — X-axis is expected–log10 (P) and Y-axis is observed–log10 (P). Panel A shows all variants. Panel B shows the variants except for variants located at significant regions on chromosome 6, 8 and 9. (TIFF) [file pgen.1005874.s002.tiff]
