# Supplementary material for: Six Novel Loci Associated with Circulating VEGF Levels Identified by a Meta-analysis of Genome-Wide Association Studies
Source: PLoS Genet. 2016 Feb 24;12(2):e1005874. doi: 10.1371/journal.pgen.1005874 (PMC4766012; doi:10.1371/journal.pgen.1005874)
Supplement: S1 Table — (DOCX) [file pgen.1005874.s003.docx]

**Supplementary Table1.**

| **Study^#^** | **AGES** | **Cilento** | **FHS** | **OGP** | **PIVUS** | **VB** | **Gioi** | **Sorbs** | **HT** | **SFS** |
| --- | --- | --- | --- | --- | --- | --- | --- | --- | --- | --- |
| Array type(s) | Illumina Hu370CNV | Illumina 370 K (859 individuals); Illumina OmniExpress 700K (288 individuals) | Affymetrix GeneChip Human Mapping 500k Array Set and 50k Human Gene Focus Panel | Affymetrix 500K | Illumina OmniExpress | Illumina 370 K (1664 individuals); Illumina OmniExpress 700K (121 individuals) | Illumina OmniExpress 700K | 500K Affymetrix GeneChip (250K Sty and 250K Nsp arrays, Affymetrix, Inc) and Affymetrix Genome-Wide Human SNP Array 6.0 | Competitive allele specific PCR (KASP) chemistry | Competitive allele specific PCR (KASP) chemistry |
| Genotype calling | GenomeStudio | GenomeStudio | affymetrix BRLMM | BRLMM | GenCall | BeadStudio analysis software | GenomeStudio | BRLMM algorithm (Affymetrix, Inc) for 500K and Birdseed Algorithm for Genome-Wide Human SNP Array 6.0 | FRET-based genotyping system | FRET-based genotyping system |
| QC filters for genotyped SNPs used for imputation | Imputation performed with the following filters: Call rate<97%, MAF<1%. Genotype dosages were used for all SNPs. | Imputation was performed in the two groups (859 and 288 individuals) separately, using the following filters: call rate<95%, MAF<1%. For the directly typed SNPs in common between the two groups, the real genotype was used in the association analysis, while the imputation dosage was considered for the other SNPs. | call rate<95%, MAF<1% HWE,Pvalue< 0.000001. | Imputation was performed in 1164 individuals using the following filters: call rate <95%, MAF<5%, HWE pvalue<10-4. | Imputation was performed in 949 individuals using the following filters: call rate <95%, HWE pvalue<10-6, monomorphisms, missing genotype rate >0.01 (if MAF<0.05), missing genotype rate>0.05 (if MAF>=0.05) | Imputation was performed in the two groups (1664 and 121 individuals) separately, using the following filters: call rate >=90%, MAF >=1%, HWEp<= 0.001. | Imputation was performed using the following filters: call rate<95%, MAF<1%. For the directly typed SNPs the real genotype was used in the association analysis, while the imputation dosage was considered for the other SNPs. | MAF ≥ 1%, callrate ≥ 95%, HWE P > 10‐4 | NA | NA |
| No of SNPs used for imputation | 308,340 SNPs | 306995 (for the 859 individuals genotyped with the 370K); 588083 (for the 288 individuals genotyped with the 700K) | 412053 | 362151 | 645318 | 332887 (for 1664 individuals with Illumina 370k chip);648130 (for 121 individuals with Illumina OmniExpress 700K) | 588083 | 378,258 | NA | NA |
| Pre-phasing Software | MACH 1.0.16b | MACH version 1.0.16 | MACH | SHAPEIT2 | IMPUTE2 | SHAPEIT v2.r613 for 1664 individuals with Illumina 370K chip; none for 121 individuals with Illumina OmniExpress 700K | MACH version 1.0.16 | Mach 1.0.16 | NA | NA |
| Imputation Software | minimac release 2012-05-29 | minimac release 2012-05-29 | minimac | IMPUTE2 | IMPUTE2 | IMPUTE version 2.2.2 | minimac release 2012-05-29 | Minimac RELEASE STAMP 2012-03-14 | NA | NA |
| Filtering of imputed genotypes | none | only monomorphic SNPs were excluded from the analysis | monomorphic SNPs were excluded from the analysis | Call rate<95%, MAF<1%, Rsq<0.4 | Call rate<95%, MAF<1%, Rsq<0.4 | none | only monomorphic SNPs were excluded from the analysis | None | NA | NA |
| Data management and statistical analysis | R, ProbABEL | R, GenABEL, ProbABEL (mmscore function was used to account for relatedness) | Pipeline  (*lmekin* function was used to account for relatedness) | R, DatABEL, GenABEL (mmscore function was used to account for relatedness) | STATA, SNPTest | R, GEMMA | R, GenABEL, ProbABEL (mmscore function was used to account for relatedness) | R, GenABEL, ProbABEL (mmscore function was used to account for relatedness) | SPSS, Plink | R packages (FactoMineR, GWAF) |

*Study names were abbreviate as Age Gene/Environment Susceptibility Reykjavik Study (AGES), Cilento study (Cilento), Framingham Heart Study (FHS), Ogliastra Genetic Park (OGP), Prospective Investigation of the Vasculature in Uppsala Seniors Study (PIVUS), Val Borbera (VB), a village included in the Cilento study (Gioi), Sorbs population (Sorbs), hypertensive adults (HT) and STANISLAS Family Study (SFS) from Biological Resources Bank (BRB).
