# Supplementary material for: Six Novel Loci Associated with Circulating VEGF Levels Identified by a Meta-analysis of Genome-Wide Association Studies
Source: PLoS Genet. 2016 Feb 24;12(2):e1005874. doi: 10.1371/journal.pgen.1005874 (PMC4766012; doi:10.1371/journal.pgen.1005874)
Supplement: S2 Table — (DOCX) [file pgen.1005874.s004.docx]

**Supplementary Table2.**

| **Variant rsID** | **Chr** | **Position** | **Coded*** | **CodedAF^†^** | **Zscore** | **P** | **Direction^‡^** |
| --- | --- | --- | --- | --- | --- | --- | --- |
| rs145243487 | 5 | 88035532 | A | 0.9565 | -5.469 | 4.53E-08 | ------ |
| rs34845073 | 5 | 88082933 | D | 0.1147 | 5.488 | 4.07E-08 | ++++++ |
| rs146956999 | 5 | 88089144 | D | 0.1166 | 5.457 | 4.85E-08 | ++++++ |
| rs114694170 | 5 | 88180196 | T | 0.9566 | -6.467 | 1.00E-10 | ------ |
| rs833066 | 6 | 43683817 | T | 0.5878 | -6.165 | 7.05E-10 | ------ |
| rs833054 | 6 | 43688746 | A | 0.6502 | -6.125 | 9.08E-10 | ------ |
| rs2759270 | 6 | 43689395 | A | 0.6589 | -5.803 | 6.51E-09 | ------ |
| rs11965885 | 6 | 43693094 | T | 0.5628 | -8.815 | 1.20E-18 | ------ |
| rs10948085 | 6 | 43693563 | T | 0.2978 | 8.197 | 2.47E-16 | ++++++ |
| rs4711748 | 6 | 43694598 | T | 0.2407 | 6.244 | 4.26E-10 | ++++++ |
| rs9462920 | 6 | 43694610 | A | 0.2818 | 8.171 | 3.06E-16 | ++++++ |
| rs9462921 | 6 | 43694634 | C | 0.2817 | 8.16 | 3.35E-16 | ++++++ |
| rs9472112 | 6 | 43694773 | A | 0.2818 | 8.185 | 2.73E-16 | ++++++ |
| rs4711749 | 6 | 43694823 | A | 0.2398 | 6.26 | 3.85E-10 | ++++++ |
| rs12193478 | 6 | 43695371 | A | 0.2398 | 6.267 | 3.69E-10 | ++++++ |
| rs9472113 | 6 | 43695564 | A | 0.2815 | 8.224 | 1.97E-16 | ++++++ |
| rs9472114 | 6 | 43695675 | A | 0.7185 | -8.164 | 3.23E-16 | ------ |
| rs6917406 | 6 | 43695917 | A | 0.7154 | -8.27 | 1.34E-16 | ------ |
| rs9462922 | 6 | 43697141 | T | 0.2841 | 8.189 | 2.63E-16 | ++++++ |
| rs9472115 | 6 | 43697187 | A | 0.8387 | -6.089 | 1.14E-09 | ------ |
| rs9472116 | 6 | 43697831 | A | 0.7167 | -8.234 | 1.82E-16 | ------ |
| rs6929236 | 6 | 43697912 | T | 0.7135 | -8.216 | 2.11E-16 | ------ |
| rs6909616 | 6 | 43698198 | T | 0.2894 | 7.934 | 2.12E-15 | ++++++ |
| rs6909820 | 6 | 43698323 | T | 0.2534 | 6.81 | 9.79E-12 | ++++++ |
| rs1591301 | 6 | 43698797 | A | 0.747 | -6.778 | 1.22E-11 | ------ |
| rs9472117 | 6 | 43699140 | A | 0.2839 | 8.205 | 2.31E-16 | ++++++ |
| rs12662506 | 6 | 43699242 | A | 0.252 | 6.831 | 8.43E-12 | ++++++ |
| rs1591302 | 6 | 43700010 | A | 0.7155 | -8.189 | 2.62E-16 | ------ |
| rs6899412 | 6 | 43700466 | T | 0.748 | -6.87 | 6.44E-12 | ------ |
| rs13214578 | 6 | 43701873 | T | 0.1899 | 6.98 | 2.96E-12 | ++++++ |
| rs12193423 | 6 | 43702246 | T | 0.8101 | -6.993 | 2.69E-12 | ------ |
| rs34528081 | 6 | 43704417 | I | 0.5992 | -8.547 | 1.27E-17 | ------ |
| rs11969760 | 6 | 43705601 | T | 0.2292 | 6.354 | 2.09E-10 | ++++++ |
| rs11967174 | 6 | 43705624 | T | 0.7705 | -6.355 | 2.08E-10 | ------ |
| rs945128 | 6 | 43710756 | A | 0.8208 | -7.046 | 1.84E-12 | ------ |
| rs945129 | 6 | 43710959 | C | 0.8224 | -6.958 | 3.46E-12 | ------ |
| rs7741844 | 6 | 43711196 | A | 0.1821 | 7.186 | 6.69E-13 | ++++++ |
| rs4714695 | 6 | 43711459 | A | 0.1791 | 6.99 | 2.76E-12 | ++++++ |
| rs1535506 | 6 | 43711847 | T | 0.1791 | 6.989 | 2.77E-12 | ++++++ |
| rs7747520 | 6 | 43712351 | A | 0.1795 | 7 | 2.56E-12 | ++++++ |
| rs2010963 | 6 | 43738350 | C | 0.3465 | 5.592 | 2.25E-08 | ++++++ |
| rs58414032 | 6 | 43739523 | C | 0.3399 | 5.773 | 7.79E-09 | ++++++ |
| rs865577 | 6 | 43742419 | C | 0.3574 | 6.039 | 1.55E-09 | ++++++ |
| rs833068 | 6 | 43742527 | A | 0.3544 | 5.977 | 2.27E-09 | ++++++ |
| rs833069 | 6 | 43742579 | T | 0.6337 | -6.308 | 2.83E-10 | ------ |
| rs3024991 | 6 | 43743060 | I | 0.362 | 5.981 | 2.22E-09 | ++++++ |
| rs3024997 | 6 | 43745107 | A | 0.3441 | 5.886 | 3.96E-09 | ++++++ |
| rs3024998 | 6 | 43745577 | T | 0.3428 | 5.942 | 2.82E-09 | ++++++ |
| rs9472153 | 6 | 43882612 | A | 0.4145 | 5.834 | 5.40E-09 | +++++- |
| rs67798973 | 6 | 43882777 | A | 0.4358 | 6.531 | 6.52E-11 | +++++- |
| rs7747448 | 6 | 43882924 | A | 0.2863 | -10.628 | 2.21E-26 | ------ |
| rs4276495 | 6 | 43883259 | A | 0.6994 | 12.116 | 8.64E-34 | ++++++ |
| rs11752787 | 6 | 43884197 | A | 0.0335 | -6.443 | 1.17E-10 | -+-?-- |
| rs4711755 | 6 | 43884229 | C | 0.3141 | -15.047 | 3.60E-51 | ------ |
| rs4714714 | 6 | 43884812 | A | 0.9665 | 6.489 | 8.65E-11 | +-+?++ |
| rs4711756 | 6 | 43885007 | T | 0.0705 | -6.857 | 7.05E-12 | ------ |
| rs4714715 | 6 | 43885087 | T | 0.9666 | 6.543 | 6.02E-11 | +-+?++ |
| rs9462938 | 6 | 43887745 | T | 0.2266 | -13.027 | 8.59E-39 | ------ |
| rs113614479 | 6 | 43888107 | A | 0.9633 | 7.014 | 2.32E-12 | +++?++ |
| rs146595059 | 6 | 43888246 | A | 0.9629 | 7.027 | 2.12E-12 | +++?++ |
| rs4714716 | 6 | 43888501 | A | 0.9682 | 6.412 | 1.43E-10 | +++?++ |
| rs62401227 | 6 | 43890619 | T | 0.2153 | -14.566 | 4.63E-48 | ------ |
| rs1776721 | 6 | 43890983 | T | 0.2911 | -15.255 | 1.53E-52 | ------ |
| rs7356919 | 6 | 43891365 | A | 0.2081 | -14.731 | 4.10E-49 | ------ |
| rs62401228 | 6 | 43891653 | T | 0.8001 | 15.133 | 9.77E-52 | ++++++ |
| rs1750570 | 6 | 43892235 | T | 0.2078 | -14.8 | 1.47E-49 | ------ |
| rs6921575 | 6 | 43892269 | T | 0.2053 | -14.832 | 9.07E-50 | ------ |
| rs3888006 | 6 | 43892938 | A | 0.2078 | -14.794 | 1.60E-49 | ------ |
| rs716607 | 6 | 43893228 | T | 0.6788 | 13.158 | 1.53E-39 | ++++++ |
| rs7745517 | 6 | 43895095 | T | 0.2068 | -15.068 | 2.64E-51 | ------ |
| rs62401230 | 6 | 43896312 | A | 0.2045 | -15.084 | 2.07E-51 | ------ |
| rs9472155 | 6 | 43897727 | T | 0.2083 | -14.923 | 2.33E-50 | ------ |
| rs62401231 | 6 | 43898150 | A | 0.6821 | 13.16 | 1.50E-39 | ++++++ |
| rs62401232 | 6 | 43898345 | A | 0.2085 | -14.918 | 2.52E-50 | ------ |
| rs833623 | 6 | 43898777 | T | 0.5732 | -13.59 | 4.62E-42 | ------ |
| rs74633169 | 6 | 43899221 | D | 0.206 | -15.265 | 1.31E-52 | ------ |
| rs7767550 | 6 | 43899252 | A | 0.2065 | -15.193 | 3.94E-52 | ------ |
| rs62401233 | 6 | 43899316 | C | 0.2065 | -15.217 | 2.74E-52 | ------ |
| rs864430 | 6 | 43899528 | T | 0.5731 | -13.622 | 2.95E-42 | ------ |
| rs9462939 | 6 | 43899646 | T | 0.2064 | -15.309 | 6.66E-53 | ------ |
| rs9462940 | 6 | 43900048 | T | 0.2061 | -15.332 | 4.65E-53 | ------ |
| rs62401235 | 6 | 43900192 | T | 0.2061 | -15.338 | 4.23E-53 | ------ |
| rs833622 | 6 | 43900404 | T | 0.4248 | 13.493 | 1.71E-41 | ++++++ |
| rs844294 | 6 | 43900707 | T | 0.5066 | 11.63 | 2.91E-31 | ++++++ |
| rs1326141 | 6 | 43903277 | A | 0.2038 | -15.318 | 5.83E-53 | ------ |
| rs1886979 | 6 | 43904901 | A | 0.5694 | -13.786 | 3.10E-43 | ------ |
| rs9381267 | 6 | 43905506 | C | 0.5866 | -13.152 | 1.67E-39 | ------ |
| rs9472158 | 6 | 43918897 | A | 0.5436 | -65.676 | 2.86E-939 | ------ |
| rs6916314 | 6 | 43919162 | A | 0.5441 | -65.311 | 6.97E-929 | ------ |
| rs6916540 | 6 | 43919416 | T | 0.5318 | -65.782 | 2.62E-942 | ------ |
| rs9472159 | 6 | 43919695 | A | 0.4479 | -73.209 | 1.67E-1166 | ------ |
| rs943075 | 6 | 43922205 | A | 0.4405 | 69.044 | 8.01E-1038 | ++++++ |
| rs78355601 | 6 | 43924763 | A | 0.4495 | -76.804 | 1.21E-1283 | ------ |
| rs4349808 | 6 | 43924774 | T | 0.5553 | 76.763 | 2.80E-1282 | ++++++ |
| rs4349809 | 6 | 43924830 | T | 0.5496 | 76.86 | 1.66E-1285 | ++++++ |
| rs4637627 | 6 | 43925327 | A | 0.4454 | -76.835 | 1.13E-1284 | ------ |
| rs4382251 | 6 | 43925373 | T | 0.4494 | 70.092 | 1.75E-1069 | ++++++ |
| rs4513773 | 6 | 43925526 | A | 0.5547 | 76.814 | 5.71E-1284 | ++++++ |
| rs6921438 | 6 | 43925607 | A | 0.4587 | -77.736 | 6.64E-1315 | ------ |
| rs13206436 | 6 | 43925778 | A | 0.4456 | -76.845 | 5.32E-1285 | ------ |
| rs12205248 | 6 | 43926219 | T | 0.555 | 76.83 | 1.72E-1284 | ++++++ |
| rs7745183 | 6 | 43926635 | T | 0.4592 | 70.016 | 3.44E-1067 | ++++++ |
| rs7745184 | 6 | 43926638 | T | 0.4465 | 70.252 | 2.24E-1074 | ++++++ |
| rs7763440 | 6 | 43926708 | A | 0.4452 | -76.824 | 2.76E-1284 | ------ |
| rs7767396 | 6 | 43927050 | A | 0.5504 | 76.816 | 4.85E-1284 | ++++++ |
| rs4481426 | 6 | 43928108 | T | 0.5496 | -69.78 | 5.25E-1060 | ------ |
| rs4320361 | 6 | 43928511 | T | 0.4499 | -76.81 | 7.98E-1284 | ------ |
| rs9472168 | 6 | 43928985 | A | 0.5615 | 77.603 | 6.10E-1277 | ++++++ |
| rs9472170 | 6 | 43929421 | C | 0.5487 | -69.532 | 1.68E-1052 | ------ |
| rs9472171 | 6 | 43930962 | A | 0.4664 | 67.91 | 4.43E-1004 | ++++++ |
| rs9472172 | 6 | 43930985 | T | 0.4431 | -76.2 | 1.48E-1263 | ------ |
| rs9462949 | 6 | 43931347 | A | 0.5486 | -67.452 | 1.26E-990 | ------ |
| rs11757903 | 6 | 43932223 | A | 0.439 | -75.408 | 1.76E-1237 | ------ |
| rs11757868 | 6 | 43932233 | T | 0.4378 | -75.438 | 1.83E-1238 | ------ |
| rs11757888 | 6 | 43932319 | T | 0.4385 | -75.418 | 8.22E-1238 | ------ |
| rs4714719 | 6 | 43932612 | T | 0.5127 | -68.524 | 2.69E-1022 | ------ |
| rs13206012 | 6 | 43932763 | A | 0.3617 | -67.128 | 3.81E-981 | ------ |
| rs9472179 | 6 | 43938931 | A | 0.5118 | -8.516 | 1.65E-17 | ----+- |
| rs4714722 | 6 | 43939179 | T | 0.514 | -8.498 | 1.93E-17 | ----+- |
| rs3929927 | 6 | 43939204 | A | 0.5141 | -8.494 | 2.00E-17 | ----+- |
| rs3929926 | 6 | 43939313 | A | 0.5141 | -8.494 | 2.00E-17 | ----+- |
| rs3929925 | 6 | 43939373 | A | 0.5124 | -8.476 | 2.33E-17 | ----+- |
| rs12214617 | 6 | 43939923 | T | 0.4771 | 8.431 | 3.42E-17 | ++++-+ |
| rs12214523 | 6 | 43939958 | T | 0.4836 | 8.578 | 9.62E-18 | ++++-+ |
| rs6936047 | 6 | 43939990 | A | 0.5164 | -8.578 | 9.62E-18 | ----+- |
| rs9462951 | 6 | 43940150 | T | 0.4846 | 8.422 | 3.69E-17 | ++++-+ |
| rs9472183 | 6 | 43940202 | A | 0.5123 | -8.343 | 7.24E-17 | ----+- |
| rs9472184 | 6 | 43940269 | A | 0.517 | -8.364 | 6.04E-17 | ----+- |
| rs201930756 | 6 | 43940368 | I | 0.4732 | 8.85 | 8.77E-19 | ++++-+ |
| rs34467391 | 6 | 43940371 | I | 0.4867 | 8.354 | 6.62E-17 | ++++-+ |
| rs60200995 | 6 | 43940373 | I | 0.4735 | 7.934 | 2.13E-15 | ++++-+ |
| rs34745324 | 6 | 43941098 | A | 0.5156 | -8.474 | 2.37E-17 | ----+- |
| rs4714723 | 6 | 43941349 | A | 0.5153 | -8.42 | 3.77E-17 | ----+- |
| rs4714724 | 6 | 43941645 | T | 0.4869 | 8.297 | 1.07E-16 | ++++-+ |
| rs4714725 | 6 | 43941682 | T | 0.5122 | -8.438 | 3.23E-17 | ----+- |
| rs4714726 | 6 | 43941782 | A | 0.5135 | -8.381 | 5.26E-17 | ----+- |
| rs10807292 | 6 | 43941986 | T | 0.4866 | 8.375 | 5.55E-17 | ++++-+ |
| rs10807293 | 6 | 43942012 | A | 0.5134 | -8.373 | 5.62E-17 | ----+- |
| rs4714727 | 6 | 43942224 | T | 0.5136 | -8.363 | 6.10E-17 | ----+- |
| rs4714728 | 6 | 43942245 | A | 0.5143 | -8.353 | 6.64E-17 | ----+- |
| rs4714729 | 6 | 43942389 | T | 0.5106 | -8.276 | 1.28E-16 | ----+- |
| rs4714731 | 6 | 43942566 | T | 0.5135 | -8.373 | 5.62E-17 | ----+- |
| rs34056938 | 6 | 43944421 | I | 0.5489 | -5.862 | 4.58E-09 | ---++- |
| rs1740071 | 6 | 43944859 | A | 0.5498 | -5.914 | 3.33E-09 | ---++- |
| rs1740073 | 6 | 43947398 | T | 0.3659 | 8.05 | 8.29E-16 | +++--+ |
| rs1740075 | 6 | 43947844 | C | 0.505 | 10.511 | 7.66E-26 | +++-++ |
| rs9369435 | 6 | 43948043 | A | 0.6194 | 9.564 | 1.14E-21 | ++++++ |
| rs2051074 | 6 | 43948453 | A | 0.94 | 7.137 | 9.57E-13 | ++++++ |
| rs12524926 | 6 | 43948873 | T | 0.1991 | -17.137 | 7.82E-66 | ------ |
| rs72867220 | 6 | 43949596 | A | 0.0703 | -10.081 | 6.71E-24 | ------ |
| rs1776703 | 6 | 43949757 | T | 0.5145 | 9.913 | 3.66E-23 | +++-++ |
| rs1740077 | 6 | 43949907 | T | 0.4391 | -12.791 | 1.85E-37 | ------ |
| rs1776704 | 6 | 43949941 | A | 0.4383 | -12.864 | 7.17E-38 | ------ |
| rs1776705 | 6 | 43949958 | T | 0.5612 | 12.848 | 8.84E-38 | ++++++ |
| rs1776706 | 6 | 43949987 | T | 0.4391 | -12.79 | 1.88E-37 | ------ |
| rs4320369 | 6 | 43950062 | T | 0.4373 | -12.606 | 1.97E-36 | ------ |
| rs67470483 | 6 | 43950168 | T | 0.3694 | 7.42 | 1.17E-13 | +++--+ |
| rs4523085 | 6 | 43950169 | A | 0.4381 | -12.874 | 6.28E-38 | ------ |
| rs4434467 | 6 | 43950250 | T | 0.5595 | 12.787 | 1.95E-37 | ++++++ |
| rs4416670 | 6 | 43950453 | T | 0.5592 | 12.766 | 2.53E-37 | ++++++ |
| rs7452472 | 6 | 43950501 | A | 0.561 | 12.798 | 1.67E-37 | ++++++ |
| rs910614 | 6 | 43950618 | T | 0.4392 | -12.789 | 1.90E-37 | ------ |
| rs910613 | 6 | 43950636 | T | 0.5611 | 12.799 | 1.65E-37 | ++++++ |
| rs910612 | 6 | 43950706 | T | 0.4394 | -12.784 | 2.02E-37 | ------ |
| rs910611 | 6 | 43950851 | T | 0.9227 | 10.231 | 1.45E-24 | ++++++ |
| rs1740079 | 6 | 43951239 | A | 0.4376 | -12.801 | 1.63E-37 | ------ |
| rs1776718 | 6 | 43951275 | A | 0.2155 | -15.758 | 6.06E-56 | ------ |
| rs1776717 | 6 | 43951336 | A | 0.2146 | -15.83 | 1.94E-56 | ------ |
| rs9349265 | 6 | 43951385 | C | 0.7854 | 15.825 | 2.09E-56 | ++++++ |
| rs910610 | 6 | 43951440 | A | 0.2146 | -15.821 | 2.22E-56 | ------ |
| rs910609 | 6 | 43951656 | A | 0.2117 | -15.967 | 2.15E-57 | ------ |
| rs910608 | 6 | 43951826 | C | 0.2162 | -15.788 | 3.78E-56 | ------ |
| rs1631155 | 6 | 43951967 | A | 0.7775 | 16.493 | 4.09E-61 | ++++++ |
| rs1631938 | 6 | 43952046 | A | 0.2219 | -16.159 | 9.75E-59 | ------ |
| rs1632037 | 6 | 43952089 | T | 0.2202 | -16.284 | 1.27E-59 | ------ |
| rs55687220 | 6 | 43952263 | A | 0.0771 | -10.285 | 8.22E-25 | ------ |
| rs1740080 | 6 | 43952412 | A | 0.1231 | -10.025 | 1.18E-23 | ------ |
| rs910606 | 6 | 43953329 | A | 0.1199 | -10.098 | 5.64E-24 | ------ |
| rs1631662 | 6 | 43953358 | T | 0.12 | -10.157 | 3.07E-24 | ------ |
| rs910604 | 6 | 43953671 | A | 0.2035 | -14.948 | 1.60E-50 | ------ |
| rs79450569 | 6 | 43954447 | I | 0.0927 | -9.245 | 2.34E-20 | ------ |
| rs72867251 | 6 | 43954809 | A | 0.9292 | 10.55 | 5.10E-26 | ++++++ |
| rs9367181 | 6 | 43956615 | A | 0.8022 | 15.531 | 2.16E-54 | ++++++ |
| rs9462952 | 6 | 43964440 | A | 0.814 | -5.859 | 4.64E-09 | ------ |
| rs3734693 | 6 | 43965165 | T | 0.8138 | -5.833 | 5.45E-09 | ------ |
| rs60805454 | 6 | 43966844 | T | 0.1846 | 5.771 | 7.90E-09 | ++++++ |
| rs7765648 | 6 | 43969257 | C | 0.886 | -6.65 | 2.93E-11 | -----+ |
| rs57055115 | 6 | 43969302 | C | 0.1805 | 5.625 | 1.86E-08 | ++++++ |
| rs7764334 | 6 | 43972571 | T | 0.8179 | -5.762 | 8.33E-09 | ------ |
| rs2273310 | 6 | 43973082 | T | 0.8173 | -5.746 | 9.15E-09 | ------ |
| rs55795851 | 6 | 43973150 | C | 0.8526 | -6.629 | 3.37E-11 | ------ |
| rs6923422 | 6 | 43973863 | T | 0.1146 | 6.868 | 6.52E-12 | ++++++ |
| rs6927996 | 6 | 43974106 | A | 0.8166 | -5.755 | 8.69E-09 | ------ |
| rs6928047 | 6 | 43974166 | A | 0.8167 | -5.743 | 9.29E-09 | ------ |
| rs9381273 | 6 | 43976268 | A | 0.1765 | 5.806 | 6.38E-09 | ++++++ |
| rs11966317 | 6 | 43977172 | A | 0.8391 | -5.961 | 2.51E-09 | ------ |
| rs202223097 | 6 | 43977769 | I | 0.1307 | 6.142 | 8.16E-10 | ++++++ |
| rs6909859 | 6 | 43978323 | A | 0.8125 | -7.167 | 7.69E-13 | ------ |
| rs61372940 | 6 | 43978907 | C | 0.1654 | 6.185 | 6.21E-10 | ++++++ |
| rs926961 | 6 | 43979214 | A | 0.8331 | -6.271 | 3.60E-10 | ------ |
| rs9367182 | 6 | 43979345 | T | 0.8992 | -6.31 | 2.80E-10 | ------ |
| rs9357428 | 6 | 43979409 | C | 0.8992 | -6.306 | 2.87E-10 | ------ |
| rs28475247 | 6 | 43985545 | T | 0.9327 | -5.663 | 1.49E-08 | ------ |
| rs7757763 | 6 | 43985842 | A | 0.1895 | 7.244 | 4.34E-13 | ++++++ |
| rs77630893 | 6 | 43986555 | T | 0.8105 | -7.24 | 4.49E-13 | ------ |
| rs7768018 | 6 | 43987576 | A | 0.8107 | -7.219 | 5.25E-13 | ------ |
| rs11337688 | 6 | 43987787 | D | 0.1888 | 7.256 | 4.00E-13 | ++++++ |
| rs6907643 | 6 | 43990199 | T | 0.2029 | 6.097 | 1.08E-09 | ++++-+ |
| rs6458367 | 6 | 43990498 | A | 0.1777 | 6.105 | 1.03E-09 | ++++-+ |
| rs7757024 | 6 | 43992587 | T | 0.122 | 6.09 | 1.13E-09 | ++++-+ |
| rs9394979 | 6 | 43995208 | A | 0.1483 | -8.381 | 5.24E-17 | ------ |
| rs9394980 | 6 | 43995314 | T | 0.8518 | 8.379 | 5.32E-17 | ++++++ |
| rs9367183 | 6 | 43997973 | A | 0.1479 | -8.337 | 7.61E-17 | ------ |
| rs1008848 | 6 | 43999301 | A | 0.1518 | -7.895 | 2.89E-15 | -+---- |
| rs9381279 | 6 | 43999697 | A | 0.8526 | 8.264 | 1.41E-16 | ++++++ |
| rs4714732 | 6 | 44000031 | A | 0.1518 | -7.913 | 2.51E-15 | -+---- |
| rs78977597 | 6 | 44001029 | T | 0.1439 | -7.988 | 1.37E-15 | ------ |
| rs1922985 | 6 | 44002077 | T | 0.1504 | -8.634 | 5.95E-18 | ------ |
| rs9369440 | 6 | 44002373 | T | 0.3038 | -6.124 | 9.14E-10 | -+---- |
| rs62403174 | 6 | 44002465 | A | 0.1504 | -8.637 | 5.77E-18 | ------ |
| rs57833688 | 6 | 44004867 | A | 0.8112 | 7.638 | 2.20E-14 | ++++++ |
| rs910603 | 6 | 44007446 | A | 0.664 | 5.592 | 2.24E-08 | +-++++ |
| rs910602 | 6 | 44007791 | A | 0.6636 | 5.671 | 1.42E-08 | +-++++ |
| rs9369443 | 6 | 44007895 | A | 0.1951 | -7.475 | 7.72E-14 | ------ |
| rs9381282 | 6 | 44008325 | A | 0.1944 | -7.521 | 5.44E-14 | ------ |
| rs9394981 | 6 | 44008747 | A | 0.6495 | 5.673 | 1.40E-08 | +-++++ |
| rs9381283 | 6 | 44008776 | A | 0.2648 | -5.743 | 9.33E-09 | -+-+-- |
| rs4711761 | 6 | 44009019 | A | 0.2851 | -5.668 | 1.45E-08 | -+-+-- |
| rs200012462 | 6 | 44009819 | I | 0.359 | -6.065 | 1.32E-09 | -+---- |
| rs9349269 | 6 | 44009910 | C | 0.6912 | 5.69 | 1.27E-08 | +-+-++ |
| rs9381284 | 6 | 44010110 | A | 0.79 | 7.044 | 1.87E-12 | +-++++ |
| rs111469860 | 6 | 44010121 | D | 0.211 | -6.779 | 1.21E-11 | -+---- |
| rs4714734 | 6 | 44013954 | C | 0.6389 | 5.863 | 4.55E-09 | +-++++ |
| rs12164037 | 6 | 44014897 | A | 0.289 | -6.701 | 2.07E-11 | -+---- |
| rs62403185 | 6 | 44015092 | T | 0.3348 | -6.201 | 5.62E-10 | -+---- |
| rs9369444 | 6 | 44015894 | T | 0.2862 | -6.862 | 6.78E-12 | -+---- |
| rs9369445 | 6 | 44015916 | A | 0.3327 | -6.348 | 2.18E-10 | -+---- |
| rs2077404 | 6 | 44016153 | T | 0.6347 | 5.717 | 1.09E-08 | +-++++ |
| rs753675 | 6 | 44016989 | T | 0.3501 | -6.221 | 4.94E-10 | -+---- |
| rs1884377 | 6 | 44018156 | T | 0.7991 | 6.956 | 3.49E-12 | ++++++ |
| rs9394982 | 6 | 44019203 | C | 0.2506 | -6.961 | 3.37E-12 | ------ |
| rs6937694 | 6 | 44019518 | T | 0.7002 | 6.733 | 1.66E-11 | +-++++ |
| rs9369446 | 6 | 44019730 | T | 0.7105 | 6.704 | 2.02E-11 | +-++++ |
| rs9472202 | 6 | 44021286 | T | 0.2309 | -6.278 | 3.42E-10 | -+---- |
| rs11375670 | 6 | 44034402 | I | 0.3396 | 5.671 | 1.42E-08 | ++++++ |
| rs5875865 | 6 | 44043449 | D | 0.57 | 8.415 | 3.93E-17 | ++++++ |
| rs10223586 | 6 | 44046413 | A | 0.4338 | -8.364 | 6.04E-17 | ------ |
| rs881934 | 6 | 44050269 | T | 0.5144 | -6.634 | 3.27E-11 | ------ |
| rs6458368 | 6 | 44051221 | T | 0.4615 | -7.564 | 3.92E-14 | ------ |
| rs6935537 | 6 | 44054459 | T | 0.7808 | -6.286 | 3.27E-10 | ------ |
| rs9394983 | 6 | 44054553 | T | 0.4606 | 9.639 | 5.49E-22 | ++++++ |
| rs7755223 | 6 | 44055333 | A | 0.7826 | -6.213 | 5.18E-10 | ------ |
| rs1138991 | 6 | 44058579 | T | 0.3999 | 6.039 | 1.55E-09 | ++++++ |
| rs55934950 | 6 | 44061977 | T | 0.2538 | 5.601 | 2.13E-08 | -+++++ |
| rs2396165 | 6 | 44063217 | T | 0.4954 | -7.479 | 7.49E-14 | -----+ |
| rs4714739 | 6 | 44064064 | A | 0.4845 | -7.526 | 5.25E-14 | -----+ |
| rs72011297 | 6 | 44064851 | D | 0.392 | 5.978 | 2.26E-09 | +++++- |
| rs7742030 | 6 | 44065062 | T | 0.2172 | 5.722 | 1.06E-08 | ++++++ |
| rs7742824 | 6 | 44065311 | A | 0.2172 | 5.713 | 1.11E-08 | ++++++ |
| rs4714741 | 6 | 44065879 | T | 0.516 | 7.374 | 1.65E-13 | +++++- |
| rs57139546 | 6 | 44067745 | T | 0.7828 | -5.708 | 1.15E-08 | ------ |
| rs9381289 | 6 | 44070133 | C | 0.5158 | 7.363 | 1.79E-13 | +++++- |
| rs4610558 | 6 | 44070949 | T | 0.2155 | 5.675 | 1.39E-08 | ++++++ |
| rs4370360 | 6 | 44070960 | C | 0.5146 | 7.347 | 2.03E-13 | +++++- |
| rs9381290 | 6 | 44072405 | A | 0.4841 | -7.362 | 1.82E-13 | -----+ |
| rs57576544 | 6 | 44072726 | T | 0.7829 | -5.688 | 1.29E-08 | ------ |
| rs4714742 | 6 | 44072970 | A | 0.4842 | -7.355 | 1.90E-13 | -----+ |
| rs9369452 | 6 | 44073159 | T | 0.4841 | -7.36 | 1.84E-13 | -----+ |
| rs910616 | 6 | 44074976 | T | 0.4834 | -7.343 | 2.09E-13 | -----+ |
| rs60207481 | 6 | 44075719 | A | 0.1654 | 6.18 | 6.40E-10 | ++++++ |
| rs877111 | 6 | 44076061 | A | 0.5162 | 7.372 | 1.68E-13 | +++++- |
| rs7756283 | 6 | 44076780 | T | 0.4839 | -7.353 | 1.93E-13 | -----+ |
| rs1321097 | 6 | 44077356 | A | 0.4841 | -7.348 | 2.02E-13 | -----+ |
| rs4714745 | 6 | 44077669 | T | 0.4842 | -7.339 | 2.15E-13 | -----+ |
| rs4714747 | 6 | 44079928 | A | 0.5159 | 7.325 | 2.38E-13 | +++++- |
| rs9357430 | 6 | 44080385 | T | 0.5159 | 7.324 | 2.40E-13 | +++++- |
| rs60105057 | 6 | 44080719 | C | 0.7821 | -5.698 | 1.21E-08 | ------ |
| rs9394985 | 6 | 44081006 | C | 0.516 | 7.32 | 2.49E-13 | +++++- |
| rs9394986 | 6 | 44081203 | T | 0.484 | -7.323 | 2.42E-13 | -----+ |
| rs6883 | 6 | 44081718 | A | 0.5146 | 7.344 | 2.08E-13 | +++++- |
| rs4711767 | 6 | 44081987 | T | 0.4802 | -7.059 | 1.67E-12 | -----+ |
| rs7770652 | 6 | 44084684 | T | 0.4952 | -7.558 | 4.10E-14 | -----+ |
| rs73429729 | 6 | 44086727 | A | 0.2174 | 5.698 | 1.21E-08 | ++++++ |
| rs2144510 | 6 | 44087583 | A | 0.4836 | -7.366 | 1.76E-13 | -----+ |
| rs2396167 | 6 | 44088543 | T | 0.4841 | -7.33 | 2.30E-13 | -----+ |
| rs1569889 | 6 | 44089135 | C | 0.484 | -7.338 | 2.17E-13 | -----+ |
| rs4714748 | 6 | 44090168 | A | 0.4839 | -7.345 | 2.05E-13 | -----+ |
| rs10625755 | 6 | 44090458 | I | 0.5113 | 7.369 | 1.72E-13 | +++++- |
| rs1891440 | 6 | 44091165 | T | 0.5161 | 7.351 | 1.97E-13 | +++++- |
| rs713049 | 6 | 44091844 | T | 0.4839 | -7.357 | 1.89E-13 | -----+ |
| rs6918252 | 6 | 44092537 | T | 0.5161 | 7.362 | 1.81E-13 | +++++- |
| rs1935611 | 6 | 44093742 | T | 0.516 | 7.31 | 2.67E-13 | +++++- |
| rs7752653 | 6 | 44093808 | T | 0.7826 | -5.75 | 8.92E-09 | ------ |
| rs2096199 | 6 | 44096014 | A | 0.2238 | 5.613 | 1.99E-08 | ++++++ |
| rs7773700 | 6 | 44097472 | T | 0.7825 | -5.691 | 1.27E-08 | ------ |
| rs12212365 | 6 | 44099561 | A | 0.5164 | 7.334 | 2.23E-13 | +++++- |
| rs9462964 | 6 | 44101276 | T | 0.483 | -7.415 | 1.22E-13 | -----+ |
| rs73429745 | 6 | 44101422 | T | 0.2193 | 5.786 | 7.21E-09 | ++++++ |
| rs66629131 | 6 | 44101468 | C | 0.7815 | -5.767 | 8.08E-09 | ------ |
| rs4714751 | 6 | 44101544 | T | 0.5059 | 7.541 | 4.66E-14 | +++++- |
| rs3841315 | 6 | 44102572 | I | 0.5147 | 7.232 | 4.76E-13 | +++++- |
| rs56168551 | 6 | 44104306 | A | 0.2186 | 5.707 | 1.15E-08 | ++++++ |
| rs7756198 | 6 | 44104395 | T | 0.643 | -6.302 | 2.94E-10 | ------ |
| rs9349273 | 6 | 44104573 | C | 0.6429 | -6.298 | 3.03E-10 | ------ |
| rs9349274 | 6 | 44104617 | T | 0.3541 | 6.169 | 6.88E-10 | ++++++ |
| rs4714753 | 6 | 44104881 | A | 0.643 | -6.304 | 2.90E-10 | ------ |
| rs6925508 | 6 | 44105442 | A | 0.3211 | 7.053 | 1.76E-12 | ++++++ |
| rs9381291 | 6 | 44105715 | T | 0.357 | 6.281 | 3.37E-10 | ++++++ |
| rs59842359 | 6 | 44105929 | T | 0.2232 | 5.803 | 6.51E-09 | ++++++ |
| rs4714755 | 6 | 44105978 | A | 0.4834 | -7.277 | 3.41E-13 | -----+ |
| rs4714756 | 6 | 44105981 | A | 0.5166 | 7.28 | 3.34E-13 | +++++- |
| rs10456528 | 6 | 44110181 | T | 0.2126 | -6.596 | 4.24E-11 | ------ |
| rs10428725 | 6 | 44112409 | T | 0.2093 | -6.523 | 6.88E-11 | -----+ |
| rs151199577 | 6 | 44113516 | D | 0.2084 | -6.543 | 6.04E-11 | -----+ |
| rs35937558 | 6 | 44116785 | I | 0.2114 | -6.287 | 3.24E-10 | -----+ |
| rs11962945 | 6 | 44125185 | A | 0.1943 | -6.858 | 7.00E-12 | ------ |
| rs12210167 | 6 | 44125996 | A | 0.8047 | 6.868 | 6.49E-12 | ++++++ |
| rs4562138 | 6 | 44126807 | T | 0.1937 | -6.87 | 6.42E-12 | ------ |
| rs12200733 | 6 | 44128121 | T | 0.18 | -6.904 | 5.05E-12 | ------ |
| rs302953 | 8 | 106512481 | T | 0.5642 | 10.504 | 8.26E-26 | ++++++ |
| rs4734869 | 8 | 106512828 | A | 0.2806 | -12.245 | 1.78E-34 | ------ |
| rs6988664 | 8 | 106513301 | A | 0.7187 | 12.408 | 2.38E-35 | ++++++ |
| rs57942103 | 8 | 106513461 | A | 0.7332 | 12.447 | 1.46E-35 | ++++++ |
| rs6469004 | 8 | 106514052 | A | 0.7217 | 12.5 | 7.47E-36 | ++++++ |
| rs6993681 | 8 | 106514091 | A | 0.7217 | 12.502 | 7.30E-36 | ++++++ |
| rs74434756 | 8 | 106514134 | I | 0.2783 | -12.506 | 6.89E-36 | ------ |
| rs72673716 | 8 | 106514155 | A | 0.2782 | -12.507 | 6.84E-36 | ------ |
| rs16873196 | 8 | 106514212 | A | 0.7217 | 12.533 | 4.91E-36 | ++++++ |
| rs6981223 | 8 | 106515026 | T | 0.7217 | 12.539 | 4.58E-36 | ++++++ |
| rs302958 | 8 | 106515115 | T | 0.5875 | -7.367 | 1.75E-13 | ------ |
| rs12680351 | 8 | 106515179 | T | 0.7982 | 12.166 | 4.73E-34 | ++++++ |
| rs12674625 | 8 | 106515898 | A | 0.7223 | 12.573 | 2.98E-36 | ++++++ |
| rs12678719 | 8 | 106516054 | C | 0.7216 | 12.575 | 2.89E-36 | ++++++ |
| rs2291192 | 8 | 106517072 | A | 0.2747 | -12.494 | 8.09E-36 | ------ |
| rs5893746 | 8 | 106517173 | I | 0.279 | -12.44 | 1.58E-35 | ------ |
| rs302959 | 8 | 106517641 | T | 0.5859 | -7.199 | 6.06E-13 | ------ |
| rs1868649 | 8 | 106517865 | A | 0.2774 | -12.573 | 2.97E-36 | ------ |
| rs6997142 | 8 | 106517876 | T | 0.7254 | 12.522 | 5.64E-36 | ++++++ |
| rs1868650 | 8 | 106517912 | T | 0.7226 | 12.573 | 2.96E-36 | ++++++ |
| rs6997293 | 8 | 106517928 | T | 0.7254 | 12.522 | 5.63E-36 | ++++++ |
| rs302962 | 8 | 106518153 | A | 0.4126 | 7.341 | 2.12E-13 | ++++++ |
| rs60350605 | 8 | 106518168 | I | 0.2762 | -12.513 | 6.37E-36 | ------ |
| rs16873231 | 8 | 106518235 | C | 0.7227 | 12.582 | 2.67E-36 | ++++++ |
| rs16873235 | 8 | 106518323 | A | 0.7252 | 12.512 | 6.43E-36 | ++++++ |
| rs302964 | 8 | 106518412 | T | 0.5116 | -7.106 | 1.20E-12 | +----- |
| rs7016378 | 8 | 106518817 | A | 0.7987 | 12.166 | 4.73E-34 | ++++++ |
| rs6982413 | 8 | 106518912 | T | 0.2738 | -12.535 | 4.82E-36 | ------ |
| rs215400 | 8 | 106519210 | T | 0.4973 | -7.058 | 1.69E-12 | +----- |
| rs7003261 | 8 | 106519305 | T | 0.7225 | 12.571 | 3.05E-36 | ++++++ |
| rs4734871 | 8 | 106520051 | A | 0.7431 | -6.234 | 4.53E-10 | ------ |
| rs35139970 | 8 | 106521078 | I | 0.3503 | -11.631 | 2.87E-31 | ------ |
| rs10094054 | 8 | 106521091 | A | 0.2029 | -10.968 | 5.42E-28 | ------ |
| rs7007968 | 8 | 106521845 | A | 0.7092 | 12.874 | 6.30E-38 | ++++++ |
| rs35213234 | 8 | 106522973 | I | 0.2917 | -12.846 | 9.09E-38 | ------ |
| rs10094510 | 8 | 106523185 | A | 0.2753 | -12.483 | 9.24E-36 | ------ |
| rs12680382 | 8 | 106524285 | A | 0.2901 | -12.707 | 5.43E-37 | ------ |
| rs10105733 | 8 | 106525861 | A | 0.7092 | 12.809 | 1.46E-37 | ++++++ |
| rs10093297 | 8 | 106526469 | T | 0.7092 | 12.803 | 1.58E-37 | ++++++ |
| rs150008884 | 8 | 106527260 | D | 0.291 | -12.85 | 8.63E-38 | ------ |
| rs16873287 | 8 | 106527975 | T | 0.7097 | 12.829 | 1.13E-37 | ++++++ |
| rs16873291 | 8 | 106528030 | T | 0.2901 | -12.794 | 1.76E-37 | ------ |
| rs35031575 | 8 | 106528643 | D | 0.2903 | -12.851 | 8.45E-38 | ------ |
| rs12676726 | 8 | 106528708 | T | 0.7238 | 12.504 | 7.09E-36 | ++++++ |
| rs10955397 | 8 | 106528826 | A | 0.6173 | -6.978 | 3.00E-12 | ------ |
| rs12679049 | 8 | 106528846 | A | 0.8002 | 12.196 | 3.26E-34 | ++++++ |
| rs12675041 | 8 | 106529045 | A | 0.3391 | -12.526 | 5.37E-36 | ------ |
| rs7837523 | 8 | 106529302 | A | 0.2426 | 6.019 | 1.76E-09 | +++++- |
| rs13267536 | 8 | 106529866 | A | 0.2091 | 6.015 | 1.80E-09 | ++++++ |
| rs4734872 | 8 | 106529881 | C | 0.7093 | 12.797 | 1.71E-37 | ++++++ |
| rs1375959 | 8 | 106530125 | A | 0.3822 | 6.853 | 7.22E-12 | ++++++ |
| rs10659560 | 8 | 106530507 | I | 0.7224 | -6.24 | 4.37E-10 | -----+ |
| rs4734119 | 8 | 106530523 | T | 0.7088 | 12.748 | 3.19E-37 | ++++++ |
| rs1157141 | 8 | 106531310 | A | 0.707 | 12.758 | 2.81E-37 | ++++++ |
| rs1157142 | 8 | 106531674 | A | 0.2907 | -12.794 | 1.77E-37 | ------ |
| rs1450163 | 8 | 106532122 | T | 0.6347 | 12.144 | 6.19E-34 | ++++++ |
| rs112472681 | 8 | 106532462 | I | 0.2858 | -12.611 | 1.83E-36 | ------ |
| rs6996138 | 8 | 106532477 | A | 0.7234 | 12.479 | 9.69E-36 | ++++++ |
| rs10955398 | 8 | 106533114 | A | 0.3842 | 6.803 | 1.03E-11 | ++++++ |
| rs4734873 | 8 | 106533330 | A | 0.2908 | -12.789 | 1.90E-37 | ------ |
| rs35916002 | 8 | 106533564 | D | 0.2757 | -12.393 | 2.86E-35 | ------ |
| rs7001868 | 8 | 106533619 | A | 0.7092 | 12.794 | 1.78E-37 | ++++++ |
| rs7836542 | 8 | 106534443 | T | 0.3648 | -12.108 | 9.60E-34 | ------ |
| rs7836585 | 8 | 106534599 | A | 0.7092 | 12.798 | 1.68E-37 | ++++++ |
| rs6469005 | 8 | 106534939 | T | 0.7119 | 12.897 | 4.66E-38 | ++++++ |
| rs2343298 | 8 | 106536253 | T | 0.3065 | 8.499 | 1.92E-17 | ++++++ |
| rs72673731 | 8 | 106536267 | T | 0.2392 | -14.104 | 3.59E-45 | ------ |
| rs7830388 | 8 | 106536683 | T | 0.5365 | 12.962 | 2.00E-38 | ++++++ |
| rs996488 | 8 | 106537714 | T | 0.4011 | -8.57 | 1.03E-17 | ------ |
| rs1450161 | 8 | 106541358 | A | 0.4228 | 9.893 | 4.45E-23 | ++++++ |
| rs200540748 | 8 | 106546504 | I | 0.469 | -5.703 | 1.18E-08 | ------ |
| rs28539851 | 8 | 106546724 | C | 0.7591 | 14.312 | 1.84E-46 | ++++++ |
| rs4734874 | 8 | 106547516 | T | 0.5896 | -10.52 | 6.95E-26 | ------ |
| rs16873346 | 8 | 106549481 | C | 0.2399 | -14.354 | 1.01E-46 | ------ |
| rs1901061 | 8 | 106550250 | A | 0.3928 | 9.75 | 1.84E-22 | ++++++ |
| rs4734875 | 8 | 106554737 | T | 0.7589 | 14.301 | 2.15E-46 | ++++++ |
| rs12680936 | 8 | 106554901 | A | 0.5887 | -10.534 | 6.02E-26 | ------ |
| rs1375955 | 8 | 106555039 | T | 0.3975 | 9.957 | 2.35E-23 | ++++++ |
| rs1349319 | 8 | 106556634 | A | 0.3932 | 9.927 | 3.18E-23 | ++++++ |
| rs16873365 | 8 | 106558235 | T | 0.1428 | -12.39 | 2.97E-35 | ------ |
| rs7008697 | 8 | 106559719 | A | 0.4815 | 8.248 | 1.61E-16 | ++++++ |
| rs4734122 | 8 | 106560370 | A | 0.4114 | 10.55 | 5.09E-26 | ++++++ |
| rs200575600 | 8 | 106561786 | D | 0.346 | -9.65 | 4.90E-22 | ------ |
| rs4734876 | 8 | 106561790 | T | 0.5824 | 11.971 | 5.02E-33 | ++++++ |
| rs202188628 | 8 | 106562174 | I | 0.5692 | -10.272 | 9.42E-25 | ------ |
| rs35446103 | 8 | 106563910 | I | 0.5862 | -10.602 | 2.93E-26 | ------ |
| rs147319748 | 8 | 106564180 | I | 0.6074 | -9.767 | 1.56E-22 | ------ |
| rs1375956 | 8 | 106564511 | T | 0.4119 | 10.506 | 8.14E-26 | ++++++ |
| rs28416651 | 8 | 106564652 | A | 0.4098 | -12.254 | 1.60E-34 | ------ |
| rs10089159 | 8 | 106564722 | A | 0.4129 | 10.547 | 5.25E-26 | ++++++ |
| rs71305160 | 8 | 106564889 | I | 0.5817 | -10.402 | 2.42E-25 | ------ |
| rs10092486 | 8 | 106564917 | C | 0.5867 | -10.55 | 5.08E-26 | ------ |
| rs10093110 | 8 | 106565414 | A | 0.4144 | -12.163 | 4.92E-34 | ------ |
| rs9886652 | 8 | 106570222 | A | 0.5585 | -10.785 | 4.04E-27 | ------ |
| rs34797610 | 8 | 106570300 | I | 0.5977 | -10.107 | 5.17E-24 | ------ |
| rs1868648 | 8 | 106570442 | A | 0.5588 | -10.891 | 1.27E-27 | ------ |
| rs34826779 | 8 | 106570964 | T | 0.2795 | -15.84 | 1.65E-56 | ------ |
| rs1375957 | 8 | 106571883 | T | 0.606 | -9.83 | 8.35E-23 | ------ |
| rs2343592 | 8 | 106572270 | A | 0.7175 | 15.854 | 1.32E-56 | ++++++ |
| rs7008006 | 8 | 106572854 | T | 0.414 | 10.596 | 3.10E-26 | ++++++ |
| rs3832566 | 8 | 106573524 | D | 0.278 | -15.763 | 5.58E-56 | ------ |
| rs3735953 | 8 | 106573578 | T | 0.5859 | -10.595 | 3.15E-26 | ------ |
| rs12216772 | 8 | 106573919 | T | 0.436 | -13.607 | 3.66E-42 | ------ |
| rs921335 | 8 | 106575332 | T | 0.5859 | -10.595 | 3.15E-26 | ------ |
| rs4734877 | 8 | 106578032 | A | 0.4132 | 10.625 | 2.28E-26 | ++++++ |
| rs72673751 | 8 | 106578940 | T | 0.8038 | 13.948 | 3.22E-44 | ++++++ |
| rs7832219 | 8 | 106578977 | T | 0.7101 | 15.864 | 1.13E-56 | ++++++ |
| rs6469006 | 8 | 106579063 | T | 0.586 | -10.595 | 3.15E-26 | ------ |
| rs10103526 | 8 | 106580607 | A | 0.414 | 10.589 | 3.36E-26 | ++++++ |
| rs6993696 | 8 | 106581284 | A | 0.4398 | -13.546 | 8.39E-42 | ------ |
| rs6993770 | 8 | 106581528 | A | 0.6982 | 15.835 | 1.79E-56 | ++++++ |
| rs4734879 | 8 | 106583124 | A | 0.701 | 15.655 | 3.06E-55 | ++++++ |
| rs4734880 | 8 | 106583398 | C | 0.5593 | -10.874 | 1.53E-27 | ------ |
| rs1470684 | 8 | 106583872 | A | 0.4403 | 10.851 | 1.98E-27 | ++++++ |
| rs7816615 | 8 | 106586094 | T | 0.4134 | 10.607 | 2.76E-26 | ++++++ |
| rs16873402 | 8 | 106589247 | T | 0.3215 | -14.878 | 4.58E-50 | ------ |
| rs1901062 | 8 | 106589534 | A | 0.5819 | -10.674 | 1.35E-26 | ------ |
| rs3217600 | 8 | 106589697 | I | 0.5799 | -10.523 | 6.75E-26 | ------ |
| rs34740151 | 8 | 106590642 | I | 0.583 | -10.584 | 3.54E-26 | ------ |
| rs6987377 | 8 | 106590684 | A | 0.4149 | 10.562 | 4.48E-26 | ++++++ |
| rs200508901 | 8 | 106590704 | D | 0.2505 | -14.438 | 2.98E-47 | ------ |
| rs4541868 | 8 | 106590705 | A | 0.2504 | -14.474 | 1.77E-47 | ------ |
| rs4602861 | 8 | 106590706 | A | 0.7499 | 14.42 | 3.87E-47 | ++++++ |
| rs201279889 | 8 | 106590706 | I | 0.2547 | -14.444 | 2.73E-47 | ------ |
| rs201312530 | 8 | 106590848 | I | 0.5344 | -9.314 | 1.23E-20 | ------ |
| rs200070434 | 8 | 106590851 | D | 0.5332 | -9.392 | 5.88E-21 | ------ |
| rs2343594 | 8 | 106590852 | A | 0.4378 | 8.689 | 3.66E-18 | ++++++ |
| rs2343595 | 8 | 106591207 | C | 0.5593 | 10.933 | 8.05E-28 | ++++++ |
| rs16873415 | 8 | 106591802 | A | 0.56 | 10.947 | 6.89E-28 | ++++++ |
| rs16873418 | 8 | 106592145 | A | 0.6774 | 14.868 | 5.35E-50 | ++++++ |
| rs66489920 | 8 | 106592539 | T | 0.6764 | 14.831 | 9.18E-50 | ++++++ |
| rs2343596 | 8 | 106593207 | A | 0.344 | -13.478 | 2.12E-41 | ------ |
| rs7013321 | 8 | 106593558 | A | 0.4671 | -11.75 | 7.08E-32 | ------ |
| rs6995272 | 8 | 106593662 | T | 0.4595 | 11.705 | 1.20E-31 | ++++++ |
| rs68054037 | 8 | 106656381 | A | 0.8155 | -5.476 | 4.35E-08 | ------ |
| rs10966869 | 9 | 2543283 | A | 0.0362 | -5.988 | 2.12E-09 | ---?-- |
| rs542859 | 9 | 2558513 | T | 0.2952 | 5.921 | 3.20E-09 | ++++++ |
| rs75656289 | 9 | 2558858 | T | 0.312 | 5.569 | 2.57E-08 | ++++++ |
| rs494035 | 9 | 2560442 | T | 0.2959 | 5.913 | 3.36E-09 | ++++++ |
| rs580571 | 9 | 2560670 | T | 0.3205 | 6.429 | 1.28E-10 | +++-++ |
| rs7043199 | 9 | 2621145 | A | 0.2066 | -5.924 | 3.14E-09 | ------ |
| rs28669967 | 9 | 2626916 | C | 0.7008 | 5.617 | 1.94E-08 | ++++++ |
| rs1877480 | 9 | 2658311 | T | 0.1546 | -5.898 | 3.67E-09 | +----- |
| rs4741754 | 9 | 2658775 | A | 0.224 | 6.905 | 5.01E-12 | -+++++ |
| rs4741756 | 9 | 2668187 | A | 0.7111 | 14.61 | 2.42E-48 | -+++++ |
| rs12156533 | 9 | 2668292 | A | 0.6769 | 15.833 | 1.85E-56 | -+++++ |
| rs201609595 | 9 | 2669625 | D | 0.2277 | -11.976 | 4.76E-33 | +----- |
| rs139014291 | 9 | 2669626 | D | 0.2299 | -12.015 | 2.96E-33 | +----- |
| rs139933989 | 9 | 2671418 | D | 0.2574 | -10.961 | 5.86E-28 | +----- |
| rs77541516 | 9 | 2672204 | C | 0.9478 | 6.497 | 8.21E-11 | ++++++ |
| rs79348841 | 9 | 2672306 | T | 0.9479 | 6.5 | 8.02E-11 | ++++++ |
| rs7027187 | 9 | 2672442 | T | 0.9384 | 6.606 | 3.94E-11 | ++++++ |
| rs6475918 | 9 | 2672461 | T | 0.9419 | 6.751 | 1.47E-11 | ++++++ |
| rs6475919 | 9 | 2672809 | T | 0.3757 | -17.976 | 3.03E-72 | ------ |
| rs7041786 | 9 | 2673052 | A | 0.069 | -5.961 | 2.51E-09 | ------ |
| rs7031045 | 9 | 2673086 | T | 0.8779 | 8.817 | 1.18E-18 | ++++++ |
| rs1542358 | 9 | 2673393 | T | 0.0691 | -5.956 | 2.59E-09 | ------ |
| rs12347630 | 9 | 2673801 | A | 0.0631 | -5.904 | 3.54E-09 | ------ |
| rs6475920 | 9 | 2673933 | A | 0.3803 | -18.034 | 1.05E-72 | ------ |
| rs6475921 | 9 | 2674000 | T | 0.8782 | 8.996 | 2.34E-19 | ++++++ |
| rs7859818 | 9 | 2674110 | A | 0.937 | 5.945 | 2.76E-09 | ++++++ |
| rs6475922 | 9 | 2674318 | T | 0.8785 | 9.007 | 2.12E-19 | ++++++ |
| rs6475923 | 9 | 2674571 | A | 0.0732 | -5.869 | 4.39E-09 | ------ |
| rs7874073 | 9 | 2674864 | A | 0.0697 | -5.978 | 2.26E-09 | ------ |
| rs6475925 | 9 | 2675158 | C | 0.8933 | 5.792 | 6.97E-09 | ++++++ |
| rs56836371 | 9 | 2675385 | T | 0.0694 | -5.959 | 2.54E-09 | ------ |
| rs10967470 | 9 | 2675698 | A | 0.7586 | 13.527 | 1.09E-41 | ++++++ |
| rs11793970 | 9 | 2675728 | C | 0.0634 | -5.892 | 3.83E-09 | ------ |
| rs7867894 | 9 | 2675867 | T | 0.742 | 13.095 | 3.51E-39 | -+++++ |
| rs1020651 | 9 | 2676360 | A | 0.6185 | 17.953 | 4.57E-72 | ++++++ |
| rs1041574 | 9 | 2676728 | C | 0.8834 | 5.725 | 1.04E-08 | ++++++ |
| rs1020652 | 9 | 2677012 | A | 0.6177 | 17.984 | 2.58E-72 | ++++++ |
| rs7029840 | 9 | 2677429 | A | 0.0687 | -5.909 | 3.43E-09 | ------ |
| rs7029311 | 9 | 2677743 | A | 0.0619 | -5.714 | 1.11E-08 | ------ |
| rs7046015 | 9 | 2677787 | A | 0.9305 | 5.886 | 3.96E-09 | ++++++ |
| rs6475926 | 9 | 2678059 | T | 0.9313 | 5.899 | 3.67E-09 | ++++++ |
| rs6475927 | 9 | 2678077 | A | 0.0679 | -5.779 | 7.49E-09 | ------ |
| rs7847455 | 9 | 2678427 | A | 0.9313 | 5.888 | 3.91E-09 | ++++++ |
| rs16909038 | 9 | 2678958 | C | 0.9318 | 5.887 | 3.94E-09 | ++++++ |
| rs11794349 | 9 | 2679427 | A | 0.9024 | 6.131 | 8.72E-10 | ++++++ |
| rs10125071 | 9 | 2679579 | T | 0.5923 | 16.713 | 1.05E-62 | ++++++ |
| rs11788260 | 9 | 2679711 | A | 0.0553 | -6.639 | 3.16E-11 | ------ |
| rs11789806 | 9 | 2680077 | A | 0.0556 | -6.659 | 2.77E-11 | ------ |
| rs2039420 | 9 | 2680092 | C | 0.5978 | 16.683 | 1.73E-62 | ++++++ |
| rs10117473 | 9 | 2680425 | C | 0.7856 | 13.245 | 4.80E-40 | ++++++ |
| rs111552651 | 9 | 2680655 | C | 0.0552 | -6.679 | 2.40E-11 | ------ |
| rs79460761 | 9 | 2680877 | T | 0.9382 | 5.751 | 8.85E-09 | ++++++ |
| rs5023561 | 9 | 2680879 | A | 0.8472 | 6.653 | 2.87E-11 | ++++++ |
| rs4317630 | 9 | 2681025 | T | 0.5888 | 16.815 | 1.90E-63 | ++++++ |
| rs10967492 | 9 | 2681175 | A | 0.2153 | -13.427 | 4.19E-41 | ------ |
| rs6475931 | 9 | 2681427 | T | 0.1543 | -6.734 | 1.65E-11 | ------ |
| rs7870360 | 9 | 2681430 | T | 0.8458 | 6.692 | 2.20E-11 | ++++++ |
| rs6475932 | 9 | 2681470 | T | 0.8464 | 6.667 | 2.61E-11 | ++++++ |
| rs4339695 | 9 | 2681559 | A | 0.4101 | -16.828 | 1.51E-63 | ------ |
| rs7855003 | 9 | 2682038 | C | 0.2576 | -13.215 | 7.16E-40 | ------ |
| rs11794855 | 9 | 2682322 | T | 0.1175 | -9.127 | 7.07E-20 | ------ |
| rs10738758 | 9 | 2682355 | A | 0.6141 | 18.169 | 9.05E-74 | ++++++ |
| rs113798386 | 9 | 2682570 | T | 0.0552 | -6.705 | 2.01E-11 | ------ |
| rs7856084 | 9 | 2682943 | C | 0.407 | -16.836 | 1.33E-63 | ------ |
| rs7048329 | 9 | 2683132 | A | 0.8457 | 6.578 | 4.76E-11 | ++++++ |
| rs10967512 | 9 | 2683939 | C | 0.2135 | -13.353 | 1.14E-40 | ------ |
| rs10967515 | 9 | 2684117 | T | 0.8129 | 9.085 | 1.04E-19 | ++++++ |
| rs10967517 | 9 | 2684176 | T | 0.1865 | -9.212 | 3.20E-20 | ------ |
| rs10757628 | 9 | 2684656 | T | 0.2137 | -13.185 | 1.06E-39 | ------ |
| rs113533325 | 9 | 2684985 | T | 0.0915 | -9.601 | 7.92E-22 | ------ |
| rs58078557 | 9 | 2685277 | A | 0.1568 | -11.419 | 3.37E-30 | ------ |
| rs10967526 | 9 | 2685764 | A | 0.2136 | -13.161 | 1.48E-39 | ------ |
| rs10114279 | 9 | 2686236 | A | 0.2197 | -13.239 | 5.22E-40 | ------ |
| rs7044724 | 9 | 2686260 | C | 0.0648 | -5.725 | 1.04E-08 | ------ |
| rs7030781 | 9 | 2686273 | A | 0.5749 | 20.385 | 2.30E-92 | ++++++ |
| rs10116375 | 9 | 2686336 | A | 0.2147 | -13.125 | 2.38E-39 | ------ |
| rs10967529 | 9 | 2686536 | T | 0.7715 | 12.442 | 1.54E-35 | ++++++ |
| rs10116513 | 9 | 2686871 | C | 0.2251 | -13.215 | 7.21E-40 | ------ |
| rs11788351 | 9 | 2687426 | T | 0.9316 | 5.78 | 7.46E-09 | ++++++ |
| rs55992105 | 9 | 2687539 | T | 0.1292 | -8.257 | 1.49E-16 | ------ |
| rs11789745 | 9 | 2687669 | T | 0.0677 | -5.714 | 1.10E-08 | ------ |
| rs10115227 | 9 | 2687794 | T | 0.8021 | 12.65 | 1.11E-36 | ++++++ |
| rs78459751 | 9 | 2687795 | A | 0.5618 | 19.811 | 2.37E-87 | ++++++ |
| rs6475939 | 9 | 2687860 | T | 0.3281 | -15.384 | 2.08E-53 | ------ |
| rs200375999 | 9 | 2688539 | D | 0.0621 | -5.722 | 1.05E-08 | ------ |
| rs140649323 | 9 | 2688540 | D | 0.0628 | -5.705 | 1.16E-08 | ------ |
| rs10125245 | 9 | 2689244 | T | 0.2139 | -13.159 | 1.51E-39 | ------ |
| rs7047154 | 9 | 2689301 | T | 0.891 | 5.474 | 4.39E-08 | ++++++ |
| rs28473277 | 9 | 2689657 | T | 0.2151 | -13.069 | 4.93E-39 | ------ |
| rs10757631 | 9 | 2690295 | C | 0.5155 | 19.084 | 3.40E-81 | ++++++ |
| rs10757632 | 9 | 2690374 | C | 0.4404 | -18.649 | 1.28E-77 | ------ |
| rs10812468 | 9 | 2690656 | T | 0.187 | -12.671 | 8.57E-37 | ------ |
| rs10812469 | 9 | 2690657 | A | 0.1836 | -12.549 | 4.00E-36 | ------ |
| rs10812470 | 9 | 2690701 | C | 0.7527 | 11.529 | 9.47E-31 | -+++++ |
| rs77260291 | 9 | 2690710 | A | 0.1941 | -10.5 | 8.66E-26 | ------ |
| rs10812471 | 9 | 2690980 | T | 0.2123 | -13.206 | 8.13E-40 | ------ |
| rs10812472 | 9 | 2691095 | C | 0.7877 | 13.204 | 8.29E-40 | ++++++ |
| rs10738760 | 9 | 2691186 | A | 0.5147 | 19.15 | 9.67E-82 | ++++++ |
| rs10812473 | 9 | 2691329 | A | 0.2123 | -13.203 | 8.43E-40 | ------ |
| rs10812474 | 9 | 2691446 | A | 0.7856 | 13.126 | 2.33E-39 | ++++++ |
| rs75395320 | 9 | 2691528 | T | 0.9364 | 5.737 | 9.64E-09 | ++++++ |
| rs10812475 | 9 | 2691546 | A | 0.7876 | 13.204 | 8.29E-40 | ++++++ |
| rs10122524 | 9 | 2691667 | T | 0.3129 | -13.722 | 7.45E-43 | ------ |
| rs10122587 | 9 | 2691951 | T | 0.2842 | -15.398 | 1.69E-53 | ------ |
| rs11788193 | 9 | 2692393 | A | 0.0724 | -5.716 | 1.09E-08 | ------ |
| rs2375981 | 9 | 2692583 | C | 0.5378 | 20.38 | 2.52E-92 | ++++++ |
| rs2375980 | 9 | 2692622 | C | 0.5945 | 17.52 | 1.02E-68 | ++++++ |
| rs11787847 | 9 | 2692773 | A | 0.9288 | 5.675 | 1.38E-08 | ++++++ |
| rs10124200 | 9 | 2693559 | T | 0.2038 | -13.514 | 1.29E-41 | ------ |
| rs748787 | 9 | 2694201 | A | 0.0672 | -5.759 | 8.49E-09 | ------ |
| rs10812481 | 9 | 2694596 | T | 0.7656 | 13.755 | 4.72E-43 | ++++++ |
| rs10967570 | 9 | 2694711 | A | 0.2339 | -13.785 | 3.13E-43 | ------ |
| rs930811 | 9 | 2696555 | A | 0.4481 | 7.985 | 1.41E-15 | ++++++ |
| rs10967580 | 9 | 2696738 | A | 0.4621 | 6.33 | 2.46E-10 | ++++++ |
| rs7856053 | 9 | 2697370 | T | 0.4251 | -7.797 | 6.32E-15 | ------ |
| rs7853140 | 9 | 2697549 | A | 0.5069 | -7.084 | 1.40E-12 | ------ |
| rs7467643 | 9 | 2698837 | A | 0.4928 | -5.477 | 4.34E-08 | ------ |
| rs1884441 | 9 | 2699004 | A | 0.8433 | -6.883 | 5.85E-12 | ------ |
| rs7048719 | 9 | 2699451 | T | 0.2248 | 7.846 | 4.31E-15 | ++++++ |
| rs7034098 | 9 | 2699606 | A | 0.7764 | -7.623 | 2.48E-14 | ------ |
| rs10812493 | 9 | 2700102 | T | 0.2258 | 7.648 | 2.04E-14 | ++++++ |
| rs10812496 | 9 | 2700128 | A | 0.2259 | 7.661 | 1.84E-14 | ++++++ |
| rs60832147 | 9 | 2700269 | I | 0.224 | 7.578 | 3.50E-14 | ++++++ |
| rs12339372 | 9 | 2700282 | A | 0.777 | -7.611 | 2.72E-14 | ------ |
| rs10967598 | 9 | 2700883 | A | 0.1343 | 6.449 | 1.13E-10 | ++++++ |
| rs1869594 | 9 | 2701954 | C | 0.4518 | -8.569 | 1.04E-17 | ------ |
| rs6475949 | 9 | 2702351 | A | 0.8262 | -5.696 | 1.23E-08 | ------ |
| rs7855332 | 9 | 2702703 | T | 0.5033 | -6.248 | 4.15E-10 | ------ |
| rs7031875 | 9 | 2703729 | T | 0.4276 | -7.497 | 6.53E-14 | ------ |
| rs10757637 | 9 | 2704141 | A | 0.6161 | 7.179 | 7.00E-13 | ++++++ |
| rs7032456 | 9 | 2704399 | A | 0.4376 | -7.298 | 2.93E-13 | ------ |
| rs1412693 | 9 | 2705874 | T | 0.4331 | -7.621 | 2.52E-14 | ------ |
| rs4084415 | 9 | 2705970 | A | 0.4232 | -7.372 | 1.69E-13 | ------ |
| rs75245372 | 9 | 2706443 | A | 0.142 | 6.884 | 5.84E-12 | ++++++ |
| rs1889109 | 9 | 2706850 | C | 0.8516 | -6.72 | 1.82E-11 | ------ |
| rs1823903 | 9 | 2707420 | A | 0.4232 | -7.368 | 1.73E-13 | ------ |
| rs3085942 | 9 | 2707850 | I | 0.5408 | 7.948 | 1.89E-15 | ++++++ |
| rs12345016 | 9 | 2708258 | C | 0.5205 | -6.8 | 1.04E-11 | ------ |
| rs1454630 | 9 | 2708587 | A | 0.7646 | -6.378 | 1.79E-10 | ------ |
| rs1454631 | 9 | 2708657 | T | 0.5702 | 7.309 | 2.69E-13 | ++++++ |
| rs12346258 | 9 | 2709183 | T | 0.1547 | 6.577 | 4.80E-11 | ++++++ |
| rs897165 | 9 | 2710537 | T | 0.5499 | 7.975 | 1.53E-15 | ++++++ |
| rs12686646 | 9 | 2710619 | A | 0.1486 | 6.619 | 3.62E-11 | ++++++ |
| rs7029012 | 9 | 2717698 | C | 0.4267 | -6.482 | 9.03E-11 | ------ |
| rs10967705 | 9 | 2717922 | C | 0.4258 | -6.658 | 2.77E-11 | ------ |
| rs12237048 | 9 | 2718534 | C | 0.5305 | -7.282 | 3.29E-13 | ------ |
| rs10812526 | 9 | 2721249 | A | 0.5636 | -5.664 | 1.48E-08 | ------ |
| rs10967728 | 9 | 2721794 | C | 0.4377 | 5.668 | 1.44E-08 | ++++++ |
| rs1026355 | 9 | 2722884 | C | 0.512 | 6.411 | 1.44E-10 | ++++-+ |
| rs1006698 | 9 | 2725283 | A | 0.4876 | -6.468 | 9.95E-11 | ----+- |
| rs10733413 | 9 | 2734710 | T | 0.4981 | -6.414 | 1.42E-10 | ----+- |
| rs7048820 | 9 | 2735053 | T | 0.5003 | -6.407 | 1.49E-10 | ----+- |
| rs7045626 | 9 | 2735203 | A | 0.6573 | -6.559 | 5.43E-11 | ----+- |
| rs7029926 | 9 | 2735253 | C | 0.6653 | -6.326 | 2.51E-10 | ----+- |
| rs7029249 | 9 | 2735415 | A | 0.3333 | 6.317 | 2.67E-10 | ++++-+ |
| rs1454632 | 9 | 2735818 | A | 0.2604 | 5.691 | 1.26E-08 | ++++++ |
| rs1454633 | 9 | 2735910 | T | 0.7416 | -5.859 | 4.65E-09 | ------ |
| rs1454634 | 9 | 2736026 | A | 0.2586 | 5.857 | 4.71E-09 | ++++++ |
| rs1470944 | 9 | 2736312 | T | 0.7395 | -5.676 | 1.38E-08 | ------ |
| rs7034474 | 9 | 2736347 | T | 0.2195 | 5.914 | 3.35E-09 | ++++++ |
| rs12237278 | 9 | 2737095 | A | 0.2633 | 6.017 | 1.78E-09 | ++++++ |
| rs10812562 | 9 | 2737140 | A | 0.263 | 6.006 | 1.90E-09 | ++++++ |
| rs1470945 | 9 | 2737697 | A | 0.2643 | 6.017 | 1.77E-09 | ++++++ |
| rs10812564 | 9 | 2738294 | T | 0.2133 | 5.806 | 6.41E-09 | ++++++ |
| rs10812565 | 9 | 2738413 | T | 0.7782 | -5.732 | 9.92E-09 | ------ |
| rs4741760 | 9 | 2739846 | T | 0.2091 | 5.863 | 4.55E-09 | ++++++ |
| rs2375964 | 9 | 2740175 | T | 0.2092 | 5.876 | 4.20E-09 | ++++++ |
| rs10738768 | 9 | 2741196 | A | 0.2105 | 5.882 | 4.05E-09 | ++++++ |
| rs7850096 | 9 | 2741728 | T | 0.5759 | -6.043 | 1.52E-09 | ----+- |
| rs2034763 | 9 | 2742608 | T | 0.4647 | 6.704 | 2.03E-11 | ++++-+ |
| rs2034764 | 9 | 2742771 | T | 0.5135 | -7.077 | 1.48E-12 | ----+- |
| rs4741762 | 9 | 2743269 | A | 0.2093 | 6.147 | 7.92E-10 | ++++++ |
| rs10812579 | 9 | 2746834 | T | 0.4437 | 6.874 | 6.25E-12 | ++++++ |
| rs7033112 | 9 | 2748501 | T | 0.4734 | 7.359 | 1.85E-13 | ++++++ |
| rs12380321 | 9 | 2749343 | T | 0.9317 | 6.164 | 7.08E-10 | ++++++ |
| rs7875640 | 9 | 2757207 | T | 0.6188 | -7.76 | 8.52E-15 | ------ |
| rs4348558 | 9 | 2761137 | T | 0.3661 | 8.325 | 8.45E-17 | ++++++ |
| rs77933099 | 9 | 2762262 | A | 0.4551 | -8.589 | 8.77E-18 | ------ |
| rs11793471 | 9 | 2762263 | A | 0.4526 | -8.656 | 4.90E-18 | ------ |
| rs6476000 | 9 | 2762303 | T | 0.5305 | 8.714 | 2.94E-18 | ++++++ |
| rs958505 | 9 | 2762721 | A | 0.2079 | 7.18 | 6.95E-13 | ++++++ |
| rs7849358 | 9 | 2763315 | T | 0.6287 | -7.884 | 3.16E-15 | ------ |
| rs10738776 | 9 | 2763894 | A | 0.4796 | -8.888 | 6.21E-19 | ------ |
| rs2168134 | 9 | 2765753 | T | 0.3523 | 8.372 | 5.67E-17 | ++++++ |
| rs7033566 | 9 | 2766462 | C | 0.6305 | -8.265 | 1.40E-16 | ------ |
| rs10738777 | 9 | 2768619 | T | 0.6433 | -8.416 | 3.90E-17 | ------ |
| rs2375862 | 9 | 2770776 | T | 0.5118 | 8.591 | 8.59E-18 | ++++++ |
| rs4520217 | 9 | 2770887 | T | 0.3845 | 7.808 | 5.83E-15 | ++++++ |
| rs10738778 | 9 | 2773573 | T | 0.2351 | 6.19 | 6.00E-10 | ++++++ |
| rs2168135 | 9 | 2774237 | T | 0.6039 | -7.499 | 6.45E-14 | ------ |
| rs4741766 | 9 | 2774758 | A | 0.5113 | 8.581 | 9.43E-18 | ++++++ |
| rs10812639 | 9 | 2776015 | A | 0.4012 | 7.398 | 1.39E-13 | ++++++ |
| rs10738780 | 9 | 2776401 | C | 0.5202 | 8.525 | 1.53E-17 | ++++++ |
| rs10757675 | 9 | 2776518 | T | 0.3852 | 7.782 | 7.13E-15 | ++++++ |
| rs10125062 | 9 | 2777062 | T | 0.4832 | -8.646 | 5.34E-18 | ------ |
| rs7027337 | 9 | 2777366 | C | 0.6005 | -7.587 | 3.28E-14 | ------ |
| rs10812641 | 9 | 2777384 | A | 0.4832 | -8.736 | 2.43E-18 | ------ |
| rs4740699 | 9 | 2778867 | A | 0.3972 | 7.602 | 2.91E-14 | ++++++ |
| rs4741767 | 9 | 2779020 | T | 0.403 | 7.615 | 2.63E-14 | ++++++ |
| rs2889224 | 9 | 2779504 | T | 0.6338 | -6.251 | 4.08E-10 | ------ |
| rs2375861 | 9 | 2779515 | T | 0.5426 | 8.934 | 4.13E-19 | ++++++ |
| rs2375860 | 9 | 2779525 | A | 0.5585 | -7.377 | 1.62E-13 | ------ |
| rs3214827 | 9 | 2780496 | I | 0.4724 | -8.594 | 8.40E-18 | ------ |
| rs201357444 | 9 | 2780498 | I | 0.4251 | -7.75 | 9.17E-15 | ------ |
| rs7035082 | 9 | 2780922 | A | 0.3811 | 7.606 | 2.84E-14 | ++++++ |
| rs7850616 | 9 | 2781918 | A | 0.5985 | -7.067 | 1.59E-12 | ------ |
| rs10122155 | 9 | 2782458 | A | 0.5094 | 8.452 | 2.87E-17 | ++++++ |
| rs10122896 | 9 | 2782774 | T | 0.5098 | 8.471 | 2.42E-17 | ++++++ |
| rs4623490 | 9 | 2783113 | C | 0.6209 | -7.667 | 1.76E-14 | ------ |
| rs1060812 | 9 | 2783788 | T | 0.3782 | 7.654 | 1.95E-14 | ++++++ |
| rs1551410 | 9 | 2786648 | C | 0.4158 | 7.763 | 8.29E-15 | ++++++ |
| rs7049072 | 9 | 2787594 | C | 0.5123 | 8.515 | 1.66E-17 | ++++++ |
| rs10757686 | 9 | 2787856 | A | 0.51 | 8.517 | 1.64E-17 | ++++++ |
| rs7869344 | 9 | 2789195 | A | 0.377 | 7.84 | 4.49E-15 | ++++++ |
| rs1454635 | 9 | 2790307 | T | 0.6016 | -7.574 | 3.61E-14 | ------ |
| rs10121371 | 9 | 2790559 | C | 0.4001 | 7.581 | 3.43E-14 | ++++++ |
| rs7865866 | 9 | 2790935 | A | 0.4865 | -8.636 | 5.82E-18 | ------ |
| rs10125412 | 9 | 2791753 | A | 0.5195 | 8.396 | 4.61E-17 | ++++++ |
| rs10113893 | 9 | 2791828 | T | 0.5166 | 8.541 | 1.33E-17 | ++++++ |
| rs10738782 | 9 | 2791926 | T | 0.36 | 7.243 | 4.38E-13 | ++++++ |
| rs10812668 | 9 | 2791971 | A | 0.6068 | -7.421 | 1.16E-13 | ------ |
| rs10812670 | 9 | 2792195 | C | 0.4143 | 6.304 | 2.91E-10 | ++++++ |
| rs7032942 | 9 | 2792552 | A | 0.5047 | 8.409 | 4.14E-17 | ++++++ |
| rs201545715 | 9 | 2792734 | D | 0.4994 | -8.077 | 6.66E-16 | ------ |
| rs141349847 | 9 | 2792761 | D | 0.4891 | -8.269 | 1.35E-16 | ------ |
| rs10046804 | 9 | 2793342 | A | 0.4907 | 8.912 | 5.01E-19 | ++++++ |
| rs7037223 | 9 | 2793539 | A | 0.3554 | 8.01 | 1.15E-15 | ++++++ |
| rs6476018 | 9 | 2793659 | T | 0.3553 | 8.013 | 1.12E-15 | ++++++ |
| rs10118386 | 9 | 2794077 | A | 0.4898 | -8.801 | 1.36E-18 | ------ |
| rs7041159 | 9 | 2794270 | A | 0.3562 | 8.032 | 9.57E-16 | ++++++ |
| rs10757691 | 9 | 2794338 | T | 0.4914 | 8.835 | 9.98E-19 | ++++++ |
| rs7857106 | 9 | 2795748 | A | 0.4922 | -8.935 | 4.07E-19 | ------ |
| rs6476021 | 9 | 2795824 | A | 0.3734 | 7.956 | 1.78E-15 | ++++++ |
| rs6476022 | 9 | 2795953 | A | 0.3591 | 8.209 | 2.22E-16 | ++++++ |
| rs6476023 | 9 | 2795985 | C | 0.4975 | -9.115 | 7.90E-20 | ------ |
| rs138162183 | 9 | 2796135 | D | 0.4948 | -8.996 | 2.33E-19 | ------ |
| rs2376312 | 9 | 2796498 | T | 0.783 | -7.441 | 9.96E-14 | ------ |
| rs7033946 | 9 | 2796867 | A | 0.5068 | -9.272 | 1.83E-20 | ------ |
| rs7037204 | 9 | 2797082 | A | 0.5009 | -8.985 | 2.60E-19 | ------ |
| rs7038516 | 9 | 2797394 | C | 0.3591 | 8.136 | 4.10E-16 | ++++++ |
| rs7041669 | 9 | 2798112 | C | 0.5313 | -9.425 | 4.28E-21 | ------ |
| rs7027886 | 9 | 2798358 | A | 0.3564 | 7.912 | 2.53E-15 | ++++++ |
| rs7042249 | 9 | 2798583 | A | 0.525 | -9.3 | 1.41E-20 | ------ |
| rs7031607 | 9 | 2798612 | T | 0.5008 | 8.867 | 7.54E-19 | ++++++ |
| rs870652 | 9 | 2800854 | A | 0.715 | -6.731 | 1.69E-11 | ------ |
| rs7032686 | 9 | 2801858 | T | 0.536 | -7.549 | 4.40E-14 | ------ |
| rs10812719 | 9 | 2809260 | C | 0.486 | -8.186 | 2.70E-16 | ------ |
| rs7850317 | 9 | 2809548 | A | 0.4068 | 7.106 | 1.19E-12 | ++++++ |
| rs2270888 | 9 | 2811505 | A | 0.4942 | -8.389 | 4.91E-17 | ------ |
| rs3739460 | 9 | 2814615 | A | 0.4811 | 6.719 | 1.83E-11 | ++++++ |
| rs10481516 | 9 | 2821498 | A | 0.5008 | 7.809 | 5.79E-15 | ++++++ |
| rs6476038 | 9 | 2823380 | A | 0.4736 | -8.094 | 5.77E-16 | ------ |
| rs10757713 | 9 | 2824000 | A | 0.4883 | 7.005 | 2.47E-12 | ++++++ |
| rs10968361 | 9 | 2825965 | T | 0.4865 | 7.474 | 7.80E-14 | ++++++ |
| rs10738791 | 9 | 2826283 | T | 0.493 | 7.453 | 9.10E-14 | ++++++ |
| rs4439183 | 9 | 2826344 | C | 0.4865 | 7.584 | 3.34E-14 | ++++++ |
| rs3858032 | 9 | 2826674 | T | 0.6028 | -6.504 | 7.82E-11 | ------ |
| rs10757715 | 9 | 2827752 | T | 0.5001 | 7.225 | 5.01E-13 | ++++++ |
| rs7036752 | 9 | 2828060 | A | 0.4511 | 6.497 | 8.20E-11 | ++++++ |
| rs2173904 | 9 | 2828765 | C | 0.4501 | 6.501 | 7.96E-11 | ++++++ |
| rs10738792 | 9 | 2829308 | A | 0.4515 | 6.525 | 6.78E-11 | ++++++ |
| rs2292000 | 9 | 2831149 | A | 0.4502 | 6.453 | 1.10E-10 | ++++++ |
| rs10125846 | 9 | 2831891 | T | 0.4549 | 6.32 | 2.61E-10 | ++++++ |
| rs6476047 | 9 | 2832663 | T | 0.4682 | -7.437 | 1.03E-13 | ------ |
| rs1875439 | 9 | 2832914 | C | 0.5805 | -6.519 | 7.08E-11 | ------ |
| rs10812760 | 9 | 2833107 | A | 0.4775 | 6.573 | 4.93E-11 | ++++++ |
| rs10217194 | 9 | 2833267 | T | 0.4758 | 6.515 | 7.25E-11 | ++++++ |
| rs201760651 | 9 | 2833691 | D | 0.4768 | -7.553 | 4.26E-14 | ------ |
| rs3830413 | 9 | 2833692 | D | 0.4784 | -7.603 | 2.88E-14 | ------ |
| rs111736617 | 9 | 2834833 | I | 0.5278 | -5.94 | 2.86E-09 | ------ |
| rs1393043 | 9 | 2835137 | C | 0.4137 | 6.024 | 1.70E-09 | ++++++ |
| rs1393044 | 9 | 2835181 | A | 0.4124 | 5.994 | 2.05E-09 | ++++++ |
| rs35637097 | 9 | 2835562 | D | 0.5857 | -6.019 | 1.76E-09 | ------ |
| rs10812770 | 9 | 2835579 | A | 0.4936 | -7.171 | 7.44E-13 | ------ |
| rs1875440 | 9 | 2835973 | T | 0.4742 | 5.982 | 2.20E-09 | ++++++ |
| rs1875441 | 9 | 2835981 | T | 0.4674 | 6.062 | 1.35E-09 | ++++++ |
| rs10121671 | 9 | 2838107 | T | 0.4842 | -7.792 | 6.62E-15 | ------ |
| rs3824350 | 9 | 2839608 | C | 0.4736 | 6.264 | 3.76E-10 | ++++++ |
| rs3824351 | 9 | 2839826 | T | 0.5081 | 7.153 | 8.46E-13 | ++++++ |
| rs10116824 | 9 | 2840829 | T | 0.5049 | 7.094 | 1.31E-12 | ++++++ |
| rs10733432 | 9 | 2841423 | C | 0.4962 | -7.13 | 1.01E-12 | ------ |
| rs10733433 | 9 | 2841493 | A | 0.4734 | 6.004 | 1.93E-09 | ++++++ |
| rs4741768 | 9 | 2841700 | T | 0.5077 | 7.352 | 1.95E-13 | ++++++ |
| rs10812784 | 9 | 2843647 | T | 0.5362 | 7.147 | 8.85E-13 | ++++++ |
| rs4741769 | 9 | 2847195 | A | 0.491 | -8.072 | 6.91E-16 | ------ |
| rs11790146 | 9 | 2851881 | C | 0.5581 | 7.385 | 1.52E-13 | ++++++ |
| rs10822143 | 10 | 64887856 | T | 0.5072 | 6.927 | 4.30E-12 | ++++++ |
| rs150230220 | 10 | 64901792 | I | 0.3198 | 6.534 | 6.40E-11 | ++++++ |
| rs7073746 | 10 | 64904071 | A | 0.5186 | -6.997 | 2.61E-12 | ------ |
| rs67345368 | 10 | 64907581 | D | 0.4755 | 7.066 | 1.60E-12 | ++++++ |
| rs35751397 | 10 | 64914016 | I | 0.4783 | 6.908 | 4.91E-12 | ++++++ |
| rs13095 | 10 | 64914372 | C | 0.4822 | 6.966 | 3.26E-12 | ++++++ |
| rs71461799 | 10 | 64914518 | D | 0.4793 | 6.924 | 4.38E-12 | ++++++ |
| rs35562567 | 10 | 64916030 | D | 0.4466 | 6.676 | 2.45E-11 | ++++++ |
| rs35447916 | 10 | 64933592 | I | 0.4827 | 6.813 | 9.58E-12 | ++++++ |
| rs10822145 | 10 | 64934548 | T | 0.4633 | 6.76 | 1.38E-11 | ++++++ |
| rs10995455 | 10 | 64936361 | A | 0.3093 | 6.462 | 1.04E-10 | ++++++ |
| rs10733789 | 10 | 64948684 | T | 0.683 | -6.589 | 4.42E-11 | ------ |
| rs10761723 | 10 | 64955581 | T | 0.4832 | 6.856 | 7.09E-12 | ++++++ |
| rs7895472 | 10 | 64961412 | A | 0.483 | 6.875 | 6.20E-12 | ++++++ |
| rs7895610 | 10 | 64961659 | T | 0.4791 | 6.836 | 8.14E-12 | ++++++ |
| rs4379723 | 10 | 64963449 | T | 0.5175 | -6.861 | 6.83E-12 | ------ |
| rs10822149 | 10 | 64987412 | A | 0.4822 | 6.863 | 6.72E-12 | ++++++ |
| rs201778637 | 10 | 64988924 | I | 0.384 | 5.805 | 6.45E-09 | ++++++ |
| rs7916868 | 10 | 64988931 | A | 0.4944 | -6.015 | 1.80E-09 | ------ |
| rs10761727 | 10 | 64995493 | T | 0.4813 | 6.827 | 8.67E-12 | ++++++ |
| rs10761729 | 10 | 64995604 | C | 0.4869 | 6.912 | 4.77E-12 | ++++++ |
| rs4399232 | 10 | 64999490 | T | 0.4811 | 6.826 | 8.72E-12 | ++++++ |
| rs7902343 | 10 | 65005399 | T | 0.4568 | 6.499 | 8.11E-11 | ++++++ |
| rs10995477 | 10 | 65010672 | T | 0.5209 | -6.948 | 3.70E-12 | ------ |
| rs4400684 | 10 | 65012687 | A | 0.5206 | -6.968 | 3.21E-12 | ------ |
| rs4454603 | 10 | 65012750 | T | 0.4781 | 6.95 | 3.65E-12 | ++++++ |
| rs4595427 | 10 | 65012944 | A | 0.5206 | -6.968 | 3.22E-12 | ------ |
| rs4405189 | 10 | 65013935 | A | 0.5226 | -6.949 | 3.67E-12 | ------ |
| rs7088799 | 10 | 65016174 | T | 0.5722 | -7.673 | 1.68E-14 | ------ |
| rs7896910 | 10 | 65016721 | A | 0.3114 | 6.833 | 8.33E-12 | ++++++ |
| rs7098181 | 10 | 65027143 | T | 0.4272 | 7.644 | 2.11E-14 | ++++++ |
| rs10761731 | 10 | 65027610 | A | 0.5727 | -7.643 | 2.12E-14 | ------ |
| rs9787438 | 10 | 65038030 | C | 0.4274 | 7.672 | 1.69E-14 | ++++++ |
| rs7080386 | 10 | 65048306 | A | 0.4279 | 7.703 | 1.33E-14 | ++++++ |
| rs7075195 | 10 | 65050659 | A | 0.5735 | -7.672 | 1.69E-14 | ------ |
| rs10761737 | 10 | 65052205 | T | 0.578 | -7.73 | 1.07E-14 | ------ |
| rs7084707 | 10 | 65052542 | T | 0.429 | 7.746 | 9.49E-15 | ++++++ |
| rs5785566 | 10 | 65054786 | I | 0.4824 | 7.052 | 1.77E-12 | ++++++ |
| rs10822153 | 10 | 65056813 | A | 0.48 | 6.961 | 3.38E-12 | ++++++ |
| rs10761739 | 10 | 65062008 | C | 0.4287 | 7.726 | 1.11E-14 | ++++++ |
| rs7073753 | 10 | 65062820 | T | 0.426 | 7.673 | 1.68E-14 | ++++++ |
| rs199545673 | 10 | 65063835 | I | 0.4149 | 7.831 | 4.84E-15 | ++++++ |
| rs74506613 | 10 | 65063836 | D | 0.4155 | 7.836 | 4.64E-15 | ++++++ |
| rs10761741 | 10 | 65066186 | T | 0.4287 | 7.783 | 7.10E-15 | ++++++ |
| rs7922587 | 10 | 65069614 | T | 0.5174 | -7.021 | 2.21E-12 | ------ |
| rs7070296 | 10 | 65070438 | A | 0.4794 | 7.024 | 2.15E-12 | ++++++ |
| rs10822155 | 10 | 65071215 | A | 0.4232 | 7.846 | 4.30E-15 | ++++++ |
| rs7090111 | 10 | 65077994 | C | 0.5721 | -7.842 | 4.43E-15 | ------ |
| rs10822156 | 10 | 65080727 | T | 0.5207 | -7.054 | 1.73E-12 | ------ |
| rs10761742 | 10 | 65085048 | A | 0.521 | -7.111 | 1.15E-12 | ------ |
| rs10822158 | 10 | 65094383 | A | 0.5228 | -7.093 | 1.32E-12 | ------ |
| rs10740115 | 10 | 65094990 | A | 0.4773 | 7.079 | 1.46E-12 | ++++++ |
| rs10740116 | 10 | 65094992 | T | 0.5232 | -7.072 | 1.52E-12 | ------ |
| rs10822159 | 10 | 65096250 | T | 0.4278 | 7.922 | 2.35E-15 | ++++++ |
| rs10740118 | 10 | 65101207 | C | 0.4278 | 7.922 | 2.34E-15 | ++++++ |
| rs7896518 | 10 | 65104500 | A | 0.5727 | -7.894 | 2.93E-15 | ------ |
| rs10822160 | 10 | 65112796 | T | 0.521 | -7.135 | 9.68E-13 | ------ |
| rs10822161 | 10 | 65118203 | A | 0.4792 | 7.099 | 1.26E-12 | ++++++ |
| rs12355784 | 10 | 65121565 | A | 0.4793 | 7.19 | 6.47E-13 | ++++++ |
| rs10822163 | 10 | 65124098 | C | 0.518 | -7.065 | 1.61E-12 | ------ |
| rs6479896 | 10 | 65126832 | T | 0.5205 | -7.098 | 1.26E-12 | ------ |
| rs10822164 | 10 | 65127258 | A | 0.4733 | 7.004 | 2.49E-12 | ++++++ |
| rs10761750 | 10 | 65128619 | A | 0.4794 | 7.098 | 1.27E-12 | ++++++ |
| rs10546984 | 10 | 65131440 | D | 0.4831 | 6.925 | 4.36E-12 | ++++++ |
| rs2393967 | 10 | 65133156 | A | 0.6974 | -6.795 | 1.08E-11 | ------ |
| rs7923609 | 10 | 65133822 | A | 0.5206 | -7.111 | 1.15E-12 | ------ |
| rs2893919 | 10 | 65134778 | A | 0.4793 | 7.092 | 1.32E-12 | ++++++ |
| rs2393966 | 10 | 65134814 | T | 0.5207 | -7.092 | 1.32E-12 | ------ |
| rs7076310 | 10 | 65135672 | A | 0.4793 | 7.092 | 1.32E-12 | ++++++ |
| rs4310508 | 10 | 65138573 | A | 0.5208 | -7.092 | 1.32E-12 | ------ |
| rs7910927 | 10 | 65138910 | T | 0.5208 | -7.093 | 1.32E-12 | ------ |
| rs2393969 | 10 | 65140440 | A | 0.5203 | -7.09 | 1.34E-12 | ------ |
| rs201528108 | 10 | 65149088 | D | 0.5238 | -7.089 | 1.36E-12 | ------ |
| rs9414801 | 10 | 65149089 | A | 0.5239 | -6.76 | 1.38E-11 | ------ |
| rs7095571 | 10 | 65150959 | T | 0.523 | -7.144 | 9.05E-13 | ------ |
| rs10761751 | 10 | 65154885 | A | 0.5251 | -7.042 | 1.89E-12 | ------ |
| rs150036478 | 10 | 65158772 | D | 0.4762 | 7.09 | 1.34E-12 | ++++++ |
| rs10761752 | 10 | 65160321 | T | 0.5226 | -7.113 | 1.14E-12 | ------ |
| rs7912893 | 10 | 65162000 | A | 0.4753 | 7.153 | 8.46E-13 | ++++++ |
| rs7896783 | 10 | 65162153 | A | 0.4776 | 7.106 | 1.19E-12 | ++++++ |
| rs10822168 | 10 | 65165184 | A | 0.4676 | 6.892 | 5.50E-12 | ++++++ |
| rs10761756 | 10 | 65172328 | T | 0.4769 | 7.095 | 1.29E-12 | ++++++ |
| rs10761758 | 10 | 65172747 | A | 0.5307 | -6.806 | 1.00E-11 | ------ |
| rs150374366 | 10 | 65172799 | D | 0.4725 | 7.045 | 1.86E-12 | ++++++ |
| rs7909269 | 10 | 65177766 | A | 0.4758 | 7.09 | 1.34E-12 | ++++++ |
| rs200711715 | 10 | 65179151 | I | 0.4733 | 6.965 | 3.28E-12 | ++++++ |
| rs7077580 | 10 | 65180885 | A | 0.4778 | 7.098 | 1.26E-12 | ++++++ |
| rs7923544 | 10 | 65182256 | T | 0.5199 | -7.077 | 1.47E-12 | ------ |
| rs10761762 | 10 | 65184717 | T | 0.5219 | -7.104 | 1.22E-12 | ------ |
| rs10761763 | 10 | 65188318 | T | 0.5219 | -7.103 | 1.22E-12 | ------ |
| rs10761766 | 10 | 65190327 | A | 0.4781 | 7.103 | 1.22E-12 | ++++++ |
| rs7924036 | 10 | 65191645 | T | 0.4967 | 6.431 | 1.27E-10 | ++++++ |
| rs3740331 | 10 | 65192288 | A | 0.4781 | 7.104 | 1.22E-12 | ++++++ |
| rs71463522 | 10 | 65193329 | D | 0.4426 | -6.682 | 2.36E-11 | ------ |
| rs200376292 | 10 | 65193341 | D | 0.4264 | -6.863 | 6.75E-12 | ------ |
| rs201670954 | 10 | 65193342 | D | 0.4314 | -6.715 | 1.88E-11 | ------ |
| rs3999089 | 10 | 65203808 | A | 0.5223 | -7.133 | 9.81E-13 | ------ |
| rs3956912 | 10 | 65205881 | T | 0.4776 | 7.086 | 1.38E-12 | ++++++ |
| rs10509186 | 10 | 65207018 | T | 0.4794 | 7.095 | 1.29E-12 | ++++++ |
| rs7085621 | 10 | 65208926 | T | 0.5207 | -7.099 | 1.25E-12 | ------ |
| rs10740125 | 10 | 65209609 | T | 0.5207 | -7.1 | 1.25E-12 | ------ |
| rs10740126 | 10 | 65210935 | A | 0.5202 | -7.1 | 1.25E-12 | ------ |
| rs7092784 | 10 | 65214749 | T | 0.4793 | 7.105 | 1.21E-12 | ++++++ |
| rs35290825 | 10 | 65224200 | I | 0.4843 | 7.05 | 1.79E-12 | ++++++ |
| rs10761771 | 10 | 65230164 | T | 0.5207 | -7.141 | 9.28E-13 | ------ |
| rs10733792 | 10 | 65232539 | A | 0.521 | -7.129 | 1.01E-12 | ------ |
| rs10761772 | 10 | 65235829 | T | 0.479 | 7.133 | 9.82E-13 | ++++++ |
| rs7909960 | 10 | 65239177 | A | 0.4782 | 7.117 | 1.10E-12 | ++++++ |
| rs7915779 | 10 | 65244244 | C | 0.4782 | 7.125 | 1.04E-12 | ++++++ |
| rs2393977 | 10 | 65247609 | A | 0.5207 | -7.119 | 1.09E-12 | ------ |
| rs10740129 | 10 | 65250808 | A | 0.4791 | 7.114 | 1.13E-12 | ++++++ |
| 10:65260508 | 10 | 65260508 | I | 0.4935 | 6.447 | 1.14E-10 | ++++++ |
| rs34524635 | 10 | 65261176 | D | 0.4684 | 6.841 | 7.84E-12 | ++++++ |
| rs199648319 | 10 | 65261179 | D | 0.4064 | 6.95 | 3.65E-12 | ++++++ |
| rs2893923 | 10 | 65261184 | T | 0.313 | 6.859 | 6.94E-12 | ++++++ |
| rs10509189 | 10 | 65264126 | T | 0.5208 | -7.13 | 1.01E-12 | ------ |
| rs4486511 | 10 | 65264266 | T | 0.4792 | 7.13 | 1.01E-12 | ++++++ |
| rs9971352 | 10 | 65265108 | A | 0.5207 | -7.128 | 1.02E-12 | ------ |
| rs35048644 | 10 | 65265705 | D | 0.4772 | 7.195 | 6.23E-13 | ++++++ |
| rs10740131 | 10 | 65271488 | A | 0.5199 | -6.858 | 6.97E-12 | ------ |
| rs10761778 | 10 | 65273782 | A | 0.5234 | -7.022 | 2.19E-12 | ------ |
| rs10761779 | 10 | 65274927 | A | 0.5204 | -7.106 | 1.20E-12 | ------ |
| rs7082470 | 10 | 65277026 | A | 0.4797 | 7.109 | 1.17E-12 | ++++++ |
| rs7075901 | 10 | 65280994 | A | 0.4719 | 7.12 | 1.08E-12 | ++++++ |
| rs7085018 | 10 | 65286667 | T | 0.5149 | -7.197 | 6.15E-13 | ------ |
| rs79576555 | 10 | 65287064 | A | 0.5236 | -6.835 | 8.22E-12 | ------ |
| rs5785580 | 10 | 65290254 | D | 0.4917 | 6.239 | 4.41E-10 | ++++++ |
| rs7920036 | 10 | 65293860 | T | 0.5187 | -7.155 | 8.36E-13 | ------ |
| rs7920058 | 10 | 65293924 | T | 0.5186 | -7.159 | 8.14E-13 | ------ |
| rs12770839 | 10 | 65297720 | A | 0.3022 | 6.853 | 7.21E-12 | ++++++ |
| rs7897379 | 10 | 65301725 | T | 0.517 | -7.066 | 1.60E-12 | ------ |
| rs202062785 | 10 | 65308749 | D | 0.3905 | 6.045 | 1.50E-09 | ++++++ |
| rs9919429 | 10 | 65313819 | A | 0.5214 | 6.279 | 3.40E-10 | ++++++ |
| rs2163188 | 10 | 65314711 | C | 0.4912 | -6.336 | 2.36E-10 | ------ |
| rs2393984 | 10 | 65314971 | A | 0.5106 | 6.3 | 2.97E-10 | ++++++ |
| rs6479905 | 10 | 65315231 | A | 0.509 | 6.337 | 2.34E-10 | ++++++ |
| rs10740134 | 10 | 65315433 | T | 0.5167 | -7.089 | 1.35E-12 | ------ |
| rs7919685 | 10 | 65315800 | T | 0.4894 | -6.317 | 2.67E-10 | ------ |
| rs67344189 | 10 | 65316235 | D | 0.4704 | -5.872 | 4.31E-09 | ------ |
| rs201876756 | 10 | 65316381 | I | 0.4771 | -6.577 | 4.79E-11 | ------ |
| rs74502455 | 10 | 65316437 | C | 0.5231 | -7.062 | 1.64E-12 | ------ |
| rs12247907 | 10 | 65317045 | C | 0.4895 | -6.35 | 2.16E-10 | ------ |
| rs7070761 | 10 | 65317056 | A | 0.5108 | 6.346 | 2.21E-10 | ++++++ |
| rs5785581 | 10 | 65318502 | I | 0.4688 | 6.59 | 4.40E-11 | ++++++ |
| rs7898861 | 10 | 65319678 | T | 0.5102 | 6.371 | 1.88E-10 | ++++++ |
| rs2393986 | 10 | 65320006 | A | 0.5181 | -7.097 | 1.28E-12 | ------ |
| rs12245149 | 10 | 65321147 | A | 0.4897 | -6.363 | 1.98E-10 | ------ |
| rs12245367 | 10 | 65321464 | T | 0.486 | -6.237 | 4.45E-10 | ------ |
| rs36027145 | 10 | 65322464 | I | 0.4881 | -6.18 | 6.41E-10 | ------ |
| rs7899657 | 10 | 65323265 | A | 0.4854 | -6.221 | 4.93E-10 | ------ |
| rs10733793 | 10 | 65323809 | T | 0.5126 | -6.984 | 2.88E-12 | ------ |
| rs4746203 | 10 | 65323997 | T | 0.4818 | -6.162 | 7.17E-10 | ------ |
| rs10822181 | 10 | 65325126 | A | 0.4853 | -6.237 | 4.46E-10 | ------ |
| rs10822182 | 10 | 65325478 | A | 0.4874 | 6.979 | 2.97E-12 | ++++++ |
| rs200667913 | 10 | 65326990 | D | 0.4427 | 6.908 | 4.90E-12 | ++++++ |
| rs68005787 | 10 | 65326991 | D | 0.4789 | 6.931 | 4.19E-12 | ++++++ |
| rs4572027 | 10 | 65328050 | A | 0.4862 | 6.555 | 5.56E-11 | ++++++ |
| rs4746204 | 10 | 65328518 | T | 0.4853 | -6.228 | 4.73E-10 | ------ |
| rs4633333 | 10 | 65328539 | C | 0.5154 | -7.009 | 2.41E-12 | ------ |
| rs7907451 | 10 | 65328740 | A | 0.4853 | -6.229 | 4.71E-10 | ------ |
| rs4746205 | 10 | 65329518 | A | 0.5153 | 6.267 | 3.67E-10 | ++++++ |
| rs10740136 | 10 | 65329622 | A | 0.5129 | -7.005 | 2.47E-12 | ------ |
| rs7100409 | 10 | 65330195 | A | 0.4855 | -6.252 | 4.05E-10 | ------ |
| rs7100413 | 10 | 65330208 | T | 0.5149 | 6.217 | 5.07E-10 | ++++++ |
| rs3847325 | 10 | 65331511 | C | 0.4852 | -6.185 | 6.19E-10 | ------ |
| rs3847326 | 10 | 65331859 | A | 0.5169 | 6.191 | 5.98E-10 | ++++++ |
| rs6479908 | 10 | 65333648 | C | 0.5286 | 5.862 | 4.57E-09 | ++++++ |
| rs7090758 | 10 | 65335315 | T | 0.5289 | 5.886 | 3.95E-09 | ++++++ |
| rs7077256 | 10 | 65336185 | A | 0.5295 | 5.882 | 4.05E-09 | ++++++ |
| rs10761786 | 10 | 65336207 | T | 0.4975 | -6.611 | 3.83E-11 | ------ |
| rs7902616 | 10 | 65336373 | A | 0.4705 | -5.882 | 4.06E-09 | ------ |
| rs10822183 | 10 | 65337141 | T | 0.5295 | 5.873 | 4.27E-09 | ++++++ |
| rs10822184 | 10 | 65337153 | T | 0.4975 | -6.602 | 4.06E-11 | ------ |
| rs7910951 | 10 | 65338304 | A | 0.539 | 5.825 | 5.72E-09 | ++++++ |
| rs7911761 | 10 | 65338753 | C | 0.5382 | 5.855 | 4.77E-09 | ++++++ |
| rs12768534 | 10 | 65340897 | A | 0.4695 | -5.907 | 3.48E-09 | ------ |
| rs10822186 | 10 | 65350383 | A | 0.4965 | -6.62 | 3.59E-11 | ------ |
| rs10761787 | 10 | 65353755 | A | 0.4957 | -6.569 | 5.07E-11 | ------ |
| rs7895549 | 10 | 65357438 | A | 0.4956 | 6.281 | 3.37E-10 | ++++++ |
| rs1896995 | 10 | 65365385 | T | 0.503 | 5.731 | 1.00E-08 | ++++++ |

- - *Coded: Coded allele (D, I, and R respectively represent Deletion, Insertion, and Reference). ^†^Coded AF: Coded allele Frequency. ^‡^Direction: Effect direction of the coded allele on VEGF levels; the sequence of the direction follows the alphabetical order of cohorts (AGES, Cilento, FHS, OGP, PIVUS, VB)
