# Supplementary material for: Six Novel Loci Associated with Circulating VEGF Levels Identified by a Meta-analysis of Genome-Wide Association Studies
Source: PLoS Genet. 2016 Feb 24;12(2):e1005874. doi: 10.1371/journal.pgen.1005874 (PMC4766012; doi:10.1371/journal.pgen.1005874)
Supplement: S3 Table — (DOCX) [file pgen.1005874.s005.docx]

**Supplementary Table3.**

| **VEGF**  **associated SNP** | **eSNP** | **Chr** | **position** | **r^2^ eSNP-VEGF associated**  **SNP** | **Tissue** | **ArrayID** | **Transcript** | **eSNP.P** |
| --- | --- | --- | --- | --- | --- | --- | --- | --- |
| *cis-eQTLs* |  |  |  |  |  |  |  |  |
| rs74506613 | rs7073746 | 10 | 64904071 | 0.83 | LCL in asthmatics (Liang 1kg) | 223650_s_at | NRBF2 | 3.53E-24 |
| rs74506613 | rs13095 | 10 | 64914372 | 0.83 | LCL in asthmatics (Liang 1kg) | 223650_s_at | NRBF2 | 3.22E-24 |
| rs74506613 | rs1935 | 10 | 64927823 | 0.84 | CD14+ monocytes (24h LPS stimulated) | 6960523 | JMJD1C | 4.85E-05 |
| rs74506613 | rs1935 | 10 | 64927823 | 0.84 | CD14+ monocytes (2h LPS stimulated) | 6960523 | JMJD1C | 7.37E-05 |
| rs74506613 | rs1935 | 10 | 64927823 | 0.84 | CD14+ monocytes (IFNg stimulated) | 6960523 | JMJD1C | 5.40E-11 |
| rs74506613 | rs1935 | 10 | 64927823 | 0.84 | CD14+ monocytes (untreated) | 6960523 | JMJD1C | 1.20E-06 |
| rs74506613 | rs1935 | 10 | 64927823 | 0.84 | LCL in asthmatics (Liang 1kg) | 223650_s_at | NRBF2 | 3.27E-24 |
| rs74506613 | rs1935 | 10 | 64927823 | 0.84 | Whole blood (DeepSAGESeq) | 10_64914411 | NRBF2 | 1.50E-12 |
| rs74506613 | rs1935 | 10 | 64927823 | 0.84 | Dendritic cells after Mycobacterium tuberculosis infection |  | NRBF2 | 6.74E-08 |
| rs74506613 | rs10761723 | 10 | 64955581 | 0.84 | LCL in asthmatics (Liang 1kg) | 223650_s_at | NRBF2 | 5.03E-24 |
| rs74506613 | rs7895472 | 10 | 64961412 | 0.84 | LCL in asthmatics (Liang 1kg) | 223650_s_at | NRBF2 | 5.17E-24 |
| rs74506613 | rs7895610 | 10 | 64961659 | 0.84 | LCL in asthmatics (Liang 1kg) | 223650_s_at | NRBF2 | 5.18E-24 |
| rs74506613 | rs4379723 | 10 | 64963449 | 0.84 | Lymph | hmm19663-S | JMJD1C | 2.94E-04 |
| rs74506613 | rs4379723 | 10 | 64963449 | 0.84 | LCL in asthmatics (Liang 1kg) | 223650_s_at | NRBF2 | 5.19E-24 |
| rs74506613 | rs10822149 | 10 | 64987412 | 0.84 | LCL in asthmatics (Liang 1kg) | 223650_s_at | NRBF2 | 5.04E-24 |
| rs74506613 | rs10761727 | 10 | 64995493 | 0.84 | LCL in asthmatics (Liang 1kg) | 223650_s_at | NRBF2 | 4.98E-24 |
| rs74506613 | rs10761729 | 10 | 64995604 | 0.83 | LCL in asthmatics (Liang 1kg) | 223650_s_at | NRBF2 | 4.98E-24 |
| rs74506613 | rs4399232 | 10 | 64999490 | 0.84 | LCL in asthmatics (Liang 1kg) | 223650_s_at | NRBF2 | 4.92E-24 |
| rs74506613 | rs10995477 | 10 | 65010672 | 0.87 | LCL in asthmatics (Liang 1kg) | 223650_s_at | NRBF2 | 4.82E-24 |
| rs74506613 | rs4400684 | 10 | 65012687 | 0.88 | LCL in asthmatics (Liang 1kg) | 223650_s_at | NRBF2 | 4.80E-24 |
| rs74506613 | rs4454603 | 10 | 65012750 | 0.87 | LCL in asthmatics (Liang 1kg) | 223650_s_at | NRBF2 | 4.80E-24 |
| rs74506613 | rs4595427 | 10 | 65012944 | 0.88 | LCL in asthmatics (Liang 1kg) | 223650_s_at | NRBF2 | 4.78E-24 |
| rs74506613 | rs4405189 | 10 | 65013935 | 0.87 | LCL in asthmatics (Liang 1kg) | 223650_s_at | NRBF2 | 4.66E-24 |
| rs74506613 | rs7088799 | 10 | 65016174 | 0.97 | LCL in asthmatics (Liang 1kg) | 223650_s_at | NRBF2 | 4.71E-24 |
| rs74506613 | rs7098181 | 10 | 65027143 | 0.96 | LCL in asthmatics (Liang 1kg) | 223650_s_at | NRBF2 | 4.48E-24 |
| rs74506613 | rs10761731 | 10 | 65027610 | 0.96 | LCL in asthmatics (Liang 1kg) | 223650_s_at | NRBF2 | 4.48E-24 |
| rs74506613 | rs9787438 | 10 | 65038030 | 0.96 | LCL in asthmatics (Liang 1kg) | 223650_s_at | NRBF2 | 4.41E-24 |
| rs74506613 | rs7080386 | 10 | 65048306 | 0.97 | LCL in asthmatics (Liang 1kg) | 223650_s_at | NRBF2 | 7.94E-23 |
| rs74506613 | rs7075195 | 10 | 65050659 | 0.96 | LCL in asthmatics (Liang 1kg) | 223650_s_at | NRBF2 | 8.06E-23 |
| rs74506613 | rs10761737 | 10 | 65052205 | 0.83 | LCL in asthmatics (Liang 1kg) | 223650_s_at | NRBF2 | 7.92E-23 |
| rs74506613 | rs7084707 | 10 | 65052542 | 0.97 | LCL in asthmatics (Liang 1kg) | 223650_s_at | NRBF2 | 8.07E-23 |
| rs74506613 | rs10822153 | 10 | 65056813 | 0.88 | LCL in asthmatics (Liang 1kg) | 223650_s_at | NRBF2 | 4.31E-24 |
| rs74506613 | rs7073753 | 10 | 65062820 | 0.96 | LCL in asthmatics (Liang 1kg) | 223650_s_at | NRBF2 | 7.77E-23 |
| rs74506613 | rs10761741 | 10 | 65066186 | 0.97 | Whole blood (Schramm et al.) | ILMN_1719344 | NRBF2 | 1.22E-38 |
| rs74506613 | rs7922587 | 10 | 65069614 | 0.86 | LCL in asthmatics (Liang 1kg) | 223650_s_at | NRBF2 | 7.73E-23 |
| rs74506613 | rs7070296 | 10 | 65070438 | 0.88 | LCL in asthmatics (Liang 1kg) | 223650_s_at | NRBF2 | 7.78E-23 |
| rs74506613 | rs10822155 | 10 | 65071215 | 0.85 | LCL in asthmatics (Liang 1kg) | 223650_s_at | NRBF2 | 7.73E-23 |
| rs74506613 | rs7090111 | 10 | 65077994 | 0.96 | LCL in asthmatics (Liang 1kg) | 223650_s_at | NRBF2 | 7.73E-23 |
| rs74506613 | rs10822156 | 10 | 65080727 | 0.88 | LCL in asthmatics (Liang 1kg) | 223650_s_at | NRBF2 | 7.75E-23 |
| rs74506613 | rs10761742 | 10 | 65085048 | 0.87 | LCL in asthmatics (Liang 1kg) | 223650_s_at | NRBF2 | 4.30E-24 |
| rs74506613 | rs10822158 | 10 | 65094383 | 0.87 | LCL in asthmatics (Liang 1kg) | 223650_s_at | NRBF2 | 8.00E-23 |
| rs74506613 | rs10822159 | 10 | 65096250 | 0.96 | LCL in asthmatics (Liang 1kg) | 223650_s_at | NRBF2 | 8.10E-23 |
| rs74506613 | rs7896518 | 10 | 65104500 | 0.96 | LCL in asthmatics (Liang 1kg) | 223650_s_at | NRBF2 | 8.32E-23 |
| rs74506613 | rs10822160 | 10 | 65112796 | 0.88 | LCL in asthmatics (Liang 1kg) | 223650_s_at | NRBF2 | 4.89E-24 |
| rs74506613 | rs10822161 | 10 | 65118203 | 0.87 | LCL in asthmatics (Liang 1kg) | 223650_s_at | NRBF2 | 4.93E-24 |
| rs74506613 | rs10822163 | 10 | 65124098 | 0.87 | LCL in asthmatics (Liang 1kg) | 223650_s_at | NRBF2 | 5.08E-24 |
| rs74506613 | rs6479896 | 10 | 65126832 | 0.88 | LCL in asthmatics (Liang 1kg) | 223650_s_at | NRBF2 | 4.92E-24 |
| rs74506613 | rs10822164 | 10 | 65127258 | 0.86 | LCL in asthmatics (Liang 1kg) | 223650_s_at | NRBF2 | 4.92E-24 |
| rs74506613 | rs10761750 | 10 | 65128619 | 0.88 | LCL in asthmatics (Liang 1kg) | 223650_s_at | NRBF2 | 4.92E-24 |
| rs74506613 | rs7923609 | 10 | 65133822 | 0.88 | CD14+ monocytes (24h LPS stimulated) | 6960523 | JMJD1C | 7.45E-05 |
| rs74506613 | rs7923609 | 10 | 65133822 | 0.88 | CD14+ monocytes (2h LPS stimulated) | 6960523 | JMJD1C | 1.04E-04 |
| rs74506613 | rs7923609 | 10 | 65133822 | 0.88 | CD14+ monocytes (IFNg stimulated) | 6960523 | JMJD1C | 1.93E-10 |
| rs74506613 | rs7923609 | 10 | 65133822 | 0.88 | CD14+ monocytes (untreated) | 6960523 | JMJD1C | 6.93E-07 |
| rs74506613 | rs7923609 | 10 | 65133822 | 0.88 | Lymph | hmm19663-S | JMJD1C | 4.25E-04 |
| rs74506613 | rs7923609 | 10 | 65133822 | 0.88 | LCL in asthmatics (Liang 1kg) | 223650_s_at | NRBF2 | 5.09E-24 |
| rs74506613 | rs2893919 | 10 | 65134778 | 0.88 | LCL in asthmatics (Liang 1kg) | 223650_s_at | NRBF2 | 4.88E-24 |
| rs74506613 | rs2893919 | 10 | 65134778 | 0.88 | RNAseq (HapMapLCL)+DeepSage (blood) | ENST00000435510 | NRBF2 | 7.97E-13 |
| rs74506613 | rs2393966 | 10 | 65134814 | 0.88 | LCL in asthmatics (Liang 1kg) | 223650_s_at | NRBF2 | 4.87E-24 |
| rs74506613 | rs7076310 | 10 | 65135672 | 0.88 | LCL in asthmatics (Liang 1kg) | 223650_s_at | NRBF2 | 4.85E-24 |
| rs74506613 | rs4310508 | 10 | 65138573 | 0.87 | LCL in asthmatics (Liang 1kg) | 223650_s_at | NRBF2 | 4.81E-24 |
| rs74506613 | rs7910927 | 10 | 65138910 | 0.88 | LCL in asthmatics (Liang 1kg) | 223650_s_at | NRBF2 | 4.81E-24 |
| rs74506613 | rs7095571 | 10 | 65150959 | 0.81 | LCL in asthmatics (Liang 1kg) | 223650_s_at | NRBF2 | 4.60E-24 |
| rs74506613 | rs10761751 | 10 | 65154885 | 0.81 | LCL in asthmatics (Liang 1kg) | 223650_s_at | NRBF2 | 4.64E-24 |
| rs74506613 | rs10761752 | 10 | 65160321 | 0.81 | LCL in asthmatics (Liang 1kg) | 223650_s_at | NRBF2 | 4.70E-24 |
| rs74506613 | rs7912893 | 10 | 65162000 | 0.81 | LCL in asthmatics (Liang 1kg) | 223650_s_at | NRBF2 | 4.77E-24 |
| rs74506613 | rs7896783 | 10 | 65162153 | 0.81 | CD14+ monocytes (24h LPS stimulated) | 6960523 | JMJD1C | 5.14E-05 |
| rs74506613 | rs7896783 | 10 | 65162153 | 0.81 | CD14+ monocytes (2h LPS stimulated) | 6960523 | JMJD1C | 1.49E-04 |
| rs74506613 | rs7896783 | 10 | 65162153 | 0.81 | CD14+ monocytes (IFNg stimulated) | 6960523 | JMJD1C | 1.85E-10 |
| rs74506613 | rs7896783 | 10 | 65162153 | 0.81 | CD14+ monocytes (untreated) | 6960523 | JMJD1C | 7.30E-07 |
| rs74506613 | rs7896783 | 10 | 65162153 | 0.81 | LCL in asthmatics (Liang 1kg) | 223650_s_at | NRBF2 | 4.79E-24 |
| rs74506613 | rs7896783 | 10 | 65162153 | 0.81 | Monocytes (CD14+) | f90lDU9EJ_k_E7nnL8 | REEP3 | 5.83E-04 |
| rs74506613 | rs10761756 | 10 | 65172328 | 0.85 | LCL in asthmatics (Liang 1kg) | 223650_s_at | NRBF2 | 4.97E-24 |
| rs74506613 | rs10761758 | 10 | 65172747 | 0.84 | LCL in asthmatics (Liang 1kg) | 223650_s_at | NRBF2 | 5.02E-24 |
| rs74506613 | rs7909269 | 10 | 65177766 | 0.84 | LCL in asthmatics (Liang 1kg) | 223650_s_at | NRBF2 | 5.09E-24 |
| rs74506613 | rs7077580 | 10 | 65180885 | 0.85 | LCL in asthmatics (Liang 1kg) | 223650_s_at | NRBF2 | 5.19E-24 |
| rs74506613 | rs7923544 | 10 | 65182256 | 0.81 | LCL in asthmatics (Liang 1kg) | 223650_s_at | NRBF2 | 5.21E-24 |
| rs74506613 | rs10761762 | 10 | 65184717 | 0.81 | LCL in asthmatics (Liang 1kg) | 223650_s_at | NRBF2 | 5.32E-24 |
| rs74506613 | rs10761763 | 10 | 65188318 | 0.81 | LCL in asthmatics (Liang 1kg) | 223650_s_at | NRBF2 | 6.31E-24 |
| rs74506613 | rs10761766 | 10 | 65190327 | 0.81 | LCL in asthmatics (Liang 1kg) | 223650_s_at | NRBF2 | 6.51E-24 |
| rs74506613 | rs3740331 | 10 | 65192288 | 0.81 | LCL in asthmatics (Liang 1kg) | 223650_s_at | NRBF2 | 6.77E-24 |
| rs74506613 | rs3956912 | 10 | 65205881 | 0.80 | LCL in asthmatics (Liang 1kg) | 223650_s_at | NRBF2 | 7.40E-24 |
| rs74506613 | rs10509186 | 10 | 65207018 | 0.81 | Lymph | hmm19663-S | JMJD1C | 1.33E-04 |
| rs74506613 | rs10509186 | 10 | 65207018 | 0.81 | LCL in asthmatics (Liang 1kg) | 223650_s_at | NRBF2 | 7.41E-24 |
| rs74506613 | rs7085621 | 10 | 65208926 | 0.81 | LCL in asthmatics (Liang 1kg) | 223650_s_at | NRBF2 | 7.55E-24 |
| rs74506613 | rs10740125 | 10 | 65209609 | 0.81 | LCL in asthmatics (Liang 1kg) | 223650_s_at | NRBF2 | 7.61E-24 |
| rs74506613 | rs10740126 | 10 | 65210935 | 0.81 | LCL in asthmatics (Liang 1kg) | 223650_s_at | NRBF2 | 7.72E-24 |
| rs74506613 | rs7092784 | 10 | 65214749 | 0.81 | LCL in asthmatics (Liang 1kg) | 223650_s_at | NRBF2 | 8.17E-24 |
| rs74506613 | rs10761771 | 10 | 65230164 | 0.80 | LCL in asthmatics (Liang 1kg) | 223650_s_at | NRBF2 | 9.33E-24 |
| rs74506613 | rs10733792 | 10 | 65232539 | 0.81 | LCL in asthmatics (Liang 1kg) | 223650_s_at | NRBF2 | 9.37E-24 |
| rs74506613 | rs10761772 | 10 | 65235829 | 0.81 | LCL in asthmatics (Liang 1kg) | 223650_s_at | NRBF2 | 9.80E-24 |
| rs74506613 | rs7909960 | 10 | 65239177 | 0.81 | LCL in asthmatics (Liang 1kg) | 223650_s_at | NRBF2 | 1.01E-23 |
| rs74506613 | rs7915779 | 10 | 65244244 | 0.81 | LCL in asthmatics (Liang 1kg) | 223650_s_at | NRBF2 | 1.06E-23 |
| rs74506613 | rs2393977 | 10 | 65247609 | 0.81 | LCL in asthmatics (Liang 1kg) | 223650_s_at | NRBF2 | 1.11E-23 |
| rs74506613 | rs10740129 | 10 | 65250808 | 0.80 | LCL in asthmatics (Liang 1kg) | 223650_s_at | NRBF2 | 1.16E-23 |
| rs74506613 | rs10509189 | 10 | 65264126 | 0.81 | LCL in asthmatics (Liang 1kg) | 223650_s_at | NRBF2 | 1.40E-23 |
| rs74506613 | rs4486511 | 10 | 65264266 | 0.81 | LCL in asthmatics (Liang 1kg) | 223650_s_at | NRBF2 | 1.41E-23 |
| rs74506613 | rs10761778 | 10 | 65273782 | 0.81 | LCL in asthmatics (Liang 1kg) | 223650_s_at | NRBF2 | 1.74E-23 |
| rs74506613 | rs10761779 | 10 | 65274927 | 0.81 | CD14+ monocytes (24h LPS stimulated) | 6960523 | JMJD1C | 7.45E-05 |
| rs74506613 | rs10761779 | 10 | 65274927 | 0.81 | CD14+ monocytes (2h LPS stimulated) | 6960523 | JMJD1C | 1.04E-04 |
| rs74506613 | rs10761779 | 10 | 65274927 | 0.81 | CD14+ monocytes (IFNg stimulated) | 6960523 | JMJD1C | 1.93E-10 |
| rs74506613 | rs10761779 | 10 | 65274927 | 0.81 | CD14+ monocytes (untreated) | 6960523 | JMJD1C | 6.93E-07 |
| rs74506613 | rs7082470 | 10 | 65277026 | 0.81 | LCL in asthmatics (Liang 1kg) | 223650_s_at | NRBF2 | 1.84E-23 |
| rs74506613 | rs7920036 | 10 | 65293860 | 0.80 | LCL in asthmatics (Liang 1kg) | 223650_s_at | NRBF2 | 2.42E-23 |
| rs111939830 | rs609303 | 18 | 72895242 | 0.94 | Whole blood (Battle) |  | TSHZ1 | 5.86E-16 |
| rs111939830 | rs609303 | 18 | 72895242 | 0.94 | Bcells (CD19+) | WVR2Uc5UBXejngA5R8 | TSHZ1 | 2.23E-05 |
| *trans e-QTLs* |  |  |  |  |  |  |  |  |
| rs6993770 | rs6993770 | 8 | 106581528 | same SNP | Whole blood (CHARGE) | 540377 | CXCL5 | 1.28E-07 |
| rs74506613 | rs12355784 | 10 | 65121565 | 0.87 | Whole blood (CHARGE) | 2340577 | AQP10 | 2.15E-22 |
| rs74506613 | rs12355784 | 10 | 65121565 | 0.87 | Whole blood (CHARGE) | 380010 | CXCL5 | 1.90E-07 |
| rs74506613 | rs12355784 | 10 | 65121565 | 0.87 | Whole blood (CHARGE) | 4860681 | GUCY1A3 | 1.52E-07 |
| rs74506613 | rs12355784 | 10 | 65121565 | 0.87 | Whole blood (CHARGE) | 5340468 | ITGA2B | 1.76E-08 |
| rs74506613 | rs12355784 | 10 | 65121565 | 0.87 | Whole blood (CHARGE) | 5310437 | MYL9 | 4.13E-07 |
| rs74506613 | rs12355784 | 10 | 65121565 | 0.87 | Whole blood (CHARGE) | 6290747 | NRGN | 6.39E-09 |
| rs74506613 | rs10761779 | 10 | 65274927 | 0.81 | Whole blood (CHARGE) | 2340577 | AQP10 | 2.33E-22 |
| rs74506613 | rs10761779 | 10 | 65274927 | 0.81 | Whole blood (CHARGE) | 380010 | CXCL5 | 1.82E-07 |
| rs74506613 | rs10761779 | 10 | 65274927 | 0.81 | Whole blood (CHARGE) | 4860681 | GUCY1A3 | 1.95E-07 |
| rs74506613 | rs10761779 | 10 | 65274927 | 0.81 | Whole blood (CHARGE) | 5340468 | ITGA2B | 1.90E-08 |
| rs74506613 | rs10761779 | 10 | 65274927 | 0.81 | Whole blood (CHARGE) | 5310437 | MYL9 | 5.26E-07 |
| rs74506613 | rs10761779 | 10 | 65274927 | 0.81 | Whole blood (CHARGE) | 6290747 | NRGN | 6.90E-09 |

*eSNP.P: The p-values were stated in the original paper.
