# Supplementary material for: Six Novel Loci Associated with Circulating VEGF Levels Identified by a Meta-analysis of Genome-Wide Association Studies
Source: PLoS Genet. 2016 Feb 24;12(2):e1005874. doi: 10.1371/journal.pgen.1005874 (PMC4766012; doi:10.1371/journal.pgen.1005874)
Supplement: S4 Table — (DOCX) [file pgen.1005874.s006.docx]

**Supplementary Table4.**

| **Chromosomal region** | **Seed Genes** |
| --- | --- |
| 5q14.3 | *LINC00461* |
|  | *MEF2C* |
|  | *MEF2C-AS1* |
| 6p21.1 | *C6orf223* |
|  | *CAPN11* |
|  | *LOC100132354* |
|  | *MRPL14* |
|  | *MRPS18A* |
|  | *RSPH9* |
|  | *SLC29A1* |
|  | *TMEM63B* |
|  | *VEGFA* |
| 8p23.1 | *ZFPM2* |
| 9p24.2 | *KCNV2* |
|  | *KIAA0020* |
|  | *VLDLR* |
|  | *VLDLR-AS1* |
| 10p21.3 | *JMJD1C* |
|  | *JMJD1C-AS1* |
|  | *MIR1296* |
|  | *NRBF2* |
|  | *REEP3* |
| 16q24.2 | *MIR5189* |
|  | *ZFPM1* |
| 18q22.3 | *TSHZ1* |
|  | *ZADH2* |
