# Supplementary material for: Six Novel Loci Associated with Circulating VEGF Levels Identified by a Meta-analysis of Genome-Wide Association Studies
Source: PLoS Genet. 2016 Feb 24;12(2):e1005874. doi: 10.1371/journal.pgen.1005874 (PMC4766012; doi:10.1371/journal.pgen.1005874)
Supplement: S1 Text — (DOCX) [file pgen.1005874.s008.docx]

**Supporting information for**

**Six novel loci associated with circulating VEGF levels identified by a meta-analysis of genome-wide association studies**

**Contents:**

1. Participating Studies 2

2. VEGF Circulating Levels Measurement 4

3. eQTL Database 5

Supplementary Reference 6

**1. Participating Studies**

**AGES**

AGES stands for Age Gene/Environment Susceptibility Reykjavik Study^1^. The Reykjavik Study cohort originally comprised a random sample of 30,795 men and women born in 1907-1935 and living in Reykjavik in 1967. A total of 19,381 people attended, resulting in 71% recruitment rate. The study sample was divided into six groups by birth year and birth date within month. One group was designated for longitudinal follow up and was examined in all stages. One group was designated a control group and was not included in examinations until 1991. Other groups were invited to participate in specific stages of the study. Between 2002 and 2006, the AGES-Reykjavik study re-examined 5764 survivors of the original cohort who had participated earlier in the Reykjavik Study. All participants signed informed consent.

**Cilento study**

The Cilento study includes 2,137 individuals recruited through a population-based sampling strategy in three isolated villages: Campora, Cardile, and Gioi (*in–silico* replication sample in this work) located in the area of the National Park of Cilento e Vallo di Diano (South Italy)^2–4^. For each village a deep genealogy (15-17 generations) including the majority of current inhabitants was also reconstructed. The study aims to identify genetic risk factors for complex traits and diseases.

**Framingham Heart Study**

The Framingham Heart Study (FHS) is a three-generation, single-site, community-based, prospective cohort study that was initiated in 1948 to investigate risk factors for cardiovascular disease including stroke. It now comprises 3 generations of participants: the original cohort (N=5,209) followed since 1948 (Original cohort); their offspring and spouses of the offspring (N=5,124), followed since 1971 (Offspring cohort); and grandchildren of the Original cohort (N=4,095) followed since 2002 (Gen3 cohort). Vascular endothelial growth factor (VEGF) levels have been measured in Original, Offspring, and Gen 3 participants^5^.

**Ogliastra Genetic Park**

The Ogliastra Genetic Park Project is a cross-sectional population based study aimed at investigating genetic and environmental determinants of complex diseases^6–8^. The participants are inhabitants of ten villages in the Ogliastra region (Sardinia, Italy). Genealogy of participants has been reconstructed as far back as the founders living in the 17th century.

**Prospective Investigation of the Vasculature in Uppsala Seniors**

All 70-year old individuals living in the community of Uppsala, Sweden, between April 2001 and June 2004 were invited to the Prospective Investigation of the Vasculature in Uppsala Seniors (PIVUS) study^5,9^. The individuals were selected randomly, and were examined within one month of their 70th birthday in order to standardize for age. Of 2,025 individuals invited, 1,016 were investigated giving a participation rate of 50%.

**Val Borbera**

The cohort is a population-based study (n=1759) from an isolated population of a North West Italy valley. Participants, age 18-102, were selected to have at least one grandparent born in the valley. Detailed clinical and genealogical information and biological material was collected for all between 2005 and 2008.

**Sorbs**

All subjects are part of a sample from an extensively phenotyped self-contained population from Eastern Germany, the Sorbs (n=1020)^10,11^. The Sorbs are of Slavonic origin, and lived in ethnic isolation among the Germanic majority during the past 1100 years. Today, the Sorbian-speaking, Catholic minority comprises approximately 15,000 full-blooded Sorbs resident in about 10 villages in rural Upper Lusatia (Oberlausitz), Eastern Saxony.

**Hypertensive Adults**

The HT adults are a ‘case-only’ sample of hypertensive European Caucasians recruited between 1996 and 1998 (n=995). Inclusion criteria were systolic blood pressure (SBP) between 120 and 160 mmHg and diastolic blood pressure (DBP) between 95 and 115 mmHg or SBP between 160 and 240 mmHg and DBP less or equal to 95 mmHg.

**STANISLAS Family Study**

The STANISLAS Family Study (SFS) is a 10-year, longitudinal survey involving 1006 volunteer families from Vandoeuvre-lès- Nancy, France, whose members were free of chronic disease (cardiovascular or cancer) between 1993 and 1995^5,12,13^. Plasma VEGF levels were measured at the second examination cycle (1998 to 2000) in a randomly selected subsample of 676 persons from 192 families, who also had DNA and met genotyping quality control criteria.

**2. VEGF Circulating Levels Measurement**

Blood samples in AGES were collected in the morning after the participants had been fasting for at least 12 h. Aliquots of serum were immediately prepared and stored at −80°C, and were subsequently used for the assessment of VEGF levels. VEGF was measured using solid phase ELISA, according to the manufacturer's instructions (Quantikine™, R&D Systems, Minneapolis, MN). Detection limit 9.0 pg/mL. Range 31.2-2000 pg/L, Inter- and intra-assay variability: 8% and 5.5% respectively.

Blood samples in Cilento, VB and Gioi were collected in the morning following a 12 h fasting. Aliquots of serum were immediately prepared and stored at −80°C, and were subsequently used for the assessment of VEGF levels. VEGF was measured using an enzyme-linked immunosorbent assay, according to the manufacturer's instructions (Quantikine™, R&D Systems, Minneapolis, MN). For Cilento, 32 individuals out of the 1147 who underwent genotyping, were excluded because they did not have VEGF serum levels. In total, 1115 individuals have both VEGF serum levels and genotyping information. For VB, 26 individuals out of the 1785 participants who underwent genotyping, were excluded because they did not have VEGF serum levels. In total, 1759 individuals have both VEGF serum levels and genotyping information. In Gioi all the 470 individuals that underwent genotyping were included in the study.

Blood VEGF levels in FHS were measured in serum. Blood samples were drawn after an overnight fast, immediately centrifuged and stored appropriately (at -80°C) until VEGF measurements were undertaken. Serum VEGF was measured using a commercial ELISA assay (Quantikine™, R&D Systems, Minneapolis, MN.). Of 8384 participants who have available genotype data, 1336 individuals were excluded because they did not have VEGF serum levels and do not have high quality genome-wide genotyping data. In total, 7048 individuals are available.

Blood samples in Ogliastra Genetic Park were collected in the morning after the participants had been fasting for at least 12 h. Aliquots of serum were immediately prepared and stored at −80°C, and were subsequently used for the assessment of VEGF levels. VEGF was measured using an enzyme-linked immunosorbent assay, according to the manufacturer's instructions (Duoset R&D System, Minneapolis, MN). Of the 1164 genotyped individuals, 267 were excluded because they did not have VEGF serum levels. In total 897 individuals have both VEGF serum levels and genotyping information.

Blood samples in PIVUS were drawn after an overnight fasting, immediately centrifuged and stored appropriately at -80°C until assessment of serum VEGF levels. Serum VEGF quantification was performed by Randox Ltd (Crumlin, UK), using a biochip array analyzer (Evidence®).

Blood samples in Sorbs were collected in the morning after overnight fasting. Serum was spinned and frozen immediately and further stored at −80°C. and were subsequently used for the assessment of VEGF levels. VEGF was measured using an enzyme-linked immunosorbent assay, according to the manufacturer's instructions (Quantikine™, R&D Systems, Minneapolis, MN).

Venous blood samples in HT and SFS were drawn after an overnight fast, immediately centrifuged and stored appropriately (-196°C in liquid nitrogen) until VEGF measurements were undertaken. Plasma VEGF quantification was performed by Randox Ltd (Crumlin, UK), using a biochip array analyzer (Evidence®).

**3. eQTL Database**

Blood cell related eQTL studies included fresh lymphocytes^14^, fresh leukocytes^15^, leukocyte samples in individuals with Celiac disease^16^, whole blood samples^17–28^,lymphoblastoid cell lines (LCL) derived from asthmatic children^29,30^, HapMap LCL from 3 populations^31^, a separate study on HapMap CEU LCL^32^, additional LCL population samples^33–37^, CD19+ B cells^38^, primary PHA-stimulated T cells^33,36^, CD4+ T cells^39^, peripheral blood monocytes^38,40,41^and CD14+ monocytes before and after stimulation with LPS or interferon-gamma^42^, CD11+ dendritic cells before and after *Mycobacterium tuberculosis* infection^43^ and a separate study of dendritic cells before or after stimulation with LPS, influenza or interferon-beta^44^. Micro-RNA QTLs^45^ and DNase-I QTLs^46^ were also queried for LCL.

Non-blood cell tissue eQTLs searched included omental and subcutaneous adipose^17,25,35,47^, stomach^47^, endometrial carcinomas^48^, ER+ and ER- breast cancer tumor cells^49^, brain cortex^40,50,51^, gliomas^52^, pre-frontal cortex^53–55^, parietal lobe^56^, frontal cortex^55,57^, temporal cortex^51,55,57^, hippocampus^55^, thalamus^55^, pons^57^, cerebellum^51,55–57^, 3 additional large studies of brain regions including prefrontal cortex, visual cortex and cerebellum, respectively^58^, liver^47,59–62^, osteoblasts^63^, intestine^64^, skeletal muscle^65^, breast tissue (normal and cancer)^66,67^, lung^25,67,68^, skin^25,35,69^, primary fibroblasts^33,36^, sputum^70^, and heart tissue from left ventricles^25,71^and left and right atria^72^. Micro-RNA QTLs were also queried for gluteal and abdominal adipose^73^. Further mRNA and micro-RNA QTLs were queried from ER+ invasive breast cancer samples, colon, kidney renal clear, lung and prostate adenocarcinoma samples^74^.

Additional eQTL data was integrated from online sources including ScanDB, the Broad Institute GTex browser, and the Pritchard Lab (eqtl.uchicago.edu). Cerebellum, parietal lobe and liver eQTL data was downloaded from ScanDB and cis-eQTLs were limited to those with P<1.0E-6 and trans-eQTLs with P<5.0E-8. The top 1000 eQTL results were downloaded from the GTex Browser at the Broad Institute for 9 tissues on 11/26/2013: thyroid, leg skin (sun exposed), tibial nerve, tibial artery, skeletal muscle, lung, heart (left ventricle), whole blood, and subcutaneous adipose^25^. All GTex results had associations with P<8.4x10^-07^.

**Supplementary Reference**

1. Harris TB, Launer LJ, Eiriksdottir G, Kjartansson O, Jonsson PV, Sigurdsson G, Thorgeirsson G, Aspelund T, Garcia ME, Cotch MF, Hoffman HJ, Gudnason V. Age, Gene/Environment Susceptibility-Reykjavik Study: multidisciplinary applied phenomics. *Am J Epidemiol*. 2007;165(9):1076–87.

2. Colonna V, Nutile T, Ferrucci RR, Fardella G, Aversano M, Barbujani G, Ciullo M. Comparing population structure as inferred from genealogical versus genetic information. *Eur J Hum Genet*. 2009;17(12):1635–41.

3. Colonna V, Nutile T, Astore M, Guardiola O, Antoniol G, Ciullo M, Persico MG. Campora: a young genetic isolate in South Italy. *Hum Hered*. 2007;64(2):123–35.

4. Ruggiero D, Dalmasso C, Nutile T, Sorice R, Dionisi L, Aversano M, Bröet P, Leutenegger AL, Bourgain C, Ciullo M. Genetics of VEGF serum variation in human isolated populations of cilento: importance of VEGF polymorphisms. *PLoS One*. 2011;6(2):e16982.

5. Debette S, Visvikis-Siest S, Chen M-H, et al. Identification of cis- and trans-acting genetic variants explaining up to half the variation in circulating vascular endothelial growth factor levels. *Circ Res*. 2011;109(5):554–63.

6. Pistis G, Piras I, Pirastu N, Persico I, Sassu A, Picciau A, Prodi D, Fraumene C, Mocci E, Manias MT, Atzeni R, Cosso M, Pirastu M, Angius A. High differentiation among eight villages in a secluded area of Sardinia revealed by genome-wide high density SNPs analysis. *PLoS One*. 2009;4(2):e4654.

7. Fraumene C, Belle EMS, Castrì L, Sanna S, Mancosu G, Cosso M, Marras F, Barbujani G, Pirastu M, Angius A. High resolution analysis and phylogenetic network construction using complete mtDNA sequences in sardinian genetic isolates. *Mol Biol Evol*. 2006;23(11):2101–11.

8. Fraumene C, Petretto E, Angius A, Pirastu M. Striking differentiation of sub-populations within a genetically homogeneous isolate (Ogliastra) in Sardinia as revealed by mtDNA analysis. *Hum Genet*. 2003;114(1):1–10.

9. Lind L, Fors N, Hall J, Marttala K, Stenborg A. A comparison of three different methods to evaluate endothelium-dependent vasodilation in the elderly: the Prospective Investigation of the Vasculature in Uppsala Seniors (PIVUS) study. *Arterioscler Thromb Vasc Biol*. 2005;25(11):2368–75.

10. Tönjes A, Koriath M, Schleinitz D, et al. Genetic variation in GPR133 is associated with height: genome wide association study in the self-contained population of Sorbs. *Hum Mol Genet*. 2009;18(23):4662–8.

11. Tönjes A, Zeggini E, Kovacs P, et al. Association of FTO variants with BMI and fat mass in the self-contained population of Sorbs in Germany. *Eur J Hum Genet*. 2010;18(1):104–10.

12. Visvikis-Siest S, Siest G. The STANISLAS Cohort: a 10-year follow-up of supposed healthy families. Gene-environment interactions, reference values and evaluation of biomarkers in prevention of cardiovascular diseases. *Clin Chem Lab Med*. 2008;46(6):733–47.

13. Siest G, Visvikis S, Herbeth B, Gueguen R, Vincent-Viry M, Sass C, Beaud B, Lecomte E, Steinmetz J, Locuty J, Chevrier P. Objectives, design and recruitment of a familial and longitudinal cohort for studying gene-environment interactions in the field of cardiovascular risk: the Stanislas cohort. *Clin Chem Lab Med*. 1998;36(1):35–42.

14. Göring HHH, Curran JE, Johnson MP, et al. Discovery of expression QTLs using large-scale transcriptional profiling in human lymphocytes. *Nat Genet*. 2007;39(10):1208–16.

15. Idaghdour Y, Czika W, Shianna K V, Lee SH, Visscher PM, Martin HC, Miclaus K, Jadallah SJ, Goldstein DB, Wolfinger RD, Gibson G. Geographical genomics of human leukocyte gene expression variation in southern Morocco. *Nat Genet*. 2010;42(1):62–7.

16. Heap GA, Trynka G, Jansen RC, Bruinenberg M, Swertz MA, Dinesen LC, Hunt KA, Wijmenga C, Vanheel DA, Franke L. Complex nature of SNP genotype effects on gene expression in primary human leucocytes. *BMC Med Genomics*. 2009;2:1.

17. Emilsson V, Thorleifsson G, Zhang B, et al. Genetics of gene expression and its effect on disease. *Nature*. 2008;452(7186):423–8.

18. Fehrmann RSN, Jansen RC, Veldink JH, et al. Trans-eQTLs reveal that independent genetic variants associated with a complex phenotype converge on intermediate genes, with a major role for the HLA. *PLoS Genet*. 2011;7(8):e1002197.

19. Mehta D, Heim K, Herder C, Carstensen M, Eckstein G, Schurmann C, Homuth G, Nauck M, Völker U, Roden M, Illig T, Gieger C, Meitinger T, Prokisch H. Impact of common regulatory single-nucleotide variants on gene expression profiles in whole blood. *Eur J Hum Genet*. 2013;21(1):48–54.

20. Zhernakova D V, de Klerk E, Westra H-J, et al. DeepSAGE reveals genetic variants associated with alternative polyadenylation and expression of coding and non-coding transcripts. *PLoS Genet*. 2013;9(6):e1003594.

21. Sasayama D, Hori H, Nakamura S, Miyata R, Teraishi T, Hattori K, Ota M, Yamamoto N, Higuchi T, Amano N, Kunugi H. Identification of single nucleotide polymorphisms regulating peripheral blood mRNA expression with genome-wide significance: an eQTL study in the Japanese population. *PLoS One*. 2013;8(1):e54967.

22. Landmark-Høyvik H, Dumeaux V, Nebdal D, Lund E, Tost J, Kamatani Y, Renault V, Børresen-Dale AL, Kristensen V, Edvardsen H. Genome-wide association study in breast cancer survivors reveals SNPs associated with gene expression of genes belonging to MHC class I and II. *Genomics*. 2013;102(4):278–87.

23. Westra H-J, Peters MJ, Esko T, et al. Systematic identification of trans eQTLs as putative drivers of known disease associations. *Nat Genet*. 2013;45(10):1238–43.

24. Van Eijk KR, de Jong S, Boks MPM, Langeveld T, Colas F, Veldink JH, de Kovel CG, Janson E, Strengman E, Langfelder P, Kahn RS, van den Berg LH, Horvath S, Ophoff RA.. Genetic analysis of DNA methylation and gene expression levels in whole blood of healthy human subjects. *BMC Genomics*. 2012;13:636.

25. Lonsdale J, Thomas J, Salvatore M, et al. The Genotype-Tissue Expression (GTEx) project. *Nat Genet*. 2013;45(6):580–5.

26. Battle A, Mostafavi S, Zhu X, Potash JB, Weissman MM, McCormick C, Haudenschild CD, Beckman KB, Shi J, Mei R, Urban AE, Montgomery SB, Levinson DF, Koller D. Characterizing the genetic basis of transcriptome diversity through RNA-sequencing of 922 individuals. *Genome Res*. 2014;24(1):14–24.

27. Benton MC, Lea RA, Macartney-Coxson D, Carless MA, Göring HH, Bellis C, Hanna M, Eccles D, Chambers GK, Curran JE, Harper JL, Blangero J, Griffiths LR. Mapping eQTLs in the Norfolk Island genetic isolate identifies candidate genes for CVD risk traits. *Am J Hum Genet*. 2013;93(6):1087–99.

28. Narahara M, Higasa K, Nakamura S, Tabara Y, Kawaguchi T, Ishii M, Matsubara K, Matsuda F, Yamada R. Large-scale East-Asian eQTL mapping reveals novel candidate genes for LD mapping and the genomic landscape of transcriptional effects of sequence variants. *PLoS One*. 2014;9(6):e100924.

29. Dixon AL, Liang L, Moffatt MF, Chen W, Heath S, Wong KC, Taylor J, Burnett E, Gut I, Farrall M, Lathrop GM, Abecasis GR, Cookson WO. A genome-wide association study of global gene expression. *Nat Genet*. 2007;39(10):1202–1207.

30. Liang L, Morar N, Dixon AL, Lathrop GM, Abecasis GR, Moffatt MF, Cookson WO. A cross-platform analysis of 14,177 expression quantitative trait loci derived from lymphoblastoid cell lines. *Genome Res*. 2013;23(4):716–726.

31. Stranger BE, Nica AC, Forrest MS, Dimas A, Bird CP, Beazley C, Ingle CE, Dunning M, Flicek P, Koller D, Montgomery S, Tavaré S, Deloukas P, Dermitzakis ET. Population genomics of human gene expression. *Nat Genet*. 2007;39(10):1217–24.

32. Kwan T, Benovoy D, Dias C, Gurd S, Provencher C, Beaulieu P, Hudson TJ, Sladek R, Majewski J. Genome-wide analysis of transcript isoform variation in humans. *Nat Genet*. 2008;40(2):225–231.

33. Dimas AS, Deutsch S, Stranger BE, et al. Common regulatory variation impacts gene expression in a cell type-dependent manner. *Science*. 2009;325(5945):1246–50.

34. Cusanovich DA, Billstrand C, Zhou X, Chavarria C, De Leon S, Michelini K, Pai AA, Ober C, Gilad Y. The combination of a genome-wide association study of lymphocyte count and analysis of gene expression data reveals novel asthma candidate genes. *Hum Mol Genet*. 2012;21(9):2111–2123.

35. Grundberg E, Small KS, Hedman AK, et al. Mapping cis- and trans-regulatory effects across multiple tissues in twins. *Nat Genet*. 2012;44(10):1084–1089.

36. Gutierrez-Arcelus M, Lappalainen T, Montgomery SB, et al. Passive and active DNA methylation and the interplay with genetic variation in gene regulation. *Elife*. 2013;2:e00523.

37. Mangravite LM, Engelhardt BE, Medina MW, et al. A statin-dependent QTL for GATM expression is associated with statin-induced myopathy. *Nature*. 2013;502(7471):377–80.

38. Fairfax BP, Makino S, Radhakrishnan J, Plant K, Leslie S, Dilthey A, Ellis P, Langford C, Vannberg FO, Knight JC. Genetics of gene expression in primary immune cells identifies cell type-specific master regulators and roles of HLA alleles. *Nat Genet*. 2012;44(5):502–510.

39. Murphy A, Chu J-HH, Xu M, et al. Mapping of numerous disease-associated expression polymorphisms in primary peripheral blood CD4+ lymphocytes. *Hum Mol Genet*. 2010;19(23):4745–4757.

40. Heinzen EL, Ge D, Cronin KD, Maia JM, Shianna KV, Gabriel WN, Welsh-Bohmer KA, Hulette CM, Denny TN, Goldstein DB. Tissue-specific genetic control of splicing: implications for the study of complex traits. *PLoS Biol*. 2008;6(12):e1.

41. Zeller T, Wild P, Szymczak S, et al. Genetics and beyond--the transcriptome of human monocytes and disease susceptibility. *PLoS One*. 2010;5(5):e10693.

42. Fairfax BP, Humburg P, Makino S, Naranbhai V, Wong D, Lau E, Jostins L, Plant K, Andrews R, McGee C, Knight JC. Innate immune activity conditions the effect of regulatory variants upon monocyte gene expression. *Science*. 2014;343(6175):1246949.

43. Barreiro LB, Tailleux L, Pai AA, Gicquel B, Marioni JC, Gilad Y. Deciphering the genetic architecture of variation in the immune response to Mycobacterium tuberculosis infection. *Proc Natl Acad Sci U S A*. 2012;109(4):1204–1209.

44. Lee MNHMN, Ye C, Villani A-CC, et al. Common genetic variants modulate pathogen-sensing responses in human dendritic cells. *Science*. 2014;343(6175):1246980.

45. Huang RS, Gamazon ER, Ziliak D, Wen Y, Im HK, Zhang W, Wing C, Duan S, Bleibel WK, Cox NJ, Dolan ME. Population differences in microRNA expression and biological implications. *RNA Biol*. 2011;8(4):692–701.

46. Degner JF, Pai AA, Pique-Regi R, Veyrieras JB, Gaffney DJ, Pickrell JK, De Leon S, Michelini K, Lewellen N, Crawford GE, Stephens M, Gilad Y, Pritchard JK. DNase I sensitivity QTLs are a major determinant of human expression variation. *Nature*. 2012;482(7385):390–4.

47. Greenawalt DM, Dobrin R, Chudin E, et al. A survey of the genetics of stomach, liver, and adipose gene expression from a morbidly obese cohort. *Genome Res*. 2011;21(7):1008–1016.

48. Kompass KS, Witte JS. Co-regulatory expression quantitative trait loci mapping: method and application to endometrial cancer. *BMC Med Genomics*. 2011;4:6.

49. Li Q, Seo J-HH, Stranger B, McKenna A, Pe'er I, Laframboise T, Brown M, Tyekucheva S, Freedman ML. Integrative eQTL-based analyses reveal the biology of breast cancer risk loci. *Cell*. 2013;152(3):633–641.

50. Webster JA, Gibbs JR, Clarke J, et al. Genetic control of human brain transcript expression in Alzheimer disease. *Am J Hum Genet*. 2009;84(4):445–458.

51. Zou F, Chai HS, Younkin CS, et al. Brain expression genome-wide association study (eGWAS) identifies human disease-associated variants. *PLoS Genet*. 2012;8(6):e1002707.

52. Shpak M, Hall AW, Goldberg MM, et al. An eQTL analysis of the human glioblastoma multiforme genome. *Genomics*. 2014;103(4):252–263.

53. Colantuoni C, Lipska BK, Ye T, Hyde TM, Tao R, Leek JT, Colantuoni EA, Elkahloun AG, Herman MM, Weinberger DR, Kleinman JE. Temporal dynamics and genetic control of transcription in the human prefrontal cortex. *Nature*. 2011;478(7370):519–523.

54. Liu C, Cheng L, Badner JA, Zhang D, Craig DW, Redman M, Gershon ES. Whole-genome association mapping of gene expression in the human prefrontal cortex. *Mol Psychiatry*. 2010;15(8):779–784.

55. Kim S, Cho H, Lee D, Webster MJ. Association between SNPs and gene expression in multiple regions of the human brain. *Transl Psychiatry*. 2012;2:e113.

56. Gamazon ER, Badner JA, Cheng L, et al. Enrichment of cis-regulatory gene expression SNPs and methylation quantitative trait loci among bipolar disorder susceptibility variants. *Mol Psychiatry*. 2013;18(3):340–346.

57. Gibbs JR, van der Brug MP, Hernandez DG, et al. Abundant quantitative trait loci exist for DNA methylation and gene expression in human brain. *PLoS Genet*. 2010;6(5):e1000952.

58. Zhang B, Gaiteri C, Bodea L-GG, et al. Integrated systems approach identifies genetic nodes and networks in late-onset Alzheimer’s disease. *Cell*. 2013;153(3):707–720.

59. Schadt EE, Molony C, Chudin E, et al. Mapping the genetic architecture of gene expression in human liver. *PLoS Biol*. 2008;6(5):e107.

60. Innocenti F, Cooper GM, Stanaway IB, et al. Identification, replication, and functional fine-mapping of expression quantitative trait loci in primary human liver tissue. *PLoS Genet*. 2011;7(5):e1002078.

61. Schroder A, Klein K, Winter S, Schwab M, Bonin M, Zell A, Zanger UM. Genomics of ADME gene expression: mapping expression quantitative trait loci relevant for absorption, distribution, metabolism and excretion of drugs in human liver. *Pharmacogenomics J*. 2013;13(1):12–20.

62. Wang X, Tang H, Teng M, et al. Mapping of hepatic expression quantitative trait loci (eQTLs) in a Han Chinese population. *J Med Genet*. 2014;51(5):319–326.

63. Grundberg E, Kwan T, Ge B, et al. Population genomics in a disease targeted primary cell model. *Genome Res*. 2009;19(11):1942–1952.

64. Kabakchiev B, Silverberg MS. Expression quantitative trait loci analysis identifies associations between genotype and gene expression in human intestine. *Gastroenterology*. 2013;144(7):1488–96, 1496.e1–3.

65. Keildson S, Fadista J, Ladenvall C, et al. Expression of phosphofructokinase in skeletal muscle is influenced by genetic variation and associated with insulin sensitivity. *Diabetes*. 2014;63(3):1154–1165.

66. Quigley DA, Fiorito E, Nord S, et al. The 5p12 breast cancer susceptibility locus affects MRPS30 expression in estrogen-receptor positive tumors. *Mol Oncol*. 2014;8(2):273–284.

67. Curtis C, Shah SP, Chin S-FF, et al. The genomic and transcriptomic architecture of 2,000 breast tumours reveals novel subgroups. *Nature*. 2012;486(7403):346–352.

68. Gao C, Tignor NL, Salit J, Strulovici-Barel Y, Hackett NR, Crystal RG, Mezey JG. HEFT: eQTL analysis of many thousands of expressed genes while simultaneously controlling for hidden factors. *Bioinformatics*. 2014;30(3):369–376.

69. Ding J, Gudjonsson JE, Liang L, Stuart PE, Li Y, Chen W, Weichenthal M, Ellinghaus E, Franke A, Cookson W, Nair RP, Elder JT, Abecasis GR. Gene expression in skin and lymphoblastoid cells: Refined statistical method reveals extensive overlap in cis-eQTL signals. *Am J Hum Genet*. 2010;87(6):779–789.

70. Qiu W, Cho MH, Riley JH, et al. Genetics of sputum gene expression in chronic obstructive pulmonary disease. *PLoS One*. 2011;6(9):e24395.

71. Koopmann TT, Adriaens ME, Moerland PD, et al. Genome-wide identification of expression quantitative trait loci (eQTLs) in human heart. *PLoS One*. 2014;9(5):e97380.

72. Lin H, Dolmatova E V, Morley MP, Lunetta KL, McManus DD, Magnani JW, Margulies KB, Hakonarson H, del Monte F, Benjamin EJ, Cappola TP, Ellinor PT. Gene expression and genetic variation in human atria. *Heart Rhythm*. 2014;11(2):266–271.

73. Rantalainen M, Herrera BM, Nicholson G, et al. MicroRNA expression in abdominal and gluteal adipose tissue is associated with mRNA expression levels and partly genetically driven. *PLoS One*. 2011;6(11):e27338.

74. Li Q, Stram A, Chen C, Kar S, Gayther S, Pharoah P, Haiman C, Stranger B, Kraft P, Freedman ML. Expression QTL-based analyses reveal candidate causal genes and loci across five tumor types. *Hum Mol Genet*. 2014;23(19):5294–5302.
